# Cryogels Based on Poly(2-oxazoline)s through Development of Bi- and Trifunctional Cross-Linkers Incorporating End Groups with Adjustable Stability

**DOI:** 10.1021/acs.macromol.3c02030

**Published:** 2024-03-12

**Authors:** Nora Engel, Tim Hoffmann, Florian Behrendt, Phil Liebing, Christine Weber, Michael Gottschaldt, Ulrich S. Schubert

**Affiliations:** † Laboratory of Organic Chemistry and Macromolecular Chemistry (IOMC), Friedrich Schiller University at Jena, Humboldtstraße 10, 07743 Jena, Germany; ‡ Jena Center for Soft Matter (JCSM), Friedrich Schiller University Jena, Philosophenweg 7, 07743 Jena, Germany; § Institute of Inorganic and Analytical Chemistry (IAAC), Friedrich Schiller University at Jena, Humboldtstraße 8, 07743 Jena, Germany

## Abstract

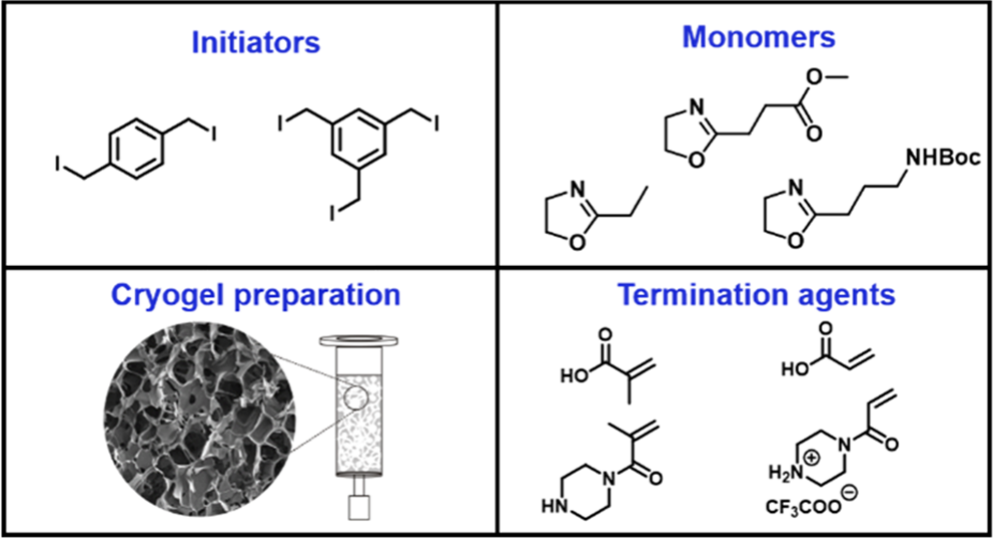

1,4-Bis(iodomethyl)benzene and 1,3,5-tris(iodomethyl)benzene were used as initiators for the cationic ring-opening polymerization (CROP) of 2-ethyl-2-oxazoline (EtOx) and its copolymerization with *tert*-butyl (3-(4,5-dihydrooxazol-2-yl)propyl)carbamate (BocOx) or methyl 3-(4,5-dihydrooxazol-2-yl)propanoate (MestOx). Kinetic studies confirmed the applicability of these initiators. Termination with suitable nucleophiles resulted in two- and three-armed cross-linkers featuring acrylate, methacrylate, piperazine-acrylamide, and piperazine-methacrylamide as polymerizable ω-end groups. Matrix-assisted laser desorption/ionization mass spectrometry and nuclear magnetic resonance (NMR) spectroscopy confirmed the successful attachment of the respective ω-end groups at all initiation sites for every prepared cross-linkers. Except for acrylate, each ω-end group remained stable during deprotection of BocOx containing cross-linkers. The cryogels were prepared using EtOx-based cross-linkers, as confirmed by solid-state NMR spectroscopy, scanning electron microscopy, and thermogravimetric analysis. Stability tests revealed a complete dissolution of the acrylate-containing gels at pH = 14, whereas the piperazine-acrylamide-based cryogels featured excellent hydrolytic stability.

## 1 Introduction

Cryogels represent a class of three-dimensional (3D)-structured materials consisting of cross-linked polymer networks. In contrast to hydrogels, these materials are characterized by an interconnected pore structure with enhanced mechanical properties enabling mass-transport of substances.^1,2^ Typically, cryogels are applied for controlled drug delivery,^3^ tissue engineering,^4^ as bioreactors,^5^ for heavy metal adsorption,^6^ for separation purposes,^7^ and also as 3D scaffolds for the cultivation of cells and microorganisms.^8,9^ Most commonly, *N*,*N*′-methylenebis(acrylamide) (MBAAm),^10,11^ ethylene glycol dimethacrylate (EGDMA),^12,13^ poly(ethylene glycol) diacrylate (PEGDA),^14,15^ and poly(ethylene glycol) dimethacrylate (PEGDMA)^16,17^ are used as cross-linking agents for the preparation of synthetic polymer-based cryogels via free-radical cross-linking polymerization at subzero temperatures. In particular, the poly(ethylene glycol) (PEG)-based cross-linker are frequently applied when biomedical applications are targeted.^14,15,17^ As an example, cryogels based on poly(ethylene glycol) methyl ether methacrylate, PEGMA, and 2-(*N*-succinimidylcarboxyoxy)ethyl methacrylate were prepared and functionalized with the amine-containing anticancer drug doxorubicin.^18^

Hydrophilic poly(2-oxazoline)s (POx) are currently of high interest as PEG alternatives in that context.^19,20^ Polymerizable end groups can be easily attached due to the living nature of the cationic ring-opening polymerization (CROP), which is used to obtain POx. Termination of the living oxazolinium species by acrylate as well as methacrylate nucleophiles has been reported in the 1980′s^21,22^ and has since been often applied.^23−26^ Alternatively, the CROP can be terminated by *N*-*tert*-butyloxycarbonylpiperazine,^27−30^ which has been exploited for further postpolymerization end group modifications.^27,29^ The termination with piperazine-based amines as nucleophiles also allows to attach radically polymerizable moieties in a direct fashion, as reported by Rueda et al., who used *N*-(4-vinylbenzyl)piperazine as a CROP termination agent.^31^

The use of bi- or multifunctional CROP initiators enables access to polymers or oligomers bearing two or more radically polymerizable ω-end groups.^32^ Those have been applied as cross-linkers for the preparation of films,^33,34^ hydrogels,^26,35,36^ microbeads,^37^ coatings,^38^ or nanofibers^39^ and remain of high interest to date. However, their potential has rarely been exploited with respect to cryogels. To the best of our knowledge, only one POx containing cryogel has been reported,^40^ which is mainly based on *N,N-*dimethylacrylamide and only contains 14 mol % of poly(2-ethyl-2-oxazoline) diacrylate (PEtOxDA).

Cryogels prepared from solely cross-linkers are rare, although PEGDA has been applied for that purpose.^15,41^ It is more common to adjust the cross-linker density and cryogel properties by copolymerization with low molar mass acrylate or methacrylate monomers. However, adjustment of the cross-linker density could also be realized by the use of multifunctional cross-linkers, e.g., by introduction of oxazoline monomers to serve as radically polymerizable moieties.^42^ In addition, the use of bi- or multifunctional CROP initiators such as 1,4-dibromo-2-butene,^43,44^ 1,4-bis(bromomethyl)benzene^45^ or 1,4-bis(iodomethyl)benzene (**BIB**) in combination with functional termination agents to yield polymerizable ω-end groups appears convenient. A comprehensive review of star-shaped POx using a variety of multifunctional initiators, including bi- and trifunctional initiators, has been published recently.^46^ Many multifunctional CROP initiators suffer from sterical hindrance and slow initiation,^46,47^ a drawback that can be overcome by enhancing the leaving group quality at the initiator.^48^ It is hence surprising that the approach has not yet been investigated for multiple initiators based on benzyl halides, whose reactivity can be increased by exchanging the bromide by iodide.^49^

The use of low molar mass comonomers enables the introduction of moieties for the additional functionalization of cryogels. However, such groups can also be already present in a suitably designed cross-linker. As prominent examples, such as carboxylic acids and primary amines, are not tolerated by the CROP mechanism, they have to be introduced by monomers bearing suitable protection groups. Methyl 3-(4,5-dihydrooxazol-2-yl)propanoate (MestOx)^50^ contains a carboxylic acid methylester, which can be functionalized directly by amidation without prior deprotection.^51^ [3-[(*tert*-Butoxycarbonyl)amino]propyl]-4,5-dihydrooxazole (BocOx)^52^ features a Boc-protected primary amino group. Once deprotected after polymerization of the monomer, BocOx provides access to subsequent functionalization with, e.g., molecules for targeting purposes^53^ or dyes.^54^

**Scheme 1:**
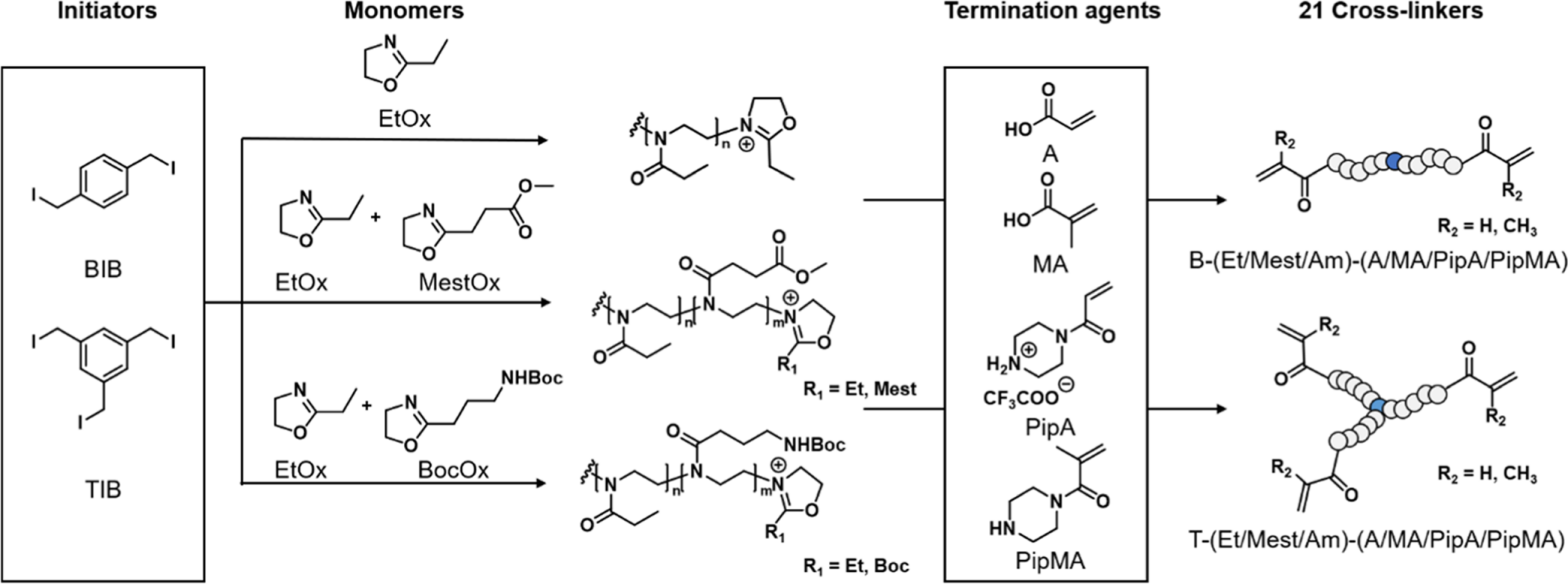
Schematic Representation of the Synthesis Route of the POx-Based Cross-Linkers Using **BIB** or **TIB** an as Initiator EtOx, MestOx, and BocOx were used as monomers. Acrylic acid (A), methacrylic acid (MA), piperazine-acrylamide in the form of its trifluoroacetic acid salt (**PipA**), and piperazine-methacrylamide (**PipMA**) were used as termination agents.

We present the development of cryogels based on purely POx by exploring difunctional CROP initiator **BIB** as well as trifunctional initiator 1,3,5-tris(iodomethyl)benzene (**TIB**). Aiming toward cryogels with varied hydrolytic stability, polymerizable ω-end groups based on acrylate, methacrylate, acrylamide, and methacrylamide were attached via direct termination of the CROP (Scheme 1). In addition to homopolymerization of EtOx to result in hydrophilic cross-linkers, the introduction of further functional moieties was tackled by copolymerization with MestOx as well as BocOx. The concept was proven by preparation of cryogels based on PEtOx, which were characterized by scanning electron microscopy (SEM), thermogravimetric analysis (TGA), and solid-state nuclear magnetic resonance (ssNMR) spectroscopy.

## 2 Experimental Section

Detailed information concerning the materials and instruments as well as procedures describing the synthesis of initiators, monomers, and termination agents can be found in the Supporting Information.

### 2.1 General Procedure for Kinetic Studies

All kinetic studies were performed at an overall [M]_0_ = 2 mol L^–1^ in acetonitrile. The ratio of [initiating moieties] to [M]_0_ was kept constant at 5. This resulted in a [M]_0_/[I]_0_ ratio of 10 when the bifunctional initiator **BIB** was used, whereas [M]_0_/[I]_0_ = 15 for the trifunctional initiator **TIB**. In copolymerizations, 68 mol % of EtOx and 32 mol % of MestOx or BocOx, respectively, were used. Detailed amounts are provided in the Supporting Information.

A preheated flask was cooled to room temperature under argon. Subsequent to heating under vacuum refilling with argon thrice, the flask was charged with initiator, i.e., **BIB** or **TIB**, respectively, under an inert atmosphere. The predetermined amounts of monomer were added, i.e., EtOx, EtOx and MestOx or BocOx and EtOx, respectively. Subsequent to the addition of acetonitrile, the reaction mixture was heated to reflux using a heat-on system under a continuous gentle argon flow. 100 μL aliquots were taken at preselected time points (5, 10, 15, 30, 45, 60, 90, 120, and 150 min, respectively) to determine the monomer conversion via gas chromatography (GC) and the molar mass distribution via size exclusion chromatography (SEC).

The polymerization rate coefficient *k*_p_ of the corresponding monomer was calculated by the postulation of a linear fit according to eqs 1 and 2.
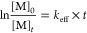
1
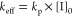
2The *k*_p_ values per side chain were calculated by applying eqs 3 and 4. The resulting values are summarized in Table 1
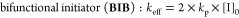
3
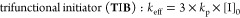
4

**Table 1:** Apparent Polymerization Rate Coefficients *k*_p_ and Apparent Polymerization Coefficients per Initiation Site *k*_p,arm_ Given in L mol^–1^ min^–1^ Calculated from the Slope of the Semi-Logarithmic Kinetic Plots According to Equations 2–4

polymer	monomer	*k*_p_ [L mol^–1^ min^–1^]	*k*_p,arm_ [L mol^−1^ min^–1^]
**B-EtOx**	EtOx	0.168	0.084
**B-MestOx**	EtOx	0.172	0.086
	MestOx	0.084	0.042
**B-BocOx**	EtOx	0.160	0.080
	BocOx	0.142	0.071
**T-EtOx**	EtOx	0.257	0.086
**T-MestOx**	EtOx	0.498	0.162
	MestOx	0.209	0.070
**T-BocOx**	EtOx	0.188	0.063
	BocOx	0.183	0.061

### 2.2 General Procedure for the Cross-Linker Synthesis

The cross-linker molecules were synthesized in a similar fashion as described above for the kinetic studies. Instead of sampling during the CROP, the reactions were terminated with triethylammonium acrylate, triethylammonium methacrylate, *N*-acryloyl-piperazinium trifluoroacetate (**PipA**) or *N*-methacryloyl-piperazine (**PipMA**). Detailed amounts and purification procedures for the termination agents and for each cross-linker are provided in the Supporting Information. The molecular structure of **PipA** was further authenticated by X-ray crystallography (see Table S1 and Figure S1).

A preheated flask was cooled to room temperature under argon. Subsequent to heating under vacuum refilling with argon thrice, the flask was charged with initiator, i.e., **BIB** or **TIB**, respectively, under an inert atmosphere. The predetermined amounts of monomer were added, i.e., EtOx, EtOx and MestOx or BocOx and EtOx, respectively. Subsequent to the addition of acetonitrile, the reaction mixture was heated to reflux using a heat-on system under a continuous gentle argon flow. Reactions times varied between 1.5 and 4 h, as optimized during the kinetic studies. Subsequently, the termination agents (1.3–2 equiv with respect to oxazolinium chain ends) were added under an inert atmosphere. The reaction mixtures were stirred at room temperature, 50 °C or overnight. Subsequent to taking an aliquot for analysis by means of ^1^H NMR spectroscopy and SEC, chloroform or dichloromethane was added. Excess amounts of termination agent and formed salts were removed by repeated washing steps with aqueous NaHCO_3_ solution, brine, and deionized water. The organic phase was dried by using Na_2_SO_4_, filtered, and concentrated under reduced pressure. Some cross-linkers were additionally precipitated from diethyl ether (−20 °C). All samples were dried in vacuo and analyzed by means of ^1^H and ^13^C NMR spectroscopy, SEC, and matrix-assisted laser desorption/ionization time-of-flight mass spectrometry (MALDI-TOF MS).

### 2.3 General Procedure for Deprotection of BocOx Containing Cross-Linkers

The BocOx containing cross-linkers were dissolved in dichloromethane, and trifluoroacetic acid (7–30 equiv per Boc protection group) was added. The reaction mixture was stirred at room temperature until gas evolution ceased (about 4 h). The solvent was evaporated under a reduced pressure. Purification procedures varied and are specified for each cross-linker in the Supporting Information. The amount of residual trifluoroacetate ions was determined by ^19^F NMR spectroscopy using potassium fluoride as an external standard. Further characterization methods included ^1^H and ^13^C NMR spectroscopy as well as MALDI-TOF MS. Data are provided in the Supporting Information.

### 2.4 General Procedure for Cryogel Preparation

For the cryogel preparations, 5 mL polypropylene syringes were used as reaction containers. For each condition, duplicates were carried out by preparing an 8 mL monomeric stock solution and distributing 3.9 mL into two 5 mL syringes each. An aqueous monomeric solution of 0.2 mol % acryloxyethyl thiocarbamoyl rhodamine B (RhoB), the corresponding cross-linker, and K_2_S_2_O_8_ was homogenized in an ultrasonic bath and was purged with argon at 0 °C for 30 min. The solution was taken up into a 5 mL syringe before addition of an aqueous *N*,*N*,*N*′,*N*′-tetramethylethylenediamine solution (80 μL of a 1.114 M solution, 0.089 mmol) through the bottom end. The syringe was bottom-capped using a syringe stopper and vortexed for 10 s. Cryo-polymerization was carried out by placing the capped syringe in a cryostat cooling bath (at −12 °C) overnight using a perforated polystyrene grid. Afterward, the syringe was removed from the cryostatic bath and thawed at room temperature for 1 h. The resulting cryogel was immersed in water to remove unreacted monomers with subsequent solvent changes for 5 days. Cryogel monoliths were cut into slices using a steel blade followed by lyophilization for at least 2 days. Slices were used for SEM imaging analysis, confocal laser scanning microscopy (CLSM), swelling experiments, and pH stability tests. Three or four slices were ground to a fine powder for analysis by means of ssNMR spectroscopy and thermogravimetric analysis (TGA). The exact experimental details are summarized in Tables S2 and S3 in the Supporting Information.

## 3 Results and Discussion

The goal of this work was the synthesis of two- and three-armed cross-linkers based on oligomeric 2-oxazolines (Scheme 1) for the preparation of cryogels. The development included the exploration of bi- and trifunctional CROP initiators as well as the design of suitable termination agents to introduce radically polymerizable ω-end groups in a direct fashion.

### 3.1 Kinetic Studies

CROP initiators comprising multiple initiation sites represented the basis for the development of the POx-based cross-linkers. Benzyl halides have long been known as suitable CROP initiators.^55,56^ Particularly, a range of benzyl bromides is commercially available. However, some of them tend to cause slow initiation, which can lead to multimodal molar mass distributions for multifunctional initiators.^57^ The use of iodine-based initiators can circumvent that problem, besides enhancing the polymerization rate compared to the corresponding bromine-based initiators.^55,58−60^ Therefore, two initiators based on iodomethylbenzene derivatives were synthesized via Finkelstein reactions in order to obtain symmetrical two- and three-armed cross-linkers (Scheme 1). The corresponding bi- and trifunctional initiators 1,4-bis(iodomethyl)benzene (**BIB**) and 1,3,5-tris(iodomethyl)benzene (**TIB**), respectively, were obtained according to the literature.^61,62^ However, **TIB** has not been used as an initiator for the CROP of 2-alkyl-2-oxazolines to the best of our knowledge.

To explore the feasibility of these compounds as CROP initiators, kinetic studies using EtOx as a monomer were conducted in acetonitrile at an initial monomer concentration [M]_0_ of 2 mol L^–1^ under reflux conditions to minimize chain transfer reactions during the CROP. The latter would result in the formation of proton-initiated species and, in consequence, would lead to impurities with single instead of multiple ω-end groups.^63^

In view of the applications as cross-linkers, the degree of polymerization per arm was set to five. This resulted in a total [M]_0_/[I]_0_ of 10 for the bifunctional initiator **BIB** and in a total [M]_0_/[I]_0_ of 15 for the trifunctional initiator **TIB**.

**Figure 1:**
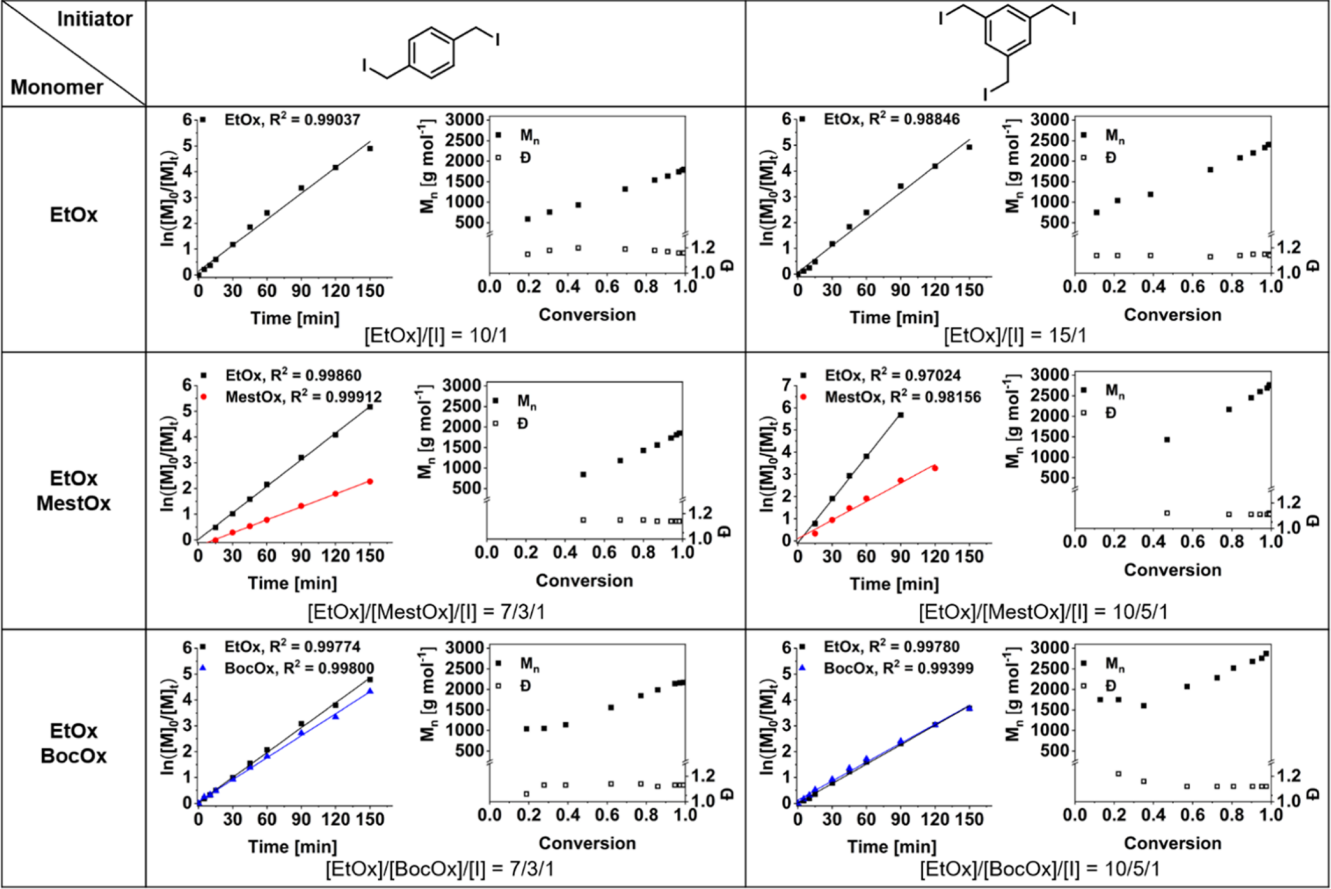
Kinetic plots for the CROP of EtOx, EtOx and MestOx as well as EtOx and BocOx initiated by **BIB** ([M]_0_/[I]_0_ = 10) and **TIB** ([M]_0_/[I]_0_ = 15) carried out in acetonitrile ([M]_0_ = 2 mol L^–1^, reflux). Apparent polymerization rate coefficients (*k*_p_) and polymerization rate coefficients per arm (*k*_p,arm_) were calculated from the slope of the corresponding linear fits in the first-order kinetic plots and are summarized in Table 1. Corresponding number-average molar masses (*M*_n_) and dispersity values (*Đ*) as plotted against conversion were determined by SEC.

The semi-logarithmic kinetic plots ln([M]_0_/[M]*_t_*) versus reaction time (Figure 1) increased in a linear fashion for both initiators. This is characteristic of a pseudo-first-order behavior with a rapid and efficient initiation. The different [M]_0_/[I]_0_ ratio resulted in apparent *k*_p_ values that deviated when comparing **BIB** and **TIB** (compare entries for **B-EtOx** and **T-EtOx**, respectively, in Table 1). However, [M]_0_/[I]_0_ per initiation site was kept constant as five. In consequence, the *k*_p,arm_ values were very similar, demonstrating an efficient initiation at each site for the bifunctional initiator **BIB** as well as for the trifunctional initiator **TIB**. The molar mass (*M*_n_) increased in a linear fashion when plotted against monomer conversion for both initiators, indicating that the molar mass can be well controlled. The monomodal molar mass distributions with narrow dispersity values (*Đ* < 1.2) confirmed the efficient and simultaneous initiation at all sites (Figures S2 and S3 in the Supporting Information).

We next investigated copolymerization reactions of EtOx with monomers featuring functional moieties. The methylester containing monomer MestOx and the Boc-protected primary amine containing BocOx were synthesized according to literature procedures.^50,52^ The fraction of functional monomer was set as ≈30% for both initiators. Therefore, the molar ratio of EtOx to functional comonomer was adjusted to 7:3 for bifunctional initiator **BIB**, whereas the ratio was 10:5 for trifunctional initiator **TIB**. Thereby, the targeted DP value of five per arm was maintained.

The copolymerization of EtOx with MestOx was of pseudo-first order, as indicated by the kinetic studies. The linear evolution of the molar mass with conversion was retained for both initiators, as were the monomodal molar mass distributions (*Đ* < 1.2, Figures S4 and S5 in the Supporting Information). The semi-logarithmic plots (Figure 1, second row) showed that the polymerization rates of EtOx and MestOx were very different from each other, which is well-known from the literature.^50^ As evident from the slope and the *k*_p,arm_ values (Table 1), EtOx was incorporated twice as fast as MestOx. Whereas these observations applied to both initiators, individual *k*_p,arm_ values differed from each other. The polymerization rate coefficient of EtOx was similar to that of the homopolymerization when using the bifunctional initiator **BIB** (compare entries for **B-EtOx** vs **B-MestOx** in Table 1). However, CROP using the trifunctional initiator **TIB** was accelerated by a factor of 2 when MestOx was used as a comonomer (compare entries for **T-EtOx** vs **T-MestOx** in Table 1). This acceleration of the propagation in case of using **TIB** could be related to the activation of the chain ends and/or stabilization of the transition state by the MestOx residues in the immediate vicinity as reported in the literature.^50,64^ As a result, potentially two MestOx units could stabilize the oxazolinium species. However, due to the symmetrical structure of **BIB**, these corresponding carbonyl groups are not in close vicinity, which may cause the difference.

In contrast, the use of BocOx as a functional comonomer did not significantly affect the propagation rate of EtOx (Table 1). The semi-logarithmic plot (Figure 1, third row) revealed a similar behavior of both monomers with almost overlapping linear regressions. The CROP tended to be slightly decelerated when the trifunctional initiator **TIB** was used. The *M*_n_ against conversion plot featured linear behavior as soon as conversions of >50% were reached. Deviations from linearity at lower conversions are due to overlapping monomer signals in the SEC elugrams (see Figures S6 and S7 in the Supporting Information).

### 3.2 Cross-Linker Synthesis by Termination of the CROP

As all kinetic studies hinted toward the presence of a living CROP, the next step included the termination of the oxazolinium chain ends with nucleophiles bearing radically polymerizable moieties. This direct termination approach avoided the need of further modification steps subsequent to polymerization within the cross-linker synthesis route. Acrylate, methacrylate, acrylamide as well as methacrylamide-based cross-linkers were designed to cover a range of common radically polymerizable moieties. PEtOx with acrylate or methacrylate ω-end groups were obtained by termination of the CROP with in situ deprotonated acrylic or methacrylic acid, respectively.^23−25^ Acrylamides and methacrylamides were introduced through piperazine derivatives as termination agents. For this purpose, *N*-acryloyl-piperazinium trifluoroacetate (**PipA**) and *N*-methacryloyl-piperazine (**PipMA**) were synthesized according to literature procedures.^65,66^ For the synthesis of **PipA**, *N*-Boc-piperazine was treated with acryloyl chloride to yield the Boc-protected amide. After deprotection by the addition of TFA and recrystallization in diethyl ether, the pure product was obtained in an overall yield of 75% and analyzed by ^1^H NMR spectroscopy and X-ray crystallography (see Table S1 and Figure S1 in the Supporting Information). **PipMA** was obtained as a yellow oil in rather low yields (25%) by the reaction of piperazine with methacrylic anhydride.^65^

The use of these four termination agents, the two initiators, and the monomer systems EtOx, EtOx/MestOx, and EtOx/BocOx resulted in 20 end-functionalized POx as listed in Table 2. The BocOx containing POx was subsequently deprotected to yield cross-linkers featuring amino moieties (AmOx). The cross-linkers were analyzed by means of SEC, ^1^H NMR spectroscopy as well as MALDI-TOF MS. The resulting characterization data are summarized in Table 3.

**Table 2:**
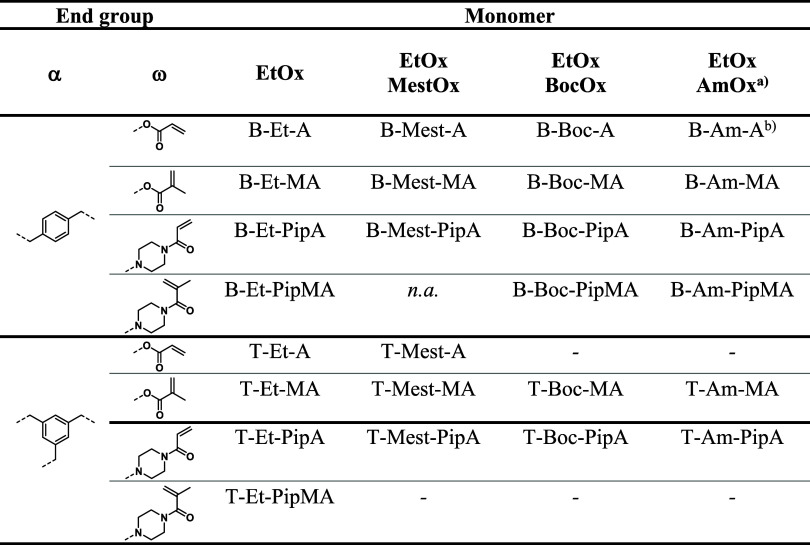
Overview on All Synthesized Cross-Linkers Based on **BIB** or **TIB** as Initiators, EtOx, and MestOx or BocOx as Monomers, Bearing Acrylate (**A**), Methacrylate (**MA**), and Piperazine-Based (**PipA** and **PipMA**) ω-End Groups

a Cross-linkers based on EtOx and AmOx were obtained after the deprotection of the BocOx containing analogues.

b Loss of the ω-end group was observed by ^1^H NMR spectroscopy.

**Table 3:** Summary of All Relevant Analytical Data of Synthesized Cross-Linkers Obtained from SEC (*M*_n_, *Đ*), MALDI-TOF MS (*M*_n_ and *Đ*), and^a^^1^H NMR (Comonomer Content in %)

	*M*_n_ [g mol^–1^]		*Đ*			
cross-linker	SEC^a^	MALDI	SEC^a^	MALDI	DF (NMR) [%]	comonomer content^f^ [%]
**B-Et-A**	2110	1280^b^	1.16	1.17^b^	94^d^	n.a.
**B-Et-MA**	1900	1460^b^	1.13	1.11^b^	92^d^	n.a.
**B-Et-PipA**	2270	1470^b^	1.13	1.09^b^	84^d^	n.a.
**B-Et-PipMA**	2300	1170^b^	1.09	1.10^b^	100^d^	n.a.
**T-Et-A**	2640	1850^b^	1.17	1.08^b^	97^d^	n.a.
**T-Et-MA**	2680	1950^b^	1.10	1.10^b^	97^d^	n.a.
**T-Et-PipA**	3020	1740^b^	1.17	1.16^b^	78^d^	n.a.
**T-Et-PipMA**	3200	2030^b^	1.06	1.06^b^	100^d^	n.a.
**B-Mest-A**	2010	1430^b^	1.17	1.11^b^	96^d^	27
**B-Mest-MA**	2030	1480^b^	1.18	1.10^b^	97^d^	28
**B-Mest-PipA**	2420	1650^b^	1.25	1.08^b^	83^d^	25
**T-Mest-A**	2930	1940^b^	1.20	1.05^b^	95^d^	35
**T-Mest-MA**	2920	2200^b^	1.12	1.08^b^	98^d^	34
**T-Mest-PipA**	3330	2300^b^	1.26	1.05^b^	89^d^	28
**B-Boc-A**	1630	1470^b^	1.19	1.13^b^	94^d^	29
**B-Boc-MA**	2070	1710^b^	1.19	1.09^b^	94^d^	29
**B-Boc-PipA**	2710	1810^b^	1.13	1.07^b^	84^d^	33
**B-Boc-PipMA**	2630	1850^b^	1.10	1.11^b^	100^d^	30
**T-Boc-MA**	3230	2220^b^	1.11	1.06^b^	98^d^	32
**T-Boc-PipA**	3795	2490^b^	1.21	1.07^b^	82^d^	35
**B-Am-A**	n.a.	1420^c^	n.a.	1.09^c^	14^e^	n.a.
**B-Am-MA**	n.a.	1520^c^	n.a.	1.06^c^	84^e^	31
**B-Am-PipA**	n.a.	1800^c^	n.a.	1.06^c^	82^e^	34
**B-Am-PipMA**	n.a.	1740^c^	n.a.	1.06^c^	94^e^	25
**T-Am-MA**	n.a.	1660^c^	n.a.	1.07^c^	86^e^	20
**T-Am-PipA**	n.a.	2320^c^	n.a.	1.04^c^	88^e^	35

a Values were determined using polystyrene (PS) as standard.

b Measurements were carried out using *trans*-2-[3-(4-*tert*-butylphenyl)-2-methyl-2-propenylidene] (DCTB) and sodium trifluoroacetate (NaTFA) as matrix mixture and poly(methyl methacrylate) (PMMA) for calibration.

c Measurements were carried out using α-cyano-4-hydroxycinnamic acid (CHCA) as matrix and poly(methyl methacrylate) (PMMA) for calibration.

d Measurements were carried out using CDCl_3_ as solvent.

e Measurements were carried out using D_2_O as solvent.

f Comonomer content was calculated from the corresponding monomer signals from ^1^H NMR spectroscopy.

All SEC elugrams revealed the presence of monomodal molar mass distributions indicating a uniform chain propagation at all initiating sites using both initiators (Figures S8–S27 the Supporting Information).

The presence of the desired ω-end groups was confirmed by ^1^H NMR spectroscopy. Vinylic proton signals were evident in the spectra of all cross-linkers (Figures S8–S27 in the Supporting Information). Integral values of these were used to estimate the degree of functionalization (DF) with the ω-end group by comparison with signal integrals derived from the benzylic methylene protons of the initiator. DF values were above 92% for the end groups attached via ester moieties, i.e., acrylate and methacrylate, which is in accordance with literature reports.^21−26^

**PipA** and **PipMA** have, to the best of our knowledge, not yet been used for end-capping of any CROP. In particular, **PipMA** proved to be well-suited for that purpose, as all DF were quantitative. For **PipA**, which represents a piperazinium salt with TFA, triethyl amine was added in situ to release the secondary amine as a nucleophile. DF values between 78 and 89% were reached. Variation of the excess of the termination agent or base did not increase the DF value further (data not shown). However, the nucleophilic attack of TFA on the living oxazolinium species was never observed.

In addition, the comonomer content was estimated from the ^1^H NMR spectra based on signals assigned to the methyl protons of the EtOx repeating units. For cross-linkers containing MestOx, the overlapping signals of the POx backbone and the MestOx methyl group were used. In contrast, the methyl proton signals of the Boc moiety were well separated, simplifying the calculations for materials containing BocOx. In all cases, overlapping end group signals were taken into account as appropriate for the individual cross-linkers according to the assignments in Figures S16–S27. The resulting comonomer content was in good agreement with the feed ratio used in all cases (Table 3).

**Figure 2:**
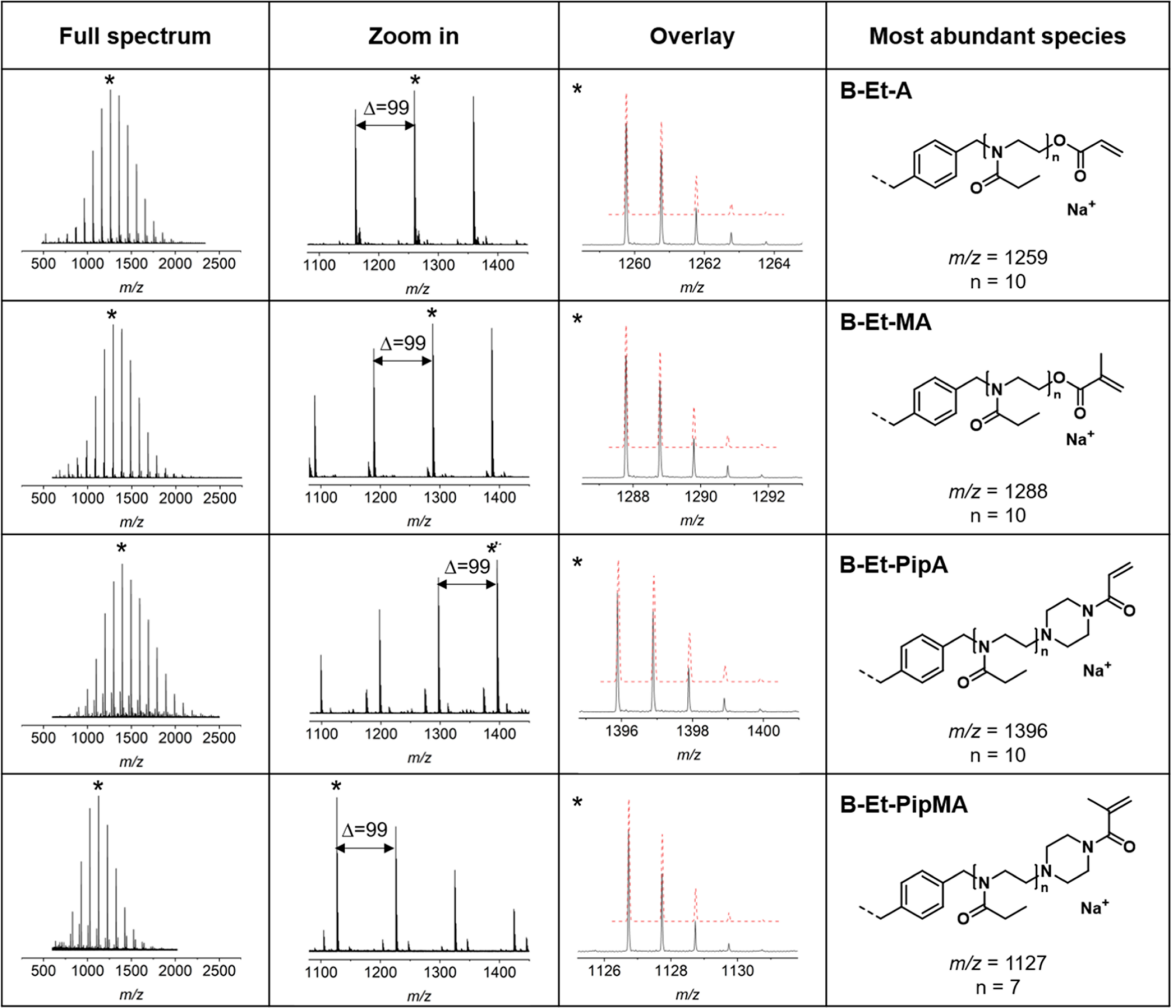
MALDI-TOF mass spectra (DCTB + NaTFA) of the different cross-linkers derived from the homopolymerization of EtOx using the bifunctional initiator **BIB**. From left to right: Full spectra, zoom into an *m*/*z* region displaying the EtOx repeating units, and overlay of the measured and calculated (red dotted line) isotopic pattern of the most abundant species (marked with *). All identified *m*/*z* species were found as sodium adducts.

The attachment of the various vinylic end groups was further confirmed by MALDI-TOF MS, as exemplified for cross-linkers derived from the homopolymerization of EtOx using the bifunctional initiator **BIB** in Figure 2. A repeating unit of 99 *m*/*z* was evident for all EtOx-based cross-linkers. The most abundant species were assigned to the desired structures, indicating a successful functionalization at all chain ends for all termination agents. Only the mass spectra of cross-linkers bearing **PipA** end groups featured a second pronounced *m*/*z* distribution, which is in accordance with the DF values described above. It was assigned to macromolecules comprising one **PipA** and one hydroxyl ω-end group. Analogous findings were made for the corresponding T-EtOx cross-linkers (see Figure S33 in the Supporting Information).

MALDI-TOF MS was also applied for the analysis of the cross-linkers containing MestOx or BocOx as comonomers (see Figures S34–S37 in the Supporting Information). For the MestOx-containing cross-linkers both repeating units for EtOx (Δ*m*/*z* = 99) and MestOx (Δ*m*/*z* = 157) were found confirming the successful synthesis of the copolymers. Accordingly, mass spectra of cross-linkers comprising BocOx revealed additional signals with a Δ*m*/*z* = 228, which corresponds to the molar mass of one BocOx repeating unit. The elevated molar mass of MestOx and BocOx repeating units resulted in increased *M*_n_ values for these cross-linkers compared to cross-linkers based on EtOx only (Table 3). For all cross-linkers, the end groups were confirmed by MS.

In summary, these characterization data show that well-defined POx can be obtained from bifunctional initiator **BIB** as well as trifunctional initiator **TIB**. Direct termination agents resulted in cross-linkers bearing radically polymerizable acrylate, methacrylate, acrylamide as well as methacrylamide-based end groups with high-end group fidelity.

### 3.3 Deprotection of BocOx Containing Cross-Linkers

**Figure 3:**
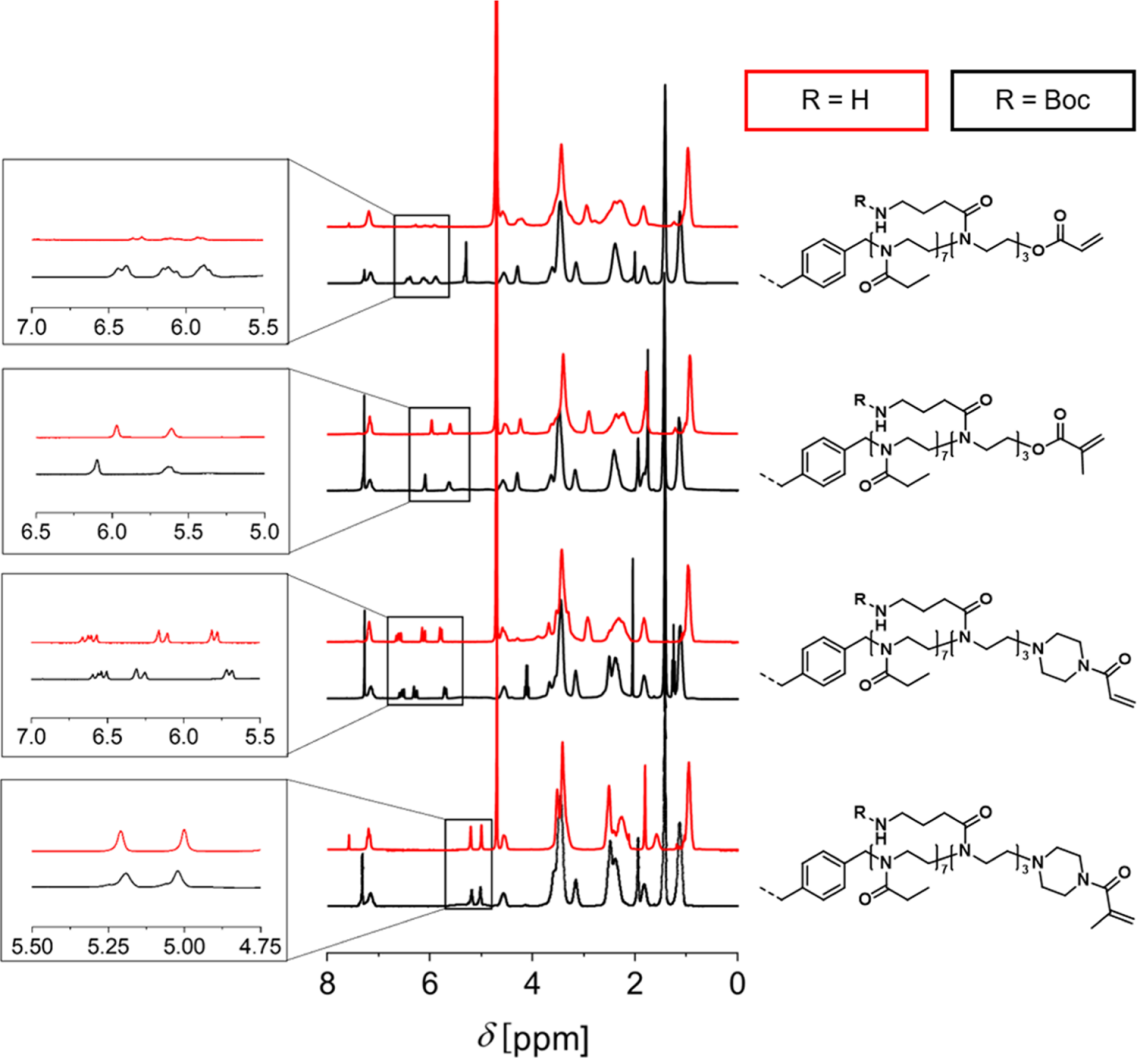
Middle: Overlay of ^1^H NMR spectra of the **B-BocOx** (black, CDCl_3_, 300 MHz) and the **B-AmOx** cross-linkers (red, D_2_O, 300 MHz). Left: Zoom into ^1^H NMR spectra of the double bond signals. Right: Schematic representation of the chemical structures of the **B-BocOx** and **B-AmOx** cross-linkers.

**Scheme 2:**
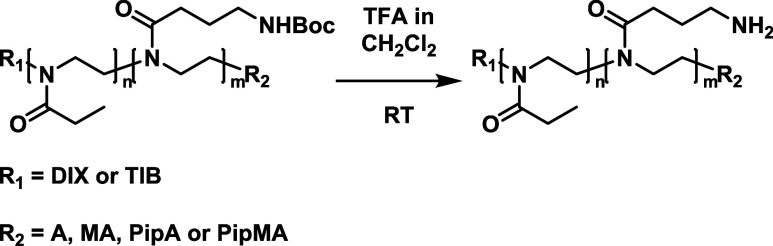
Schematic Representation of the Deprotection of the BocOx-Containing Cross-Linkers Using **BIB** or **TIB** as Initiators (R_1_) and Acrylate (**A**), Methacrylate (**MA**), **PipA**, or **PipMA** as the ω-End Group (R_2_)

A further functionalization of the cross-linkers via the amino moieties introduced by BocOx required the removal of the Boc protection groups. For this purpose, B-BocOx and T-BocOx oligomers were deprotected using trifluoroacetic acid (TFA) in order to obtain the corresponding B-Am and T-Am cross-linkers, respectively (Scheme 2). ^1^H NMR spectroscopy confirmed the successful removal of the Boc protecting group for all cross-linkers (Figure 3). An overview about the signal assignments in the ^1^H NMR spectra of the AmOx-based oligomers is given in the Supporting Information (see Figures S28–S32).

However, acrylate end groups were susceptible to cleavage from the cross-linkers during aqueous purification, both via extraction methods as well as by using anion exchange resins, as indicated by the loss of vinylic proton signals in the ^1^H NMR spectra. Despite their ester functionality, methacrylate end groups were more stable so that TFA removal via a chloride-modified anion exchange resin was successful. In addition to ^1^H NMR spectroscopy, MALDI-TOF MS clearly confirmed that these end groups were retained, although the DF was slightly lowered (Table 3 and Figures S38 and S39).

Cross-linkers featuring the more stable amide-based end groups **PipA** and **PipMA** could be deprotected without a decrease in DF. However, aqueous purification methods resulted in the Michael addition to cross-linkers containing **PipA** end groups, thereby impairing the polymerizable end group. This problem was avoided by purification through precipitation in diethyl ether to result in a cross-linker TFA salt. The amount of TFA in **B-Am-PipA** and **T-Am-PipA** determined from ^19^F NMR spectroscopy was in the expected range (5.0 or 8.5 TFA molecules per cross-linker molecule, respectively) (Figures S29 and S32). In contrast, the methacrylamide-based end group remained unaffected, even when aqueous purification methods were applied. However, the extraction using water and chloroform influenced the copolymer composition, as chains featuring a high amine content were susceptible to enrichment in the aqueous phase. **B-Am-PipMA** hence featured a lowered fraction of amino groups compared to that of the respective **B-Boc-PipMA** starting material. ^19^F NMR spectroscopy confirmed the removal of the excess of trifluoroacetic acid (Figures S28, S30, and S31).

### 3.4 Cryogel Preparation and Characterization

**Scheme 3:**
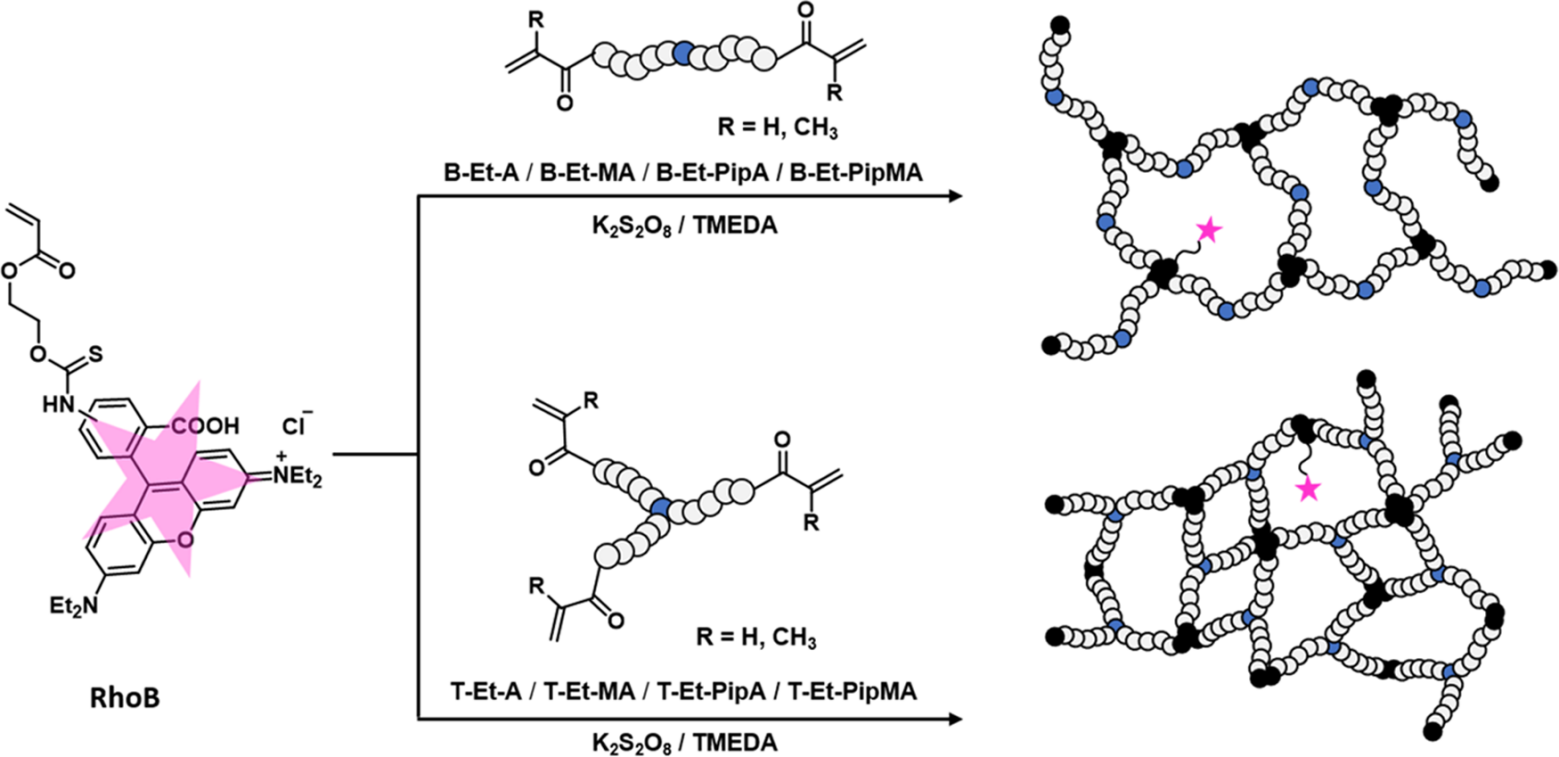
Schematic Representation of the Synthesis of Cryogels by Cryo-Polymerization at −12 °C Based on Bi- and Trifunctional EtOx-Based Cross-linkers Bearing Various ω-End Groups (**A**, **MA**, **PipA**, and **PipMA**) Acryloyloxyethyl thiocarbamoyl rhodamine B (**RhoB**) served as a comonomer to enable a potential observation of the cryogel pore structure by confocal laser scanning microscopy.

The two- and three-armed EtOx-based cross-linkers with either acrylate, methacrylate, or **PipA** as the ω-end group were successfully used in the preparation of cryogels which were obtained as stable monoliths. Acryloyloxyethyl thiocarbamoyl rhodamine B (**RhoB**) was used as a functional comonomer to enable visualization of the gel structure by CLSM (Scheme 3). An overview about the reaction conditions used for the gel preparation based on both **B-Et** and **T-Et** cross-linker series is provided in Tables S2 and S3 in the Supporting Information.

**Figure 4:**
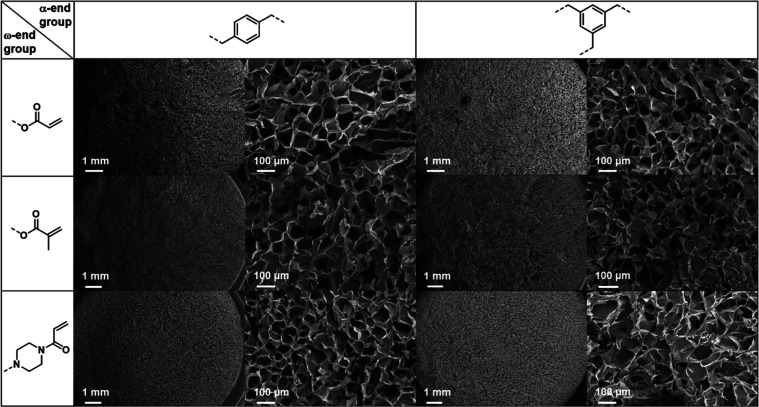
SEM micrographs of cryogels based on B-EtOx and T-EtOx cross-linkers with different ω-end groups. For each type, an entire picture of the gel slice as well as a zoomed view (121×) is displayed.

Only for **B-Et-PipMA** and **T-Et-PipMA**, no or a reduced gel formation was observed, which resulted in a large amount of remaining liquid feed solution after the reaction. The poor polymerizability of *N,N*-dialkyl methacrylamides was already described by Suzuki et al. and might be due to steric effects of the alkyl substituents.^67^ Scanning electron microscopy (SEM) imaging revealed sponge-like morphologies throughout the entire series with median pore sizes from 24 up to 39 μm (Figures 4 and S40). Under the chosen conditions, pore sizes could be reproduced for all two- and three-armed cross-linkers with three different ω-end groups. According to the statistical analysis, significant differences between the pore sizes of most cryogels were found (Figure S41 and Table S4). However, no correlation between the pore sizes and either the end group or the number of polymer arms in the cross-linkers was found.

**Figure 5:**
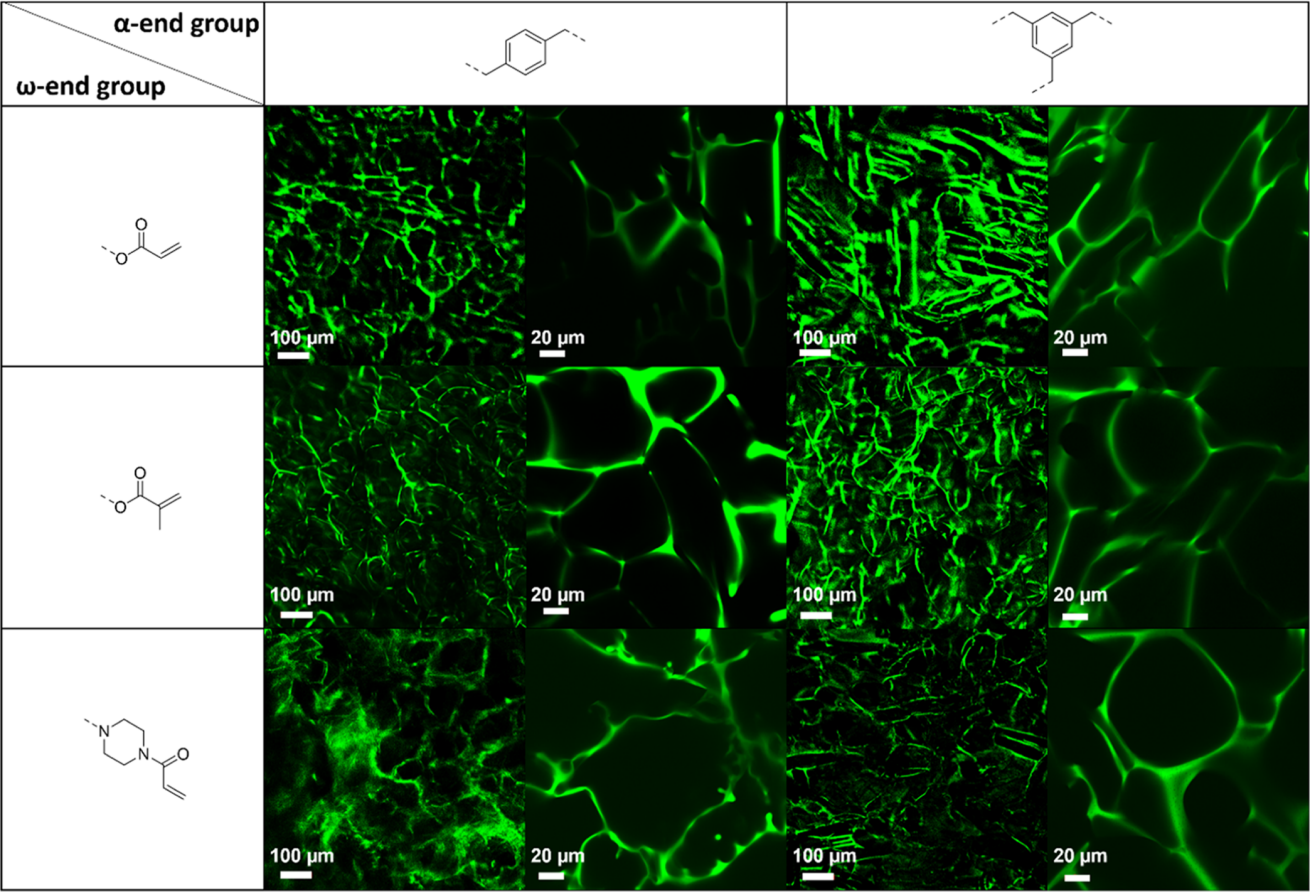
CLSM images of cryogels based on B-EtOx and T-EtOx cross-linkers with different ω-end groups (10 and 400× magnification, respectively).

The visualization of the cryogel pore structures in the swollen state was also possible by the use of CLSM due to incorporated Rhodamine B. The confocal images revealed a homogeneous distribution of the fluorescent dye throughout the entire polymer network (Figure 5). A rough estimation of the pore sizes was possible from the magnified images. Based on these manual measurements for each of the six cryogels compared to SEM, an increase of the pore sizes in the native, hydrated state was found, with pore sizes ranging from 32 μm up to 92 μm.

**Figure 6:**
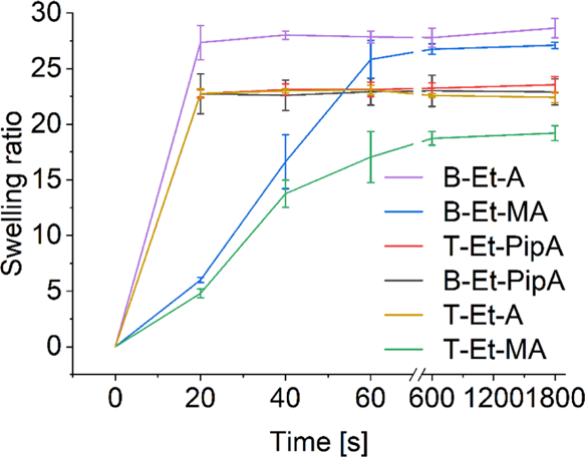
Swelling ratios of the cryogel series based on different POx cross-linkers. Swelling experiments were performed in triplicate with the exception of CG-T-Et-MA (*n* = 2).

Swelling experiments revealed significant differences in the water uptake ability among the cryogels (Figure 6 and Table S5). Cryogels based on trifunctional POx cross-linkers demonstrated a much slower swelling in comparison to their bifunctional analogues, except for the PipA containing polymers whose swelling ratios were almost identical. Presumably, this is due to the additional number of cross-linking points leading to an overall increasing cross-linking density/degree of cross-linking. Furthermore, acrylate and PipA containing cryogels reached the maximum swelling degree already after 20 s, whereas the methacrylate-containing analogues demonstrated a slower water uptake. This might be attributed to the additional methyl groups, which cause an increase in the hydrophobicity of the polymeric networks. However, all cryogels demonstrated a fast swelling behavior, which is comparable to PEGDA cryogels.^68^

In addition, no correlation between swelling and pore sizes for the individual cryogels was found.

^13^C ssNMR measurements revealed the presence of the different EtOx-based building blocks in the polymeric networks (see Figure S42 in the Supporting Information). No clear differences were visible within the spectra of cryogels based on either two- or three-armed macromonomers. In addition, all cryogels displayed excellent thermal stability up to at least 250 °C (see Figure S43 in the Supporting Information). Based on these results, the prepared cryogels are able to be sterilized by autoclaving (which usually proceeds at 120 °C) without material degradation prior to potential experiments with microorganisms or cells. When using **B-Et-A** and **B-Et-MA**, the first derivative TGA graphs displayed two major transition peaks at 355 and 415 °C or 304 and 417 °C, respectively. Notably, there was an intensity decrease of the earlier transition peak in the case of the **B-Et-MA**-based cryogels. In the case of **B-Et-PipA**-based cryogels, there is exclusively one transition peak at 428 °C, emphasizing the increased thermal stability of the piperazine-based ω-end group.

The use of three-armed instead of two-armed cross-linkers had no effect on the thermal stability of the cryogels, as shown by the almost identical curves. In addition, the stability of the prepared cryogels in aqueous solutions was investigated at different pH values (see Figure S44 in the Supporting Information). A complete dissolution of the cryogels containing acrylate ester bonds (**B-Et-A**, **T-Et-A**) was observed after 24 h under alkaline conditions. In contrast, methacrylate and **PipA** containing cryogel networks remained stable in a broad pH range even for 24 h.

## 4 Conclusions

We presented the development of cryogels that are solely based on cross-linkers derived from oligomeric 2-oxazolines. Thorough kinetic investigations of bifunctional and trifunctional benzyl iodides confirmed their excellent suitability as initiators for the CROP, confirming efficient and fast initiation at all sites. In addition to the successful incorporation of MestOx as well as BocOx as monomers comprising further functional moieties, the CROP could be terminated with four tailored nucleophiles, i.e., acrylate, methacrylate, piperazine-acrylamide, and piperazine-methacrylamide. The resulting two- and three-armed POx-based cross-linkers featured high-end group fidelity. The attachment of the radically polymerizable moieties through ester as well as amide bonds generated cross-linkers and cryogels with tunable hydrolytic stability. Whereas cryogels cross-linked via piperazine-acrylamide moieties were stable in a broad pH value and temperature range, POx-acrylate cross-linkers enabled access to materials that can be degraded under alkaline conditions.

Having developed access to the novel cryogels, our next steps include the exploitation of the broad range of materials that can now be made available. For instance, the varied cross-linking density will impact mechanical properties of the cryogels and could be further tuned by variation of the DP of the cross-linkers. In particular, cryogels consisting of MestOx and AmOx-based cross-linkers will be of interest for 3D cell culture as, e.g., peptides or sugars can be covalently attached to the materials.

## Supporting Information Available

Additional information concerning the synthesis procedures for the cross-linkers and the cryo-polymerization. X-ray data of **PipA**. SEC elugrams from the kinetic studies. ^1^H NMR spectra, SEC, and MALDI-TOF MS data from all cross-linkers. Cryogel pore size analysis based on automated SEM image analysis and swelling experiments. ssNMR spectra from all EtOx-based cross-linkers and the corresponding cryogels. TGA analysis and stability tests in pH = 0, 7, and 14 of all EtOx-based cryogels (PDF)

## Notes

Deposition number 2294139 contains the supplementary crystallographic data for this paper. These data are provided free of charge by the joint Cambridge Crystallographic Data Center and Fachinformationszentrum Karlsruhe, http://www.ccdc.cam.ac.uk.
