# Supplementary material for: Cryogels Based on Poly(2-oxazoline)s through Development of Bi- and Trifunctional Cross-Linkers Incorporating End Groups with Adjustable Stability
Source: Macromolecules. 2024 Mar 12;57(6):2915–27. doi: 10.1021/acs.macromol.3c02030 (PMC10977347; doi:10.1021/acs.macromol.3c02030)
Supplement: Supplementary file 1 — ma3c02030_si_001.pdf [file ma3c02030_si_001.pdf]

Supporting Information

Cryogels based on poly(2-oxazoline)s through development of bi- and trifunctional crosslinkers incorporating end groups with adjustable stability

Nora Engel[a,b,‡], Tim Hoffmann[a,b,‡], Florian Behrendt[a,b], Phil Liebing[c], Christine Weber[a,b], Michael Gottschaldt[a,b], Ulrich S. Schubert[a,b,*]

[a] Laboratory of Organic Chemistry and Macromolecular Chemistry (IOMC), Friedrich Schiller University Jena, Humboldtstr. 10, 07743 Jena, Germany

[b] Jena Center for Soft Matter (JCSM), Friedrich Schiller University Jena, Philosophenweg 7, 07743 Jena, Germany

[c] Laboratory of Inorganic and Analytical Chemistry (IAAC), Friedrich Schiller University Jena, Humboldtstr. 8, 07743 Jena, Germany

‡ Authors contributed equally to this work.

EXPERIMENTAL SECTION....4
1 Materials....4
2 Instruments....5
3 Initiator synthesis....9
3.1 1,4-*Bis*(iodomethyl)benzene (BIB)....9
3.2 1,3,5-*Tris*(iodomethyl)benzene (TIB)....9
4 Monomer synthesis....10
4.1 Methyl 4-((2-chloroethyl)amino)-4-oxobutanoate (MestOx-precursor)....10
4.2 Methyl 3-(4,5-dihydrooxazol-2-yl)propanoate (MestOx)....11
4.3 *tert*-Butyl (3-(4,5-dihydrooxazol-2-yl)propyl)carbamate (BocOx precursor)....12
4.4 2-[3-[(*tert*-Butyloxycarbonyl)amino]propyl]-4,5-dihydrooxazole (BocOx)....13
5 Termination agent synthesis....14
5.1 *tert*-Butyl 4-acryloylpiperazine-1-carboxylate (Precursor PipA)....14
5.2 *N*-Acryloyl-piperazinium trifluoroacetate (PipA)....15
5.3 *N*-Methacryloyl-piperazine (PipMA)....17
6 Kinetic studies....18
6.1 Homopolymerization of EtOx using BIB as initiator....18
6.2 Homopolymerization of EtOx using TIB as initiator....19
6.3 Copolymerization of EtOx and MestOx using BIB as initiator....19
6.4 Copolymerization of EtOx and MestOx using TIB as initiator....20
6.5 Copolymerization of EtOx and BocOx using BIB as initiator....20
6.6 Copolymerization of EtOx and BocOx using TIB as initiator....21
7 Crosslinker synthesis....22
7.1 Crosslinkers derived from the homopolymerization of EtOx....22

7.2 Crosslinkers derived from the copolymerization of EtOx and MestOx 32

7.3 Crosslinkers derived from the copolymerization of EtOx and BocOx 39

7.4 Deprotection of BocOx containing polymers yielding crosslinkers with amino moieties 46

8 Cryogel preparation 53

8.1 Bifunctional EtOx-based cryogels 53

8.2 Trifunctional EtOx-based cryogels 54

9 Additional Supporting Figures 55

9.1 MALDI-TOF mass spectra 55

9.2 Cryogel analysis 62

10 References 70

EXPERIMENTAL SECTION

1 MATERIALS

Barium oxide (BaO, 90%, ACROS), triethylamine (Sigma-Aldrich), acrylic acid stabilized with hydroquinone monomethylether (≥ 99%, Sigma-Aldrich), methacrylic acid stabilized with hydroquinone monomethylether (99%, Sigma-Aldrich), methacrylic anhydride containing 2,000 ppm topanol A as inhibitor (94%, Sigma-Aldrich), acryloyl chloride stabilized with 400 ppm phenothiazine (96%, Thermo Scientific), 1-(*tert*-butoxycarbonyl)piperazine (98%, Carbolution), trifluoroacetic acid (> 99%, TCI), *N*-(*tert*-butoxycarbonyl)-4-aminobutyric acid (> 98%, TCI), ethyl chloroformate (> 98% , Merck), 2-chlorethylamine hydrochloride (> 98%, TCI), sodium trifluoroacetate (NaTFA, 98%, Sigma-Aldrich), 1,3,5-*tris*(bromomethyl)benzene (> 98%, TCI), 1,4-*bis*(bromomethyl)benzene (> 98%, TCI), sodium iodide (> 99, TCI), piperazine (anhydrous, > 99.9%, TCI), potassium peroxodisulfate ($K_2S_2O_8$, ≥ 98%, Fluka Analytical), acryloxyethyl thiocarbamoyl rhodamine B (RhoB, ≤ 100%, Polysciences) and *N*,*N*,*N*‘,*N*‘-tetramethylethylenediamine (TMEDA, 99%, Sigma-Aldrich) were used as purchased. Prior to use, 2-ethyl-2-oxazoline (EtOx, ≥ 99%, Sigma-Aldrich) was stirred over BaO overnight, distilled to dryness and stored under argon atmosphere. Acetonitrile and *N*,*N*-dimethylformamide were obtained from a solvent purification system (SPS; Pure solv EN, InnovativeTechnology). Ambersep 900 (OH) (Thermo Fisher) was modified as follows: The anion exchanger was packed into a column and rinsed with brine in order to exchange the OH^- ions to Cl^- ions while continuous checking the pH value. After reaching a pH = 7, the excess of Cl^- ions deriving from brine was removed by rinsing the Ambersep 900 with deionized water while monitoring the process regularly using silver nitrate. All other chemicals and solvents were obtained from common commercial sources and used without further purification unless stated otherwise.

2 INSTRUMENTS

Proton and carbon nuclear magnetic resonance (^{1}H NMR, ^{13}C NMR) spectra were recorded on a 300 MHz spectrometer at room temperature equipped with an Avance I console, a dual proton (^{1}H), a carbon (^{13}C) sample head and a 120 × BACS automatic sample changer (Bruker, Germany). $CDCl_3$ or D_2O were used as NMR solvents. All chemical shifts are given in ppm and were determined using the residual non-deuterated solvent signal as reference. ^{13}C solid-state NMR spectra (^{13}C ssNMR) were recorded on a 400 MHz Bruker Avance III HD spectrometer at room temperature with a 4 mm MAS sample head.

Size exclusion chromatography (SEC) was performed on a Shimadzu system, equipped with a CBM-20A controller, a DGU-14A degasser, a LC-10AD vp pump, a Techlab oven (40 °C) and a RID 10A refractive index detector for data acquisition. As eluent, a mixture of chloroform/isopropanol/triethylamine (94/2/4; v/v/v) with a flow rate of 1 mL min^{-1} was applied. A PSS SDV guard/linear S (5 µm particle size) was used as column. Molar masses were estimated using polystyrene (PS) standards (162 g mol^{-1} to 128,000 g mol^{-1}).

Gas chromatography (GC) measurements were conducted on a Shimadzu GC-2010 plus using a system equipped with an AOC-20s auto sampler, AOC-20i injector and a flame ionization detector. Chloroform was used as a solvent. Helium was utilized as carrier gas and the separation was carried out on a Phenomenex ZB-5 column (5% phenyl, 95% dimethyl polysiloxane as stationary phase) with 30 m length, 0.25 mm inner diameter and 0.25 µm film thickness. GC was performed using the following set up: FID temperature 270 °C, split: 10%, injection volume: 0.2 µL; program: 2 min at 60 °C, heating with 16 K min^{-1} up to 200 °C, hold for 4 min at 200 °C (total: 14.75 min).

Matrix-assisted laser desorption ionization time-of-flight mass spectrometry (MALDI-TOF MS) measurements were carried out on a rapifleX MALDI TOF/TOF System (Bruker Daltonics) equipped with a scoutMTP II ion source and a smartbeam™ 3D laser (355 nm

wavelength). All spectra were measured in a positive reflector mode using α-cyano-4-hydroxycinnamic acid (CHCA) or *trans*-2-[3-(4-*tert*-butylphenyl)-2-methyl-2-propenylidene]malononitrile (DCTB) as matrix with sodium trifluoroacetate (NaTFA) as ionization additive. The recording was performed using manufacture's software flexControl 4.0. Evaluation and processing of the recorded spectra was done using manufacture's software flexAnalysis 4.0 and internal calibration using PMMA (2,500 Da) as a standard.

Electrospray ionization mass spectrometry (ESI MS) measurements were carried out on a Bruker MicroQTOF mass spectrometer. In addition, high-resolution (HR) ESI MS was performed using a Thermo QExactive plus Orbitrap mass spectrometer equipped with an ESI source using a calibration standard "ESI-L Low Concentration Tuning Mix" supplied from Agilent.

X-ray crystallography: The single-crystal X-ray intensity data for the compound **PipA** were collected on a Stoe IPDS 2T diffractometer equipped with a Cu-Kα fine-focus source and a 34 cm imaging-plate detector, at T = 130(2) K. The crystal structure was solved with SHELXT-2018/3[1] and refined by full matrix least-squares methods on F^2 with SHELXL-2018/3[2] using the Olex 1.2[3] environment. The crystallographic data are summarized in Table S1. CCDC 2294139 contains the supplementary crystallographic data for this publication. These data can be obtained free of charge from The Cambridge Crystallographic Data Centre (CCDC; http://www.ccdc.cam.ac.uk).

Scanning electron microscopy (SEM) imaging: Cryogel micrographs for morphological investigations and pore size measurements were obtained using a Zeiss Sigma VP Field Emission Scanning Electron Microscope (Jena, Germany) with an SE-2 detector and an accelerating voltage of 10 kV. For this purpose, freeze-dried cryogel slices were coated with a 9 nm platinum layer using a CCU-010 HV Safematic sputter coater (Zizers, Switzerland) and

fixed onto the plate holder pins with conductive carbon adhesive pads. A full view picture of the entire sample was obtained before taking selected views with higher magnifications.

Image analysis and pore size measurements were carried out based on the evaluation of SEM images. By the use of a deep learning-based algorithm established in JIPipe,[4-6] the cryogel pore sizes were automatically measured using four images per sample. Only the median values were considered as reliable measures to compare the pore size variation among the cryogel series. The establishment and creation of the algorithmic approach was described in an earlier work.[7] In brief, electron microscopy images were analyzed in TIFF 8-bit format, using a workflow written in JIPipe.[6] The analysis produced intensity-based and morphometric measures of the pores; the latter was also utilized to compare the automated framework of analysis with the manual measurements of the pores. The analysis was carried out by exploiting deep-learning (DL) methods. We applied the JIPipe implementation of the Cellpose framework after appropriate transfer learning.[8]

Statistical analysis. Cryogel pore sizes were compared using the Kruskal-Wallis analysis of variances (ANOVA) as well as Mood's median test. Statistical significance was set at $p < 0.05$. Differences between test groups were assessed in both methods using Dunns test.

Microscopic evaluation of hydrated cryogels was performed using the confocal laser scanning microscope LSM880 Elyra PS.1 system (Zeiss, Oberkochen, Germany) with a C-Apochromat 40×/1.2 W Korr FCS M27 objective. Images were acquired and analyzed using the ZEN software.

Thermogravimetric analysis (TGA) was carried out under nitrogen atmosphere using a Netzsch TG209 F1 Libra (Selb, Germany). For the measurements, a standard method was used as follows: Continuous heating from 25 °C to 600 °C with a heat rate of 20 K min^{-1}; time: 29 min). If necessary, the data were smoothed after the measurements using the Netzsch Proteus Thermal Analysis Software Version 8.0.2.

Lyophilization of polymers was conducted on an Alpha 2-4 LD plus freeze dryer from Martin Christ Gefriertrocknungsanlagen GmbH (Germany).

Ultrapure water was received from a Merck Millipore water purification system which was used for all cryogel preparations unless otherwise stated. For a precise and adjustable control of the reaction temperature for the cryopolymerization reactions, a FP40-MC cryostat from Julabo GmbH (Seelbach, Germany) was used.

3 INITIATOR SYNTHESIS

3.1 **1,4-*Bis*(iodomethyl)benzene (BIB)**

The synthesis was performed according to a literature procedure with slight modifications.[9] 1,4-*Bis*(bromomethyl)benzene (20.16 g, 0.08 mol, 1 eq.) and sodium iodide (45.80 g, 0.31 mol, 3.8 eq.) were suspended in distilled acetone (155 mL). The reaction mixture was stirred for 3 h at 60 °C. After the reaction mixture was allowed to reach room temperature, the solvent was removed under reduced pressure. The residue was suspended in chloroform (130 mL) and filtered. The organic layer was washed once with deionized water (100 mL), once with brine (100 mL), and dried over Na_2SO_4. The solvent was removed under reduced pressure and the crude product was recrystallized from 1,4-dioxane. The product was washed with cold diethyl ether and dried *in vacuo*. The product was obtained as yellow crystals. Yield: 12.6 g (44%).

^{1}H NMR ($CDCl_3$, 300 MHz): δ = 7.30 - 7.38 (m, 4H), 4.44 (s, 4H) ppm.

M_p: 173 to 174 °C.

3.2 1,3,5-*Tris*(iodomethyl)benzene (TIB)

The synthesis was performed according to a literature procedure with slight modifications.[10] 1,3,5-*Tris*(bromomethyl)benzene (20.20 g, 0.06 mol, 1 eq.) and sodium iodide (50.42 g, 0.34 mol, 6 eq.) were suspended in distilled acetone (150 mL). The reaction mixture was stirred for 3 h at 60 °C. Afterwards, the reaction mixture was cooled to room temperature and the formed NaBr was filtered off. The solvent was removed under reduced pressure and the residue was recrystallized from 1,4-dioxane. The product was washed with cold diethyl ether and dried *in vacuo*. The product was obtained as yellow crystals. Yield: 19.85 g (66%).

^{1}H NMR ($CDCl_3$, 300 MHz): δ = 7.27 (s, 3H,), 4.39 (s, 6H) ppm.

M_p: 122 to 123 °C.

4 MONOMER SYNTHESIS

4.1 Methyl 4-((2-chloroethyl)amino)-4-oxobutanoate (MestOx-precursor)

The synthesis was performed according to a literature procedure with slight modifications.[11] Methyl 4-chloro-4-oxobutyrate (50.00 g, 0.28 mol, 1 eq.) and 2-chloroethylamine hydrochloride (29.43 g, 0.31 mol, 1.1 eq.) were suspended in dry dichloromethane (400 mL) while cooling with an ice-bath. Triethylamine (81 mL, 0.59 mol, 2.1 eq.) was added to the reaction mixture within 1 h while keeping the reaction temperature between 0 °C and 5 °C. Afterwards, the reaction mixture was allowed to reach room temperature and stirred overnight. The reaction mixture was washed with deionized water (2 × 50 mL) and once with brine (50 mL). The organic layer was dried over Na_2SO_4, filtered, and the solvent was removed under reduced pressure. The product was purified by gel filtration column chromatography (neutral Al_2O_3, dichloromethane:methanol (99:1; v:v) and used without further purification. Yield: 54.37 g (88%).

^{1}H NMR ($CDCl_3$, 300 MHz): δ = 6.04 - 6.45 (m, 1H, NH), 3.68 (m, 7H), 2.62 - 2.75 (m, 2H), 2.46 - 2.58 (m, 2H) ppm.

4.2 Methyl 3-(4,5-dihydrooxazol-2-yl)propanoate (MestOx)

The synthesis was performed according to a literature procedure with slight modifications.[11] Methyl 4-((2-chloroethyl)amino)-4-oxobutanoate (54.37 g, 0.25 mol, 1 eq.) was mixed with anhydrous sodium carbonate (28.60 g, 0.27 mol, 1.1 eq.) and the reaction was performed for three days at the rotary evaporator (40 °C, 20 mbar) providing a continuous mixing of the viscous reaction mixture and a larger surface for the CO_2 release. Afterwards, distilled dichloromethane was added and the mixture was filtered. The solvent was removed under reduced pressure and the red-brown oil was stirred overnight over BaO. After distillation (115 °C, 0.02 mbar), the product was obtained as colorless liquid. Yield: 18.69 g (48%).

^{1}H NMR ($CDCl_3$, 300 MHz): δ = 4.12 - 4.34 (m, 2H), 3.74 - 3.89 (m, 2H), 3.68 (s, 3H), 2.52 - 2.72 (m, 4H) ppm.

4.3 *tert*-Butyl (3-(4,5-dihydrooxazol-2-yl)propyl)carbamate (BocOx precursor)

The synthesis was performed according to a literature procedure with slight modifications.[12] *N*-(*tert*-Butoxycarbonyl)-4-aminobutyric acid (25.0 g, 0.123 mol, 1 eq.) and triethylamine (33 mL, 0.246 mol, 2 eq.) were dissolved in tetrahydrofuran (400 mL) and cooled to 0 °C with an ice-bath. After the addition of ethyl chloroformate (13.07 mL, 0.135 mol, 1.1 eq.), the reaction mixture was allowed to reach room temperature and stirred for 1 h. A mixture of 2-chloroethylamine hydrochloride (15.07 g, 0.135 mol, 1.1 eq.), dimethylformamide (150 mL) and triethylamine (33 mL, 0.246 mol, 2 eq.) were added dropwise and the reaction mixture was stirred at room temperature overnight. The solvent was evaporated and the crude product was dissolved in dichloromethane, washed twice with sat. aq. $NaHCO_3$ solution, once with brine, and once with deionized water. The organic layer was dried over Na_2SO_4, filtered, and the solvent was removed under reduced pressure. The product was obtained as a yellowish solid. Yield: 19.44 g (60%).

^{1}H NMR ($CDCl_3$, 300 MHz): δ = 10.02 (br s, 2H, NH), 6.58 - 6.49 (m, 1H, CH), 6.38 - 6.32 (d, 1H, CH_2), 5.84 - 5.80 (d, 1H, CH_2), 3.61 - 3.50 (br s, 4H, 2×CH_2), 3.42 - 3.39 (t, 4H, 2×CH_2) ppm.

HR-ESI: *m/z* calculated for $C_{11}H_{21}N_2O_3Cl$ (M+Na$^+$): 287.1133; found: 287.1132 (error 0.2 ppm).

Elemental analysis calculated for $C_{11}H_{21}N_2O_3Cl$: C 49.9, H 8.00, N 10.58. Found: C 50.37, H 7.93, N 10.58.

4.4 2-[3-[(*tert*-Butyloxycarbonyl)amino]propyl]-4,5-dihydrooxazole (BocOx)

The synthesis was performed according to a literature procedure with slight modifications.[12] *tert*-Butyl (3-(4,5-dihydrooxazol-2-yl)propyl)carbamate (BocOx precursor) (18.33 g, 0.069 mol, 1 eq.) and potassium carbonate (19.21 g, 0.139 mol, 2 eq.) were suspended in dimethylformamide (150 mL). The reaction mixture was heated to 50 °C and stirred overnight. The solid was filtered off and the solvent was removed under reduced pressure. The yellowish solid was recrystallized from ethyl acetate. The product was obtained as colorless solid.

Yield: 10.48 g (66%).

^{1}H NMR ($CDCl_3$, 300 MHz): δ = 4.92 (br s, 1H, NH), 4.21 - 4.15 (t, 2H, CH_2), 3.80 - 3.74 (t, 2H, CH_2), 3.17 - 3.10 (m, 2H, CH_2), 2.30 - 2.25 (t, 2H, CH_2), 1.83 - 1.73 (m, 2H, CH_2), 1.39 (s, 9H, 3×CH_3) ppm.

HR-ESI: *m/z* calculated for $C_{11}H_{20}N_2O_3$ (M+Na$^+$): 229.1547; found: 229.1547 (error -0.2 ppm).

Elemental analysis calculated for $C_{11}H_{20}N_2O_3$: C 57.87, H 8.83, N 12.27. Found: C 57.70, H 8.77, N 12.17.

M_p: 89.6 °C.

5 TERMINATION AGENT SYNTHESIS

5.1 *tert*-Butyl 4-acryloylpiperazine-1-carboxylate (Precursor PipA)

The synthesis was performed according to a literature procedure with slight modifications.[13]

1-(*tert*-Butoxycarbonyl)piperazine (20.00 g, 0.107 mol, 1 eq.) and triethylamine (14.80 g, 0.107 mol, 1 eq.) were dissolved in dichloromethane (170 mL) and cooled to 0 °C with an ice-bath. Acryloyl chloride (9.43 mL, 0.117 mol, 1.1 eq) was dissolved in dichloromethane (80 mL) and added dropwise to the reaction mixture. The reaction mixture was allowed to reach room temperature and stirred for 3 h. The solution was washed three times with aq. sat. $NaHCO_3$ solution, two times with brine, and once with water. The organic layer was dried over Na_2SO_4, filtered, and the solvent was removed under reduced pressure. The product was obtained as a colorless solid. Yield: 25.15 g (97%).

^1H NMR ($CDCl_3$, 300 MHz): δ = 6.57 - 6.48 (m, 1H, CH), 6.29 - 6.23 (dd, 1H, CH_2), 5.70 - 5.66 (dd, 1H, CH_2), 3.61 - 3.50 (m, 4H, 2×CH_2), 3.42 - 3.39 (m, 4H, 2×CH_2) ppm.

HR-ESI: *m/z* calculated for $C_{12}H_{20}N_2O_3$ (M+Na^+): 263.1366; found: 263.1365 (error 0.6 ppm).

Elemental analysis calculated for $C_{12}H_{20}N_2O_3$: C 59.98, H 8.39, N 11.66. Found: C 59.93, H 8.34, N 11.67.

M_p: 77.8 °C.

5.2 *N*-Acryloyl-piperazinium trifluoroacetate (PipA)

The synthesis was performed according to a literature procedure with slight modifications.[13] *tert*-Butyl-4-acryloylpiperazine-1-carboxylate (20.00 g, 0.082 mol, 1 eq.) was dissolved in dichloromethane (160 mL) and trifluoroacetic acid (44 mL, 0.578 mol, 7 eq.) was added. The reaction mixture was stirred overnight at room temperature and the solvent was removed under reduced pressure. The crude product was dissolved in acetone (20 mL) and diethyl ether was added dropwise until the crystallization process started. After 4 h, the formed crystals were filtered off, washed with cold diethyl ether, and dried *in vacuo*. The product was obtained as a colorless solid. Yield: 16.02 g (77%).

^{1}H NMR ($CDCl_3$, 300 MHz): δ = 10.02 (br s, 2H, NH), 6.58 - 6.49 (m, 1H, CH), 6.38 - 6.32 (d, 1H, CH_2), 5.84 - 5.80 (d, 1H, CH_2), 3.61 - 3.50 (br s, 4H, 2× CH_2), 3.42 - 3.39 (t, 4H, 2× CH_2) ppm.

HR-ESI: *m/z* calculated for $C_7H_{12}N_2O$ (M+H$^+$): 141.1022; found: 141.1029 (error -4.7 ppm).

Table S1. Crystal data and details on structure refinement for **PipA**.

**CCDC deposition number**	2294139
**Molecular formula sum**	$C_9H_{13}F_3N_2O_3$
**Formula weight / g mol^{-1}**	254.21
**Crystal system**	monoclinic
**Space group**	$P2_1/c$
**Cell metric** ***a*** **/ Å**	8.436(1)
b **/ Å**	6.5421(6)
c **/ Å**	20.625(3)
**α / deg.**	90
**β / deg.**	101.46(1)
**γ / deg.**	90
**Cell volume / Å^3**	1115.5(2)
**Molecules per cell** ***z***	4
**Electrons per cell** F_{000}	528
**Calcd. density ρ / g cm^{-3}**	1.514

**μ / mm^{-1} (Cu-K$_\alpha$)**	1.281
**Crystal shape and color**	colorless plate
**Crystal size / mm**	0.26×0.12×0.03
**θ range / deg.**	4.375 … 66.569
**Reflections collected**	6022
**Reflections unique**	1966
**Reflections with I>2σ(I)**	1210
**Completeness of dataset**	99.5 %
**R_{int}**	0.0323
**Parameters; Restraints**	183; 27[a]
**R_1 (all data, I>2σ(I))**	0.1323; 0.0750
**wR_2 (all data, I>2σ(I))**	0.1955; 0.1512
**GooF (F^2)**	1.021
**Max. residual peaks**	–0.313; 0.438
**Extinction coefficient**	0.0020(6)

[a] Restraints on interatomic distances and anisotropic displacement parameters of the disordered Trifluoroacetate anion (SIMU, SADI).

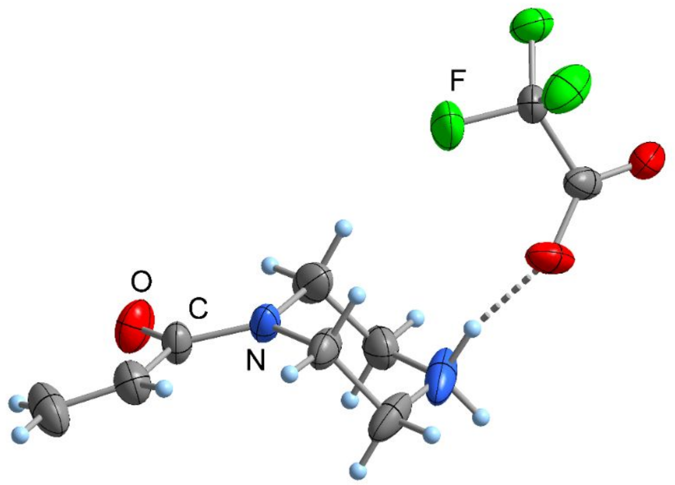

Figure S1. Molecular structure of PipA in the crystal. Displacement ellipsoids of the heavier atoms drawn at the 40% probability level, hydrogen atoms as spheres of arbitrary size. The minor-occupancy part of the disordered trifluoroacetate anion is omitted for clarity.

5.3 *N*-Methacryloyl-piperazine (PipMA)

The synthesis was performed according to a literature procedure with slight modifications.[14] Piperazine (21.62 g, 0.26 mol, 2 eq.) was dissolved in distilled dichloromethane (150 mL) and cooled to –10 °C with an NaCl/ice bath. Then, methacrylic anhydride (20 mL, 0.13 mol, 1 eq.) was dissolved in distilled dichloromethane (200 mL) and added dropwise to the reaction mixture over a period of 3.5 h keeping the temperature between –10 °C and –5 °C. The reaction mixture was allowed to reach room temperature and stirred for 1 h while monitoring the reaction progress by TLC (neutral Al_2O_3, dichloromethane:methanol (20:1; v:v), $R_f = 0.5$). The mixture was washed with 0.1 M aq. NaOH solution (200 mL). The aqueous layer was extracted with dichloromethane (3 × 200 mL). The combined organic layers were dried over Na_2SO_4, filtered, and the solvent was removed under reduced pressure. The product was purified by column chromatography (neutral Al_2O_3, dichloromethane:methanol (20:1; v:v), $R_f = 0.5$) and obtained as a colorless oil. Yield: 4.3 g (25%).

^{1}H NMR ($CDCl_3$, 300 MHz): δ = 5.17 (s, 1H), 5.02 (s, 1H), 3.56 (br s, 4H), 3.02 - 2.68 (m, 4H), 2.15 (s, 1H), 1.94 (s, 3H) ppm.

6 KINETIC STUDIES

All kinetic studies were performed according to the general procedure described in the main manuscript. Utilized amounts of chemicals for each kinetic study are detailed below.

6.1 Homopolymerization of EtOx using BIB as initiator

BIB (2.15 g, 6 mmol, 1 eq.), EtOx (6.06 mL, 60 mmol, 10 eq.) and 23.95 mL dry acetonitrile were used to result in an overall $[M]_0$ to $[I]_0$ ratio of 10 and an initial monomer concentration of 2 mol L^{-1}.

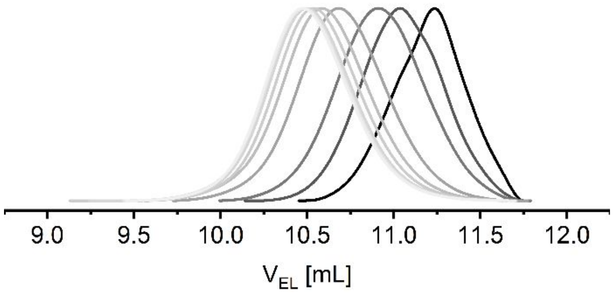

Figure S2. Overlay of the SEC elugrams (chloroform, triethylamine, *i*so-propanol, RI-detection) of the kinetic study of the polymerization of EtOx using BIB as initiator.

6.2 Homopolymerization of EtOx using TIB as initiator

TIB (3.29 g, 6.61 mmol, 1 eq.), EtOx (10.095 mL, 100 mmol, 15 eq.) and 39.91 mL dry acetonitrile were used to result in an overall $[M]_0$ to $[I]_0$ ratio of 15 and an initial monomer concentration of 2 mol L^{-1}.

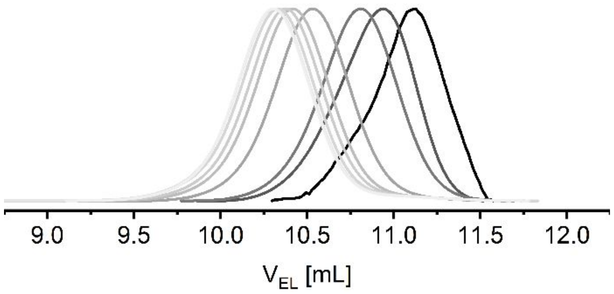

Figure S3. Overlay of the SEC elugrams (chloroform, triethylamine, *iso*-propanol, RI-detection) of the kinetic study of the polymerization of EtOx using TIB as initiator.

6.3 Copolymerization of EtOx and MestOx using BIB as initiator

BIB (2.90 g, 8.10 mmol, 1 eq.), EtOx (5.66 mL, 56 mmol, 7 eq.), MestOx (3.15mL, 24 mmol, 3 eq.) and 34.37 mL dry acetonitrile were used to result in an overall $[M]_0$ to $[I]_0$ ratio of 10, a ratio of [EtOx] to [MestOx] of 7 to 3, and an initial monomer concentration of 2 mol L^{-1}.

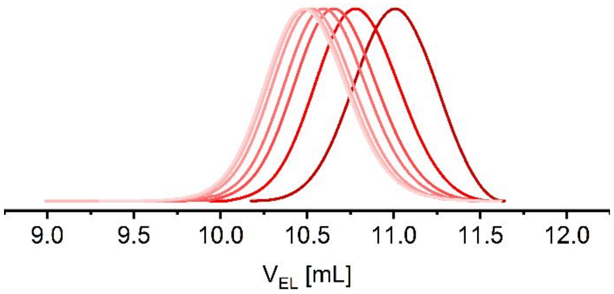

Figure S4. Overlay of the SEC elugrams (chloroform, triethylamine, *iso*-propanol, RI-detection) of the kinetic study of the copolymerization of EtOx and MestOx using BIB as initiator.

6.4 Copolymerization of EtOx and MestOx using TIB as initiator

TIB (1.99 g, 4 mmol, 1 eq.), EtOx (4.04 mL, 40 mmol, 10 eq.), MestOx (3.14 mL, 20 mmol, 5 eq.) and 22.82 mL dry acetonitrile were used to result in an overall $[M]_0$ to $[I]_0$ ratio of 15, a ratio of [EtOx] to [MestOx] of 10 to 5, and an initial monomer concentration of 2 mol L^{-1}.

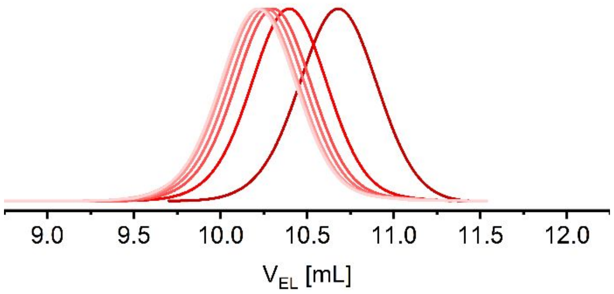

Figure S5. Overlay of the SEC elugrams (chloroform, triethylamine, *iso*-propanol, RI-detection) of the kinetic study of the copolymerization of EtOx and MestOx using TIB as initiator.

6.5 Copolymerization of EtOx and BocOx using BIB as initiator

BIB (1.00 g, 2.79 mmol, 1 eq.), EtOx (1.978 mL, 20 mmol, 7 eq.), BocOx (1.92 g, 8.41 mmol, 3 eq.) and 12 mL dry acetonitrile were used to result in an overall $[M]_0$ to $[I]_0$ ratio of 10, a ratio of [EtOx] to [BocOx] of 7 to 3, and an initial monomer concentration of 2 mol L^{-1}.

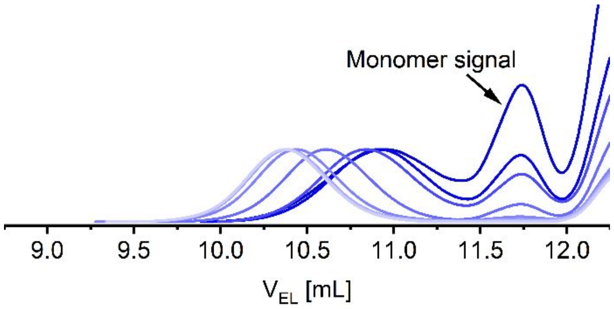

Figure S6. Overlay of the SEC elugrams (chloroform, triethylamine, *iso*-propanol, RI-detection) of the kinetic study of the copolymerization of EtOx and BocOx using BIB as initiator.

6.6 Copolymerization of EtOx and BocOx using TIB as initiator

TIB (1.00 g, 2 mmol, 1 eq.), EtOx (2.02 mL, 20 mmol, 10 eq.), BocOx (2.28 g, 10 mmol, 5 eq.) and 13 mL dry acetonitrile were used to result in an overall $[M]_0$ to $[I]_0$ ratio of 15, a ratio of [EtOx] to [BocOx] of 10 to 5, and an initial monomer concentration of 2 mol L^{-1}.

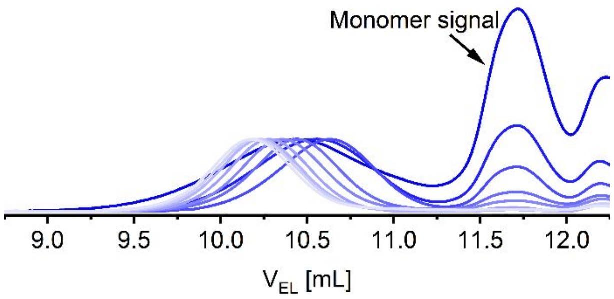

Figure S7. Overlay of the SEC elugrams (chloroform, triethylamine, *iso*-propanol, RI-detection) of the kinetic study of the copolymerization of EtOx and BocOx using TIB as initiator.

7 CROSSLINKER SYNTHESIS

All crosslinker syntheses were performed according to the general procedure described in the main manuscript.

7.1 Crosslinkers derived from the homopolymerization of EtOx

7.1.1 Bifunctional crosslinker with acrylate ω-end groups

B-Et-A. The polymerization was performed for 2.5 h according to the general procedure using BIB (2.15 g, 6 mmol, 1 eq.), EtOx (6.06 mL, 60 mmol, 10 eq.) and 23.95 mL dry acetonitrile The ω-chain ends were terminated by the addition of acrylic acid (1.65 mL, 24 mmol, 4 eq.) and triethylamine (3.20 mL, 24 mmol, 4 eq.) at a reaction temperature of 50 °C. Subsequent to purification by extraction, **B-Et-A** was re-dissolved in distilled dichloromethane and precipitated from cold diethyl ether. The polymer was obtained as a yellowish solid. Yield: 3.47 g (43%).

^{1}H NMR ($CDCl_3$, 300 MHz): 7.24 - 7.03 (m, 4H, CH**a**), 6.53 - 6.32 (m, 2H, CH, **f**), 6.28 - 5.99 (m, 2H, CH_2, **g**), 5.97 - 5.75 (m, 2H, CH_2, **g′**), 4.68 - 4.46 (m, 4H, CH_2, **b**), 4.29 (br s, 4H, CH_2, **c′**), 3.77 - 3.23 (m, 45H, CH_2, **c**), 2.59 - 2.12 (m, 25H, CH_2, **d**), 1.34 - 0.94 (m, 37H, CH_3) ppm; DP = 10; DF = 94%.

SEC (chloroform/*iso*-propanol/triethylamine [94/2/4; v/v/v], RI-detection, PS. cal.): $M_{n,SEC}$ = 2110 g mol^{-1}; Đ = 1.16.

MALDI-TOF MS (DCTB + NaTFA): $M_{n,MALDI}$ = 1280 g mol^{-1}; Đ = 1.17; [$C_8H_8(C_5H_9NO)_{10}(C_3H_3O_2)_2 + Na^+$] = 1259.759 *m/z* (calculated 1259.762 *m/z*) observed.

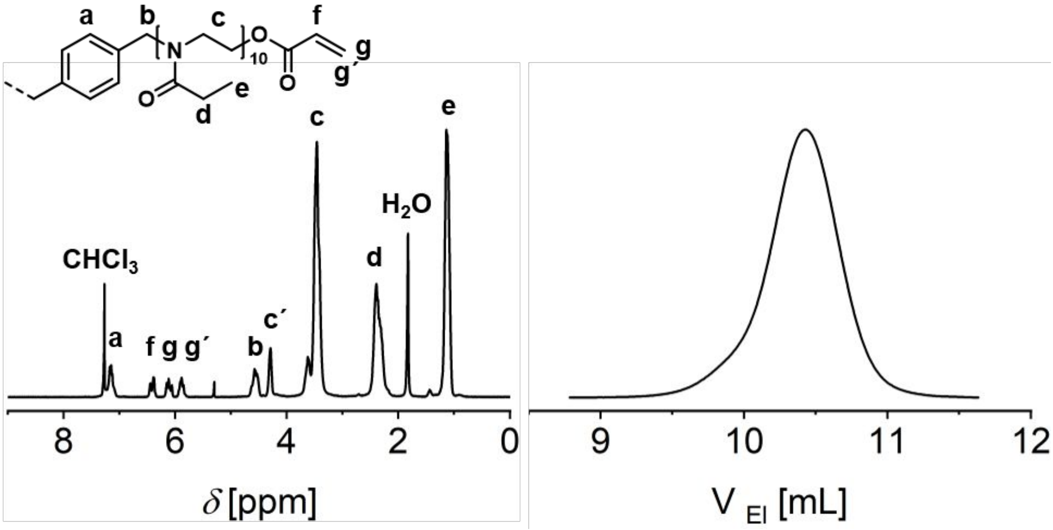

Figure S8. Characterization data of **B-Et-A**. Left: ^{1}H NMR spectrum (300 MHz, $CDCl_3$). Right: SEC elugram (chloroform, triethylamine, *i*so-propanol, RI-detection).

7.1.2 Bifunctional crosslinker with methacrylate ω-end groups

B-Et-MA. The polymerization was performed for 2.25 h according to the general procedure using BIB (4.31 g, 12 mmol, 1 eq.), EtOx (12.12 mL, 120 mmol, 10 eq.) and 47.9 mL dry acetonitrile. The ω-chain ends were terminated by the addition of methacrylic acid (4.05 mL, 48 mmol, 4 eq.) and triethylamine (6.45 mL, 48 mmol, 4 eq.) at a reaction temperature of 50 °C. The polymer was obtained as a yellowish solid. Yield: 8.54 g (72%).

^{1}H NMR ($CDCl_3$, 300 MHz): δ = 7.26 - 6.99 (m, 4H, CH **a**), 6.23 - 5.99 (m, 2H, CH_2 **g**), 5.74 - 5.52 (m, 2H, CH_2 **g´**), 4.79 - 4.45 (m, 4H, CH_2 **b**), 4.43 - 4.16 (m, 4H, CH_2 **c´**), 3.83 - 3.11 (m, 41H, CH_2 **c**), 2.67 - 2.11 (m, 23H, CH_2 **d**), 2.03 - 1.89 (m, 6H, CH_3 **f**), 1.53 - 1.00 (m, 35H, CH_3 **e**) ppm; DP = 11; DF = 92%.

SEC (chloroform/*iso*-propanol/triethylamine [94/2/4, v/v/v], RI-detection, PS cal.): $M_{n,\ SEC}$ = 1900 g mol^{-1}; Đ = 1.13.

MALDI-TOF MS (DCTB + NaTFA): $M_{n,MALDI}$ = 1460 g mol^{-1}, Đ = 1.11; [$C_8H_8(C_5H_9NO)_{11}(C_3H_3O_2)_3 + Na^+$] = 1386.866 *m/z* (calculated 1386.867 *m/z*).

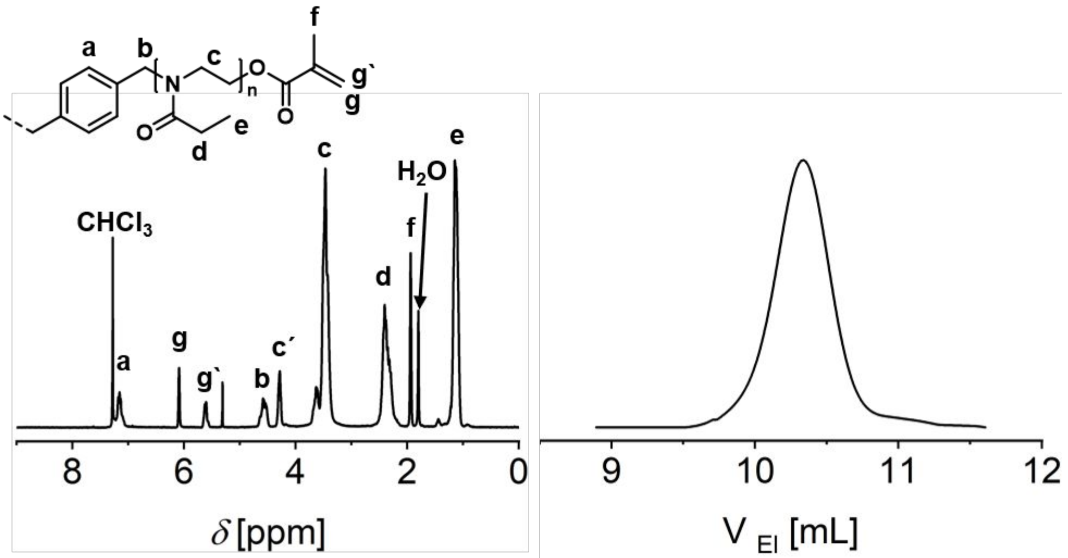

Figure S9. Characterization data of **B-Et-MA**. Left: ^1H NMR spectrum (300 MHz, $CDCl_3$). Right: SEC elugram (chloroform, triethylamine, *i*so-propanol, RI-detection).

7.1.3 Bifunctional crosslinker with *N*-acryloyl-piperazine (PipA) ω-end groups

B-Et-PipA. The polymerization was performed for 1.5 h according to the general procedure using BIB (2.146 g, 6 mmol, 1 eq.), EtOx (6.06 mL, 60 mmol, 10 eq.) and 14.00 mL dry acetonitrile. The ω-chain ends were terminated by the addition of PipA (3.972 g, 15.6 mmol, 2.6 eq.) and triethylamine (2.41 mL, 18 mmol, 3 eq.) at room temperature. The polymer was obtained as a yellowish solid Yield: 5.04 g (60%).

^1H NMR ($CDCl_3$, 300 MHz): δ = 7.19 - 7.10 (m, 4H, CH **a**), 6.61 - 6.51 (m, 2H, CH **h**), 6.32 - 6.27 (br, 2H, CH_2 **i**), 5.73 - 5.69 (br, 2H, CH_2 **i′**), 4.59 - 4.55 (m, 4H, CH_2 **b**), 3.69 - 3.46 (br, 46H, CH_2 **c**, CH_2 **g**), 2.52 - 3.34 (br m, 33H, CH_2 **d**, CH_2 **f**), 1.12 (br, 32H, CH_3 **e**) ppm; DP = 10; DF = 84%.

SEC (chloroform/*iso*-propanol/triethylamine [94/2/4, v/v/v], RI-detection, PS cal.): $M_{n,\,SEC}$ = 2270 g mol^{-1}; Đ = 1.13.

MALDI-TOF MS (DCTB + NaTFA): $M_{n,MALDI}$ = 1470 g mol^{-1}; Đ = 1.09; [$C_8H_8(C_5H_9NO)_{10}(C_7H_{11}N_2O)_2 + Na^+$] = 1395.883 *m/z* (calculated 1395.910 *m/z*).

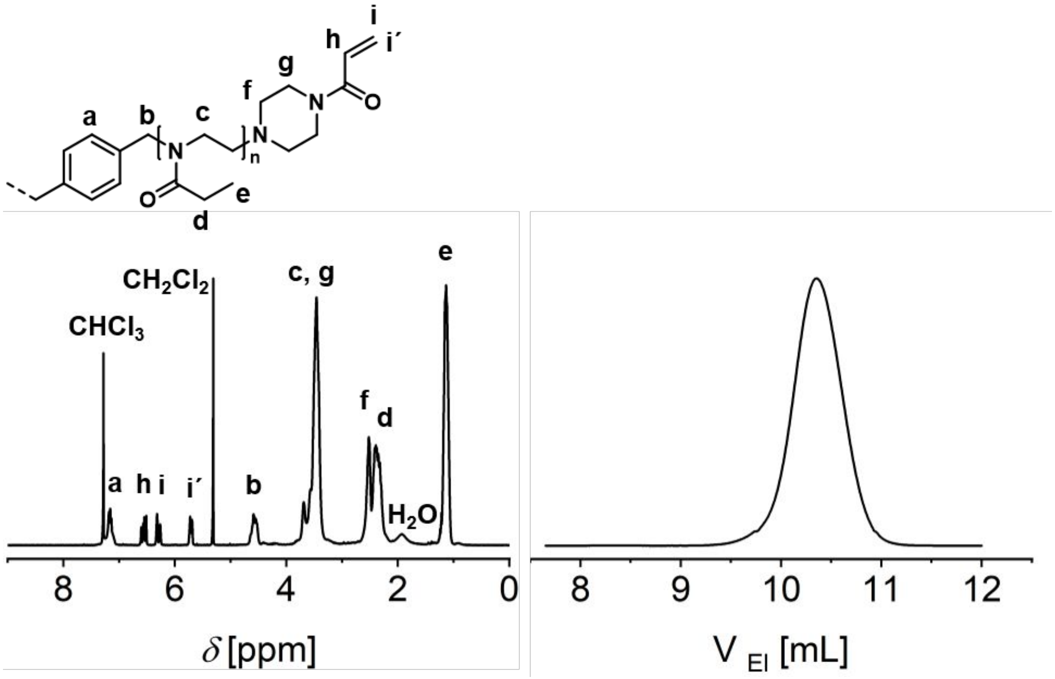

Figure S10. Characterization data of **B-Et-PipA**. Left: ^{1}H NMR spectrum (300 MHz, $CDCl_3$). Right: SEC elugram (chloroform, triethylamine, *iso*-propanol, RI-detection).

7.1.4 Bifunctional crosslinker with *N*-methacryloyl-piperazine (PipMA) ω-end groups

B-Et-PipMA. The polymerization was performed for 1.75 h according to the general procedure using BIB (10.09 g, 30 mmol, 1 eq.), EtOx (30.28 mL, 300 mmol, 10 eq.), and 66 mL dry acetonitrile. The ω-chain ends were terminated by the addition of PipMA (13.5 mL, 90 mmol, 3 eq.) and triethylamine (16.0 mL, 120 mmol, 4 eq.) at a reaction temperature of 50 °C. The polymer was obtained as a yellowish solid. Yield: 39.10 g (92%). ^{1}H NMR ($CDCl_3$, 300 MHz): δ = 7.12 (br, 4H, CH **a**), 5.15 (br, 2H, CH_2 **i**), 4.98 (br, 2H, CH_2 **i´**), 4.54 (br, 4H, CH_2 **b**), 3.55 - 3.42 (br, 45H, CH_2 **c**, CH_2 **g**), 2.44 - 2.38 (br m, 35H, CH_2 **d**, CH_2 **f**), 1.91 (br, 6H, CH_3 **h**), 1.09 (br, 31H, CH_3 **e**) ppm; DP = 10; DF = quant..

SEC (chloroform/*iso*-propanol/triethylamine [94/2/4, v/v/v], RI-detection, PS cal.): $M_{n,\,SEC}$ = 2300 g mol^{-1}; Đ = 1.09.

MALDI-TOF MS (DCTB + NaTFA): $M_{n,MALDI}$ = 1170 g mol^{-1}; Đ = 1.10; $[C_8H_8(C_5H_9NO)_7(C_8H_{13}N_2O)_2 + Na^+]$ = 1126.725 *m/z* (calculated 1126.736 *m/z*).

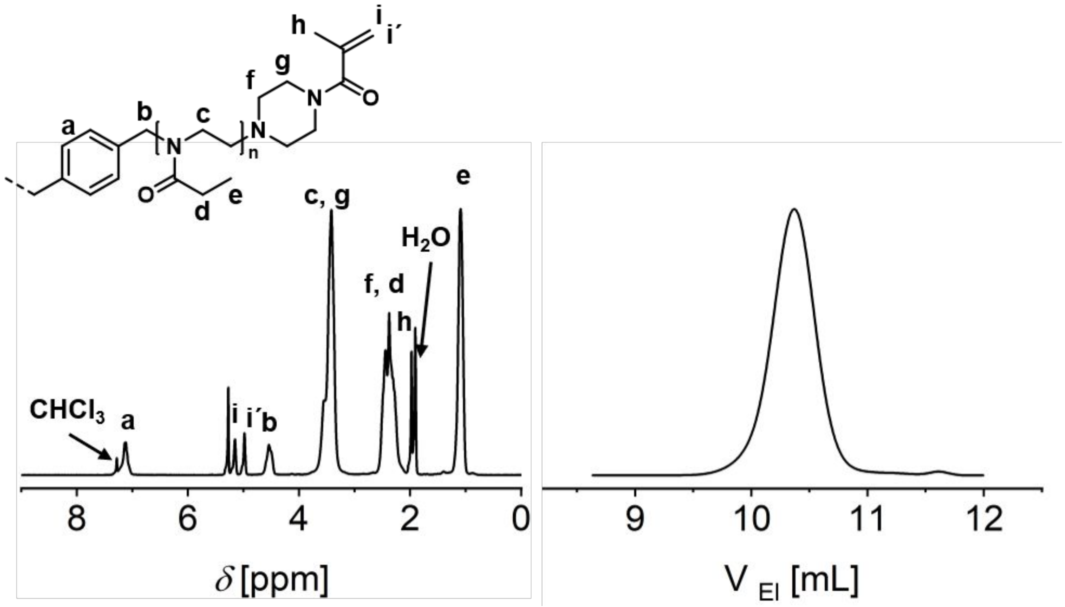

Figure S11. Characterization data of **B-Et-PipMA**. Left: ^{1}H NMR spectrum (300 MHz, $CDCl_3$). Right: SEC elugram (chloroform, triethylamine, *i*so-propanol, RI-detection).

7.1.5 Trifunctional crosslinker with acrylate ω-end groups

T-Et-A. The polymerization was performed for 2.5 h according to the general procedure using TIB (3.39 g, 6.67 mmol, 1 eq.), EtOx (10.1 mL, 100 mmol, 15 eq.) and 39.9 mL dry acetonitrile. The ω-chain ends were terminated by the addition of acrylic acid (2.75 mL, 40 mmol, 6 eq.) and triethylamine (5.4 mL, 40 mmol, 6 eq.) at a reaction temperature of 50 °C. The polymer was obtained as a yellowish solid. Yield: 8.74 g (88%).

^{1}H NMR ($CDCl_3$, 300 MHz): δ = 7.13 - 6.71 (m, 3H, CH **a**), 6.54 - 6.30 (m, 3H, CH **f**), 6.25 - 5.99 (m, 3H, CH_2 **g**), 5.95 - 5.72 (m, 3H, CH_2 **g´**), 4.55 (br s, 6H, CH_2 **b**), 4.39 - 4.12 (m, 6H,

CH_2 **c′**), 3.84 - 3.01 (m, 57H, CH_2 **c**), 2.60 - 1.87 (m, 34H, CH_2 **d**), 1.12 (br d, 48H, CH_3 **e**) ppm; DP = 14; DF = 97%.

SEC (chloroform/*iso*-propanol/triethylamine [94/2/4, v/v/v], RI-detection, PS cal.): $M_{n,SEC}$ = 2640 g mol^{-1}; Đ = 1.17.

MALDI-TOF MS (DCTB + NaTFA): $M_{n,MALDI}$ = 1850 g mol^{-1}; Đ = 1.08; $[C_9H_9(C_5H_9NO)_{15}(C_3H_3O_2)_3 + Na^+]$ = 1839.130 *m/z* (calculated 1839.126 *m/z*).

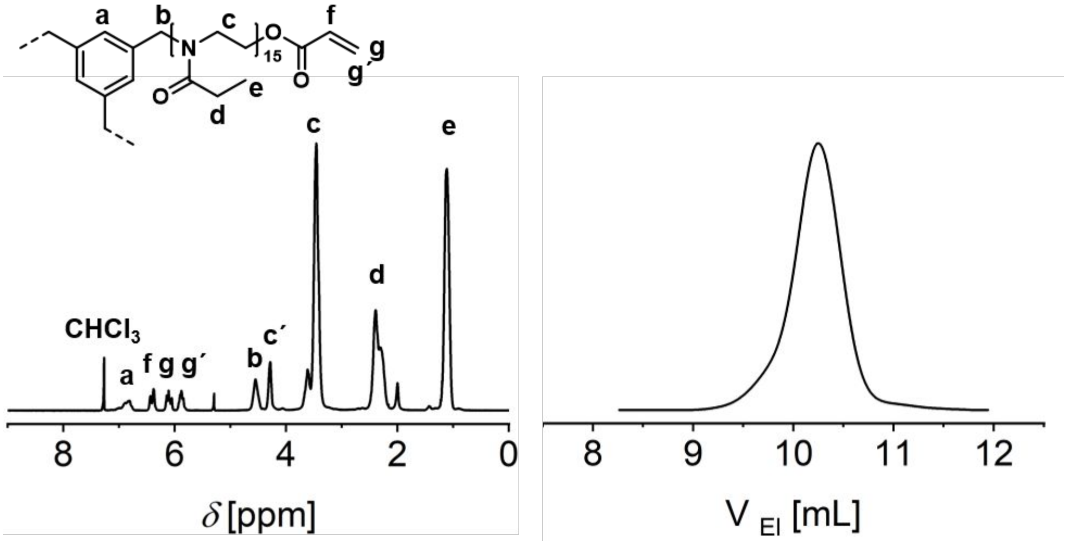

Figure S12. Characterization data of **T-Et-A**. Left: ^{1}H NMR spectrum (300 MHz, $CDCl_3$). Right: SEC elugram (chloroform, triethylamine, *i*so-propanol, RI-detection).

7.1.6 Trifunctional crosslinker with methacrylate ω-end groups

T-Et-MA. The polymerization was performed for 2.5 h according to the general procedure using TIB (9.2 g, 18 mmol, 1 eq.), EtOx (28 mL, 280 mmol, 15 eq.) and 112 mL dry acetonitrile. The ω-chain ends were terminated by the addition of methacrylic acid (9.45 mL, 112 mmol, 6 eq.) and triethylamine (15 mL, 112 mmol, 6 eq.) at a reaction temperature of 50 °C. The polymer was obtained as a yellowish solid. Yield: 25.62 g (90%).

^{1}H NMR ($CDCl_3$, 300 MHz): δ = 7.15 - 6.65 (m, 3H, CH **a**), 6.08 (br s, 3H, CH_2 **g**), 5.74 - 5.51 (m, 3H, CH_2 **g´**), 4.55 (br s, 6H, CH_2 **b**), 4.28 (br s, 6H, CH_2 **c´**), 3.89 - 3.06 (m, 55H, CH_2 **c**), 2.69 - 2.08 (m, 31H, CH_2 **d**), 1.93 (br s, 8H, CH_3 **f**), 1.13 (br s, 45H, CH_3 **e**) ppm; DP = 15; DF = 97%.

SEC (chloroform/*iso*-propanol/triethylamine [94/2/4, v/v/v], RI-detection, PS cal.): $M_{n,SEC}$ = 2680 g mol^{-1}; Đ = 1.10.

MALDI-TOF MS (DCTB + NaTFA): $M_{n,MALDI}$ = 1950 g mol^{-1}; Đ = 1.10; $[C_9H_9(C_5H_9NO)_{15}(C_4H_5O_2)_3 + Na^+]$ = 1881.157 *m/z* (calculated 1881.173 *m/z*).

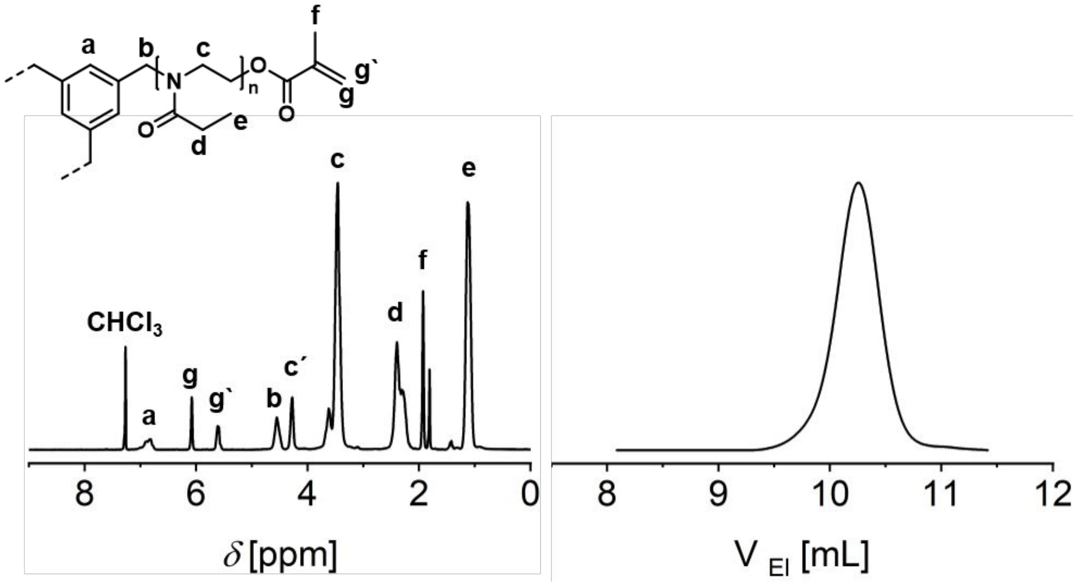

Figure S13. Characterization data of **T-Et-MA**. Left: ^{1}H NMR spectrum (300 MHz, $CDCl_3$). Right: SEC elugram (chloroform, triethylamine, *i*so-propanol, RI-detection).

7.1.7 Trifunctional crosslinker with *N*-acryloyl-piperazine (PipA) ω-end groups

T-Et-PipA. The polymerization was performed for 1.5 h according to the general procedure using TIB (2.49 g, 5 mmol, 1 eq.), EtOx (7.57 mL, 75 mmol, 10 eq.) and 17.42 mL dry acetonitrile. The ω-chain ends were terminated by the addition of PipA (4.91 g, 19.5 mmol, 3.9 eq.) and triethylamine (3.01 mL, 22.5 mmol, 4.5 eq.) at room temperature. **T-Et-PipA** was dissolved in dichloromethane, and precipitated in cold diethyl ether. The polymer was dried *in vacuo* and obtained as a yellowish solid. Yield: 6.42 g (64%).

^{1}H NMR ($CDCl_3$, 300 MHz): δ = 7.00 - 6.80 (br, 3H, CH **a**), 6.60 - 6.50 (m, 3H, CH **h**), 6.31 - 6.25 (br, 2H, CH_2 **i**), 5.72 - 5.69 (br, 2H, CH_2 **i′**), 4.57 (br, 7H, CH_2 **b**), 3.68 - 3.46 (br, 62H, CH_2 **c**, CH_2 **g**), 2.52 - 3.31 (br m, 50H, CH_2 **d**, CH_2 **f**) 1.12 (br, 44H, CH_3 **e**) ppm; DP = 15; DF = 78%.

SEC (chloroform/*iso*-propanol/triethylamine [94/2/4, v/v/v], RI-detection, PS cal.): $M_{n,SEC}$ = 2960 g mol^{-1}; Đ = 1.17.

MALDI-TOF MS (DCTB + NaTFA): $M_{n,MALDI}$ = 1740 g mol^{-1}; Đ = 1.16; [$C_9H_9(C_5H_9NO)_{15}(C_7H_{11}N_2O)_3$ + Na^+] = 2043.321 *m/z* (calculated 2043.347 *m/z*).

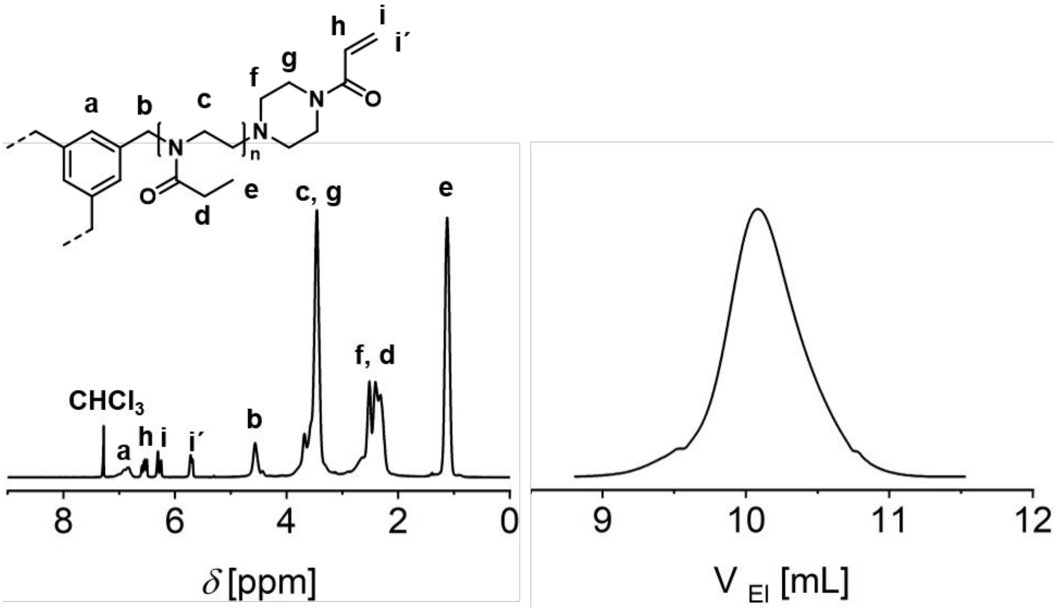

Figure S14. Characterization data of **T-Et-PipA**. Left: ^{1}H NMR spectrum (300 MHz, $CDCl_3$). Right: SEC elugram (chloroform, triethylamine, *iso*-propanol, RI-detection).

7.1.8 Trifunctional crosslinker with *N*-methacryloyl-piperazine (PipMA) ω-end groups

T-Et-PipMA. The polymerization was performed for 2.4 h according to the general procedure using TIB (1.33 g, 2.67 mmol, 1 eq.), EtOx (4.04 mL, 40 mmol, 15 eq.) and 15.96 mL dry acetonitrile. The ω-chain ends were terminated by the addition of PipMA (1.9 g, 12 mmol, 4.5 eq.) dissolved in 1 mL dry acetonitrile and 1.8 mL (13 mmol, 5 eq.) triethylamine at a reaction temperature of 50 °C. The polymer was obtained as a colorless solid. Yield: 4.02 g (100%).

^{1}H NMR ($CDCl_3$, 300 MHz): δ = 7.07 - 6.66 (m, 3H, CH **a**), 5.21 (br s, 3H, CH_2 **i**), 5.04 (br s, 3H, CH_2 **i´**), 4.57 (br d, 5H, CH_2 **b**), 3.84 - 3.12 (m, 63H, CH_2 **c**, CH_2 **g**), 2.67 - 2.14 (m, 46H, CH_2 **d**, CH_2 **f**), 1.96 (br s, 9H, CH_3 **h**),1.14 (br s, 43H, CH_3 **e**) ppm; DP = 16; DF = quant.

SEC (chloroform/*iso*-propanol/triethylamine [94/2/4, v/v/v], RI-detection, PS cal.): $M_{n,SEC}$ = 3200 g mol^{-1}; Đ = 1.06.

MALDI-TOF MS (DCTB + NaTFA): $M_{n,MALDI}$ = 2030 g mol^{-1}; Đ = 1.06; [$C_9H_9(C_5H_9NO)_{14}(C_8H_{13}N_2O)_3 + Na^+$] = 1986.323 *m/z* (calculated 1986.329 *m/z*).

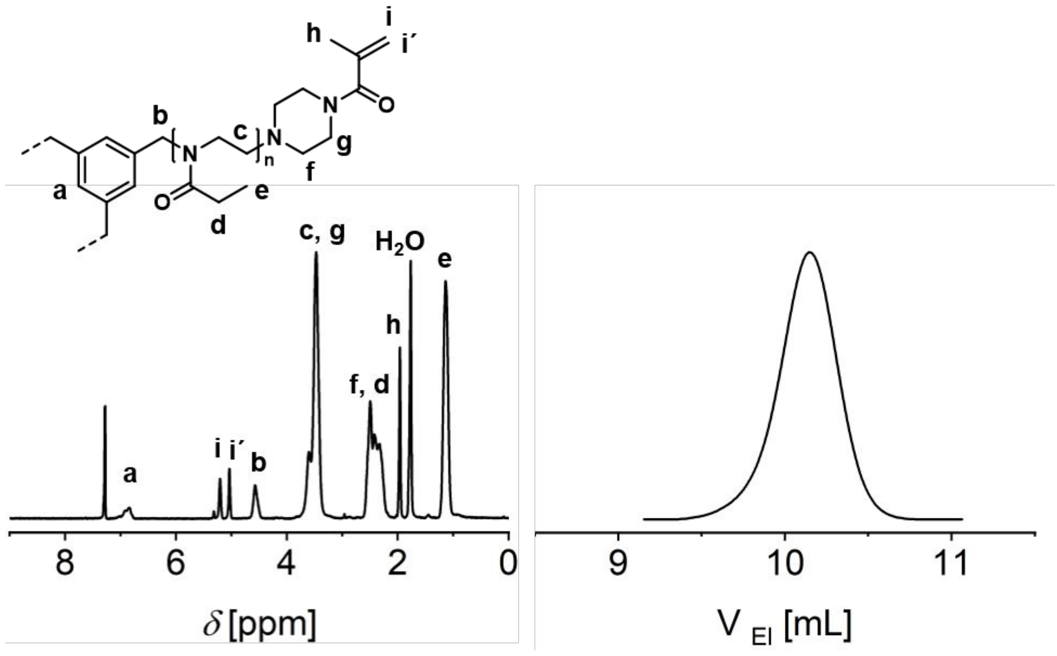

Figure S15. Characterization data of **T-Et-PipMA**. Left: ^{1}H NMR spectrum (300 MHz, $CDCl_3$). Right: SEC elugram (chloroform, triethylamine, *iso*-propanol, RI-detection).

7.2 Crosslinkers derived from the copolymerization of EtOx and MestOx

7.2.1 Bifunctional crosslinker with acrylate ω-end groups

B-Mest-A. The polymerization was performed for 2.5 h according to the general procedure using BIB (2.90 g 8 mmol, 1 eq.), EtOx (5.56 mL, 56 mmol, 7 eq.), MestOx (3.15 mL, 24 mmol, 3 eq.) dissolved in 34.4 mL of dry acetonitrile. The ω-chain ends were terminated by the addition of acrylic acid (2.20 mL, 32 mmol, 4 eq.) and triethylamine (4.30 mL, 32 mmol, 4 eq.) triethylamine at a reaction temperature of 50 °C. The polymer was obtained as a colorless solid. Yield: 5.96 g (64%).

^{1}H NMR ($CDCl_3$, 300 MHz): δ = 7.16 (br d, 4H, CH **a**), 6.31 - 6.56 (m, 2H, CH **j**), 6.01 - 6.27 (m, 2H, CH_2 **k**), 5.72 - 5.99 (m, 2H, CH_2 **k'**), 4.45 - 4.78 (m, 4H, CH_2 **b**), 4.06 - 4.40 (m, 4H, CH_2, **c′**, CH_2 **f′**), 2.99 - 3.84 (m, 47H, CH_3 **I**, CH_2 **c**, CH_2 **f**), 2.10 - 2.94 (m, 28H, CH_2 **g**, CH_2 **h**, CH_2 **d**), 0.88 - 1.29 (m, 24H, CH_3 **e**) ppm; DP = 10; DF = quant..

SEC (chloroform/*iso*-propanol/triethylamine [90/2/4; v/v/v], RI-detection, PS cal.): $M_{n,SEC}$ = 2010 g mol^{-1}; Đ = 1.17.

MALDI-TOF MS (DCTB + NaTFA): M_n = 1430 g mol^{-1}; Đ = 1.11; [$C_8H_8(C_5H_9NO)_7(C_7H_{11}NO_3)_3(C_3H_3O_2)_2 + Na^+$] = 1433.777 *m/z* (calculated 1433.779 *m/z*).

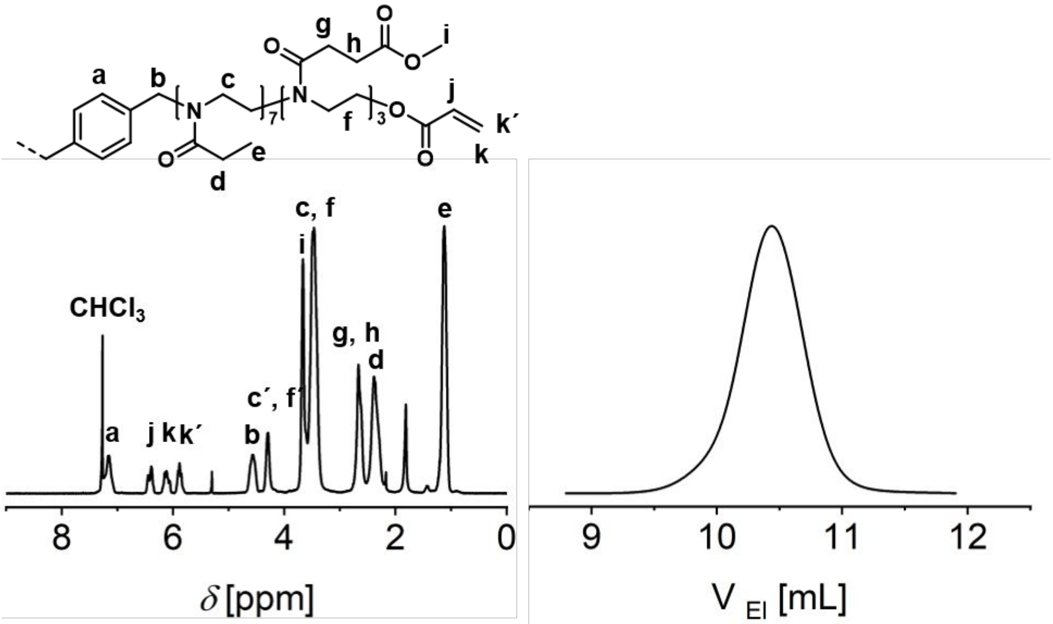

Figure S16. Characterization data of **B-Mest-A**. Left: ^{1}H NMR spectrum (300 MHz, $CDCl_3$). Right: SEC elugram (chloroform, triethylamine, *iso*-propanol, RI-detection).

7.2.2 Bifunctional crosslinker with methacrylate ω-end groups

B-Mest-MA. The polymerization was performed for 1.5 h according to the general procedure using BIB (5.83 g, 16 mmol, 1 eq.), EtOx (11.31 mL, 112 mmol, 7 eq.), MestOx (7.54 mL, 48 mmol, 3 eq.) and 61.15 mL dry acetonitrile. The ω-chain ends were terminated by the addition of methacrylic acid (5.30 mL, 60 mmol, 4 eq.) and 8.50 mL (63 mmol, 4 eq.) triethylamine at a reaction temperature of 50 °C. The polymer was obtained as a colorless solid. Yield: 15.34 g (83%).

^{1}H NMR ($CDCl_3$, 300 MHz): δ = 7.26 - 7.01 (m, 4H, CH **a**), 6.19 - 6.02 (m, 2H, CH_2 **k**), 5.69 - 5.67 (m, 1H), 5.61 (br d, 1H), 4.72 - 4.46 (m, 4H, CH_2 **b**), 4.28 (br s, 4H, CH_2 **c´**, CH_2 **f´**), 3.80 - 3.25 (m, 47H, CH_3 **i**, CH_2 **c**, CH_2 **f**), 2.65 (br d, 12H, CH_2 g, CH_2 **h**), 2.49 - 2.10 (m, 15H, CH_2 **d**), 1.94 (br s, 6H, CH_3 **j**), 1.13 (br s, 23H, CH_3 **e**) ppm; DP = 10; DF = 97%.

SEC (chloroform/*iso*-propanol/triethylamine [94/2/4, v/v/v], RI-detection, PS cal.): $M_{n,\,SEC}$ = 2030 g mol^{-1}; Đ = 1.18.

MALDI-TOF MS (DCTB + NaTFA): $M_{n,MALDI}$ = 1480 g mol^{-1}; Đ = 1.10; $[C_8H_8(C_5H_9NO)_7(C_7H_{11}NO_3)_3(C_3H_3O_2)_2 + Na^+]$ = 1461.805 *m/z* (calculated 1461.810 *m/z*).

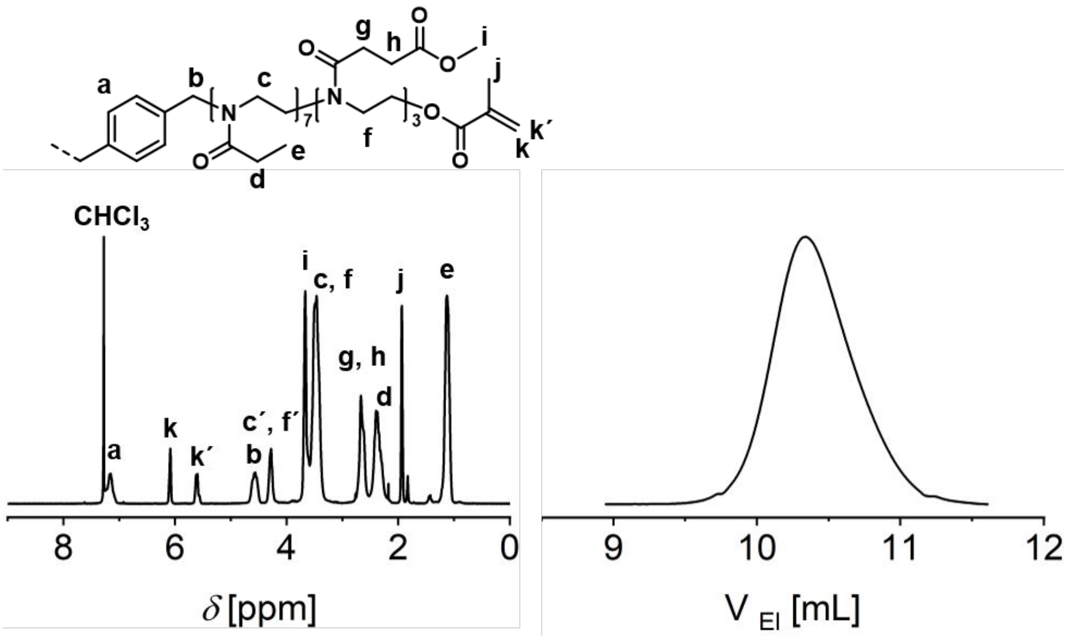

Figure S17. Characterization data of **B-Mest-MA**. Left: ^{1}H NMR spectrum (300 MHz, $CDCl_3$). Right: SEC elugram (chloroform, triethylamine, *iso*-propanol, RI-detection).

7.2.3 Bifunctional crosslinker with *N*-acryloyl-piperazine (PipA) ω-end groups

B-Mest-PipA. The polymerization was performed for 2.9 h according to the general procedure using BIB (1.08 g, 3 mmol, 1 eq.), EtOx (2.12 mL, 21 mmol, 7 eq.), MestOx (1.18 mL, 9 mmol, 3 eq.) and 26.76 mL dry acetonitrile. The ω-chain ends were terminated by the addition of PipA (2.13 g, 8.4 mmol, 2.8 eq.) and triethylamine (1.20 mL, 9 mmol, 3 eq.) at a reaction temperature of 30 °C. The polymer was obtained as a yellowish solid. Yield: 1.37 g (39%).

^{1}H NMR ($CDCl_3$, 300 MHz): δ = 7.27 - 6.98 (m, 4H, CH **a**), 6.68 - 6.46 (m, 2H, CH **l**), 6.38 - 6.23 (m, 2H, CH_2 **m**), 5.80 - 5.60 (m, 2H, CH_2 **m'**), 4.72 - 4.47 (m, 4H, CH_2 **b**), 3.93 - 3.22 (m, 52H, CH_3 **c**, CH_2 **c**, CH_2 **f**, CH_2 **k**), 3.07 - 2.03 (m, 40H, CH_2 **j**, CH_2 **g**, CH_2 **h**, CH_2 **d**), 1.13 (br s, 23H, CH_3 **e**) ppm; DP = 10; DF = 89%.

SEC (chloroform/*iso*-propanol/triethylamine [94/2/4, v/v/v], RI-detection, PS cal.): $M_{n,SEC}$ = 2420 g mol^{-1}; Đ = 1.25.

MALDI-TOF MS (DCTB + NaTFA): $M_{n,MALDI}$ = 1650 g mol^{-1}; Đ = 1.08; $[C_8H_8(C_5H_9NO)_{18}(C_7H_{11}NO_3)_2(C_7H_{11}N_2O_2)_2 + Na^+]$ = 1511.902 *m/z* (calculated 1511.921 *m/z*).

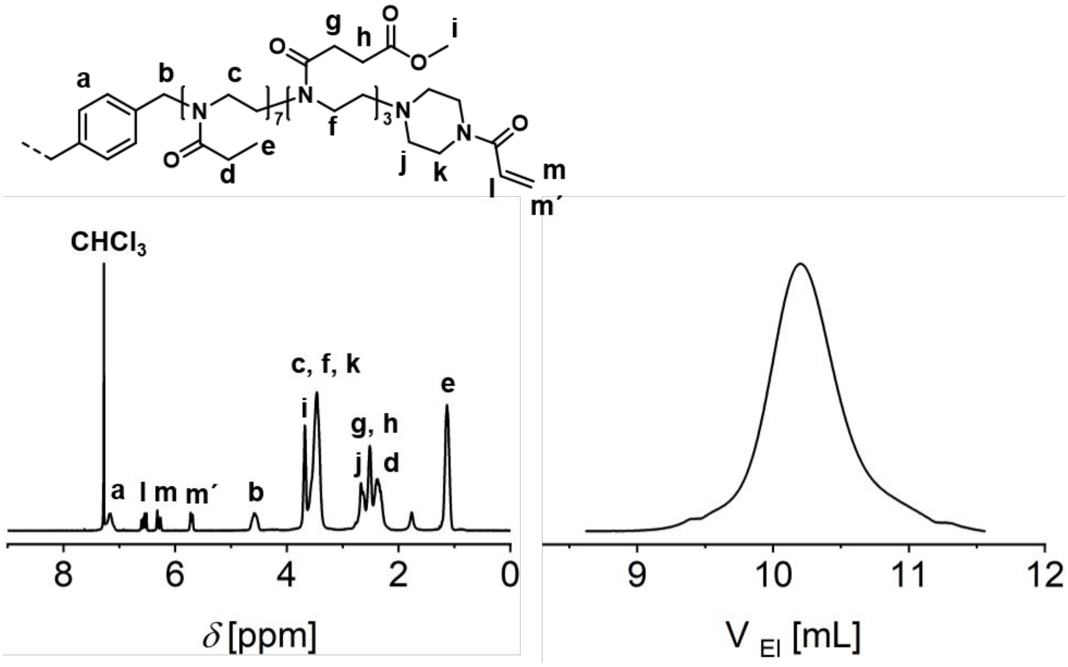

Figure S18. Characterization data of **B-Mest-PipA**. Left: ^{1}H NMR spectrum (300 MHz, $CDCl_3$). Right: SEC elugram (chloroform, triethylamine, *i*so-propanol, RI-detection).

7.2.4 Trifunctional crosslinker with acrylate ω-end groups

T-Mest-A. The polymerization was performed for 2.5 h according to the general procedure using TIB (1.99 g, 4 mmol, 1 eq.), EtOx (4.04 mL, 40 mmol, 7 eq.), MestOx (3.14 mL, 20 mmol, 3 eq.) and 22.82 mL dry acetonitrile. The ω-chain ends were terminated by the addition of acrylic acid (1.65 mL, 24 mmol, 6 eq.) and triethylamine (3.20 mL, 24 mmol, 6 eq.) at a reaction temperature of 50 °C. The polymer was obtained as a yellowish solid. Yield: 3.96 g (56%).

^1H NMR ($CDCl_3$, 300 MHz): δ = 7.18 - 6.71 (m, 3H, CH **a**), 6.59 - 6.30 (m, 3H, CH **j**), 6.23 - 6.01 (m, 3H, CH_2 **k**), 6.01 - 5.74 (m, 3H, CH_2 **k'**), 4.67 - 4.48 (m, 6H, CH_2 **b**), 4.84 - 4.46 (m, 6H, CH_2 **c'**, CH_2, **f'**), 3.85 - 3.20 (m, 72H, CH_3 **i**, CH_2 **c**, CH_2 **f**), 3.00 - 2.02 (m, 43H, CH_2 **g**, CH_2 **h**, CH_2 **d**), 1.13 (br s, 30H, CH_3 **e**); DP = 18; DF = 95%.

SEC (chloroform/*iso*-propanol/triethylamine [94/2/4, v/v/v], RI-detection, PS cal.): $M_{n,SEC}$ = 2930 g mol^{-1}; Đ = 1.20.

MALDI-TOF MS (DCTB + NaTFA): $M_{n,MALDI}$ = 1940 g mol^{-1}; Đ = 1.05 [$C_9H_9(C_5H_9NO)_9(C_7H_{11}NO_3)_4(C_3H_3O_2)_3 + Na^+$] = 1873.001 *m/z* (calculated 1873.011 *m/z*).

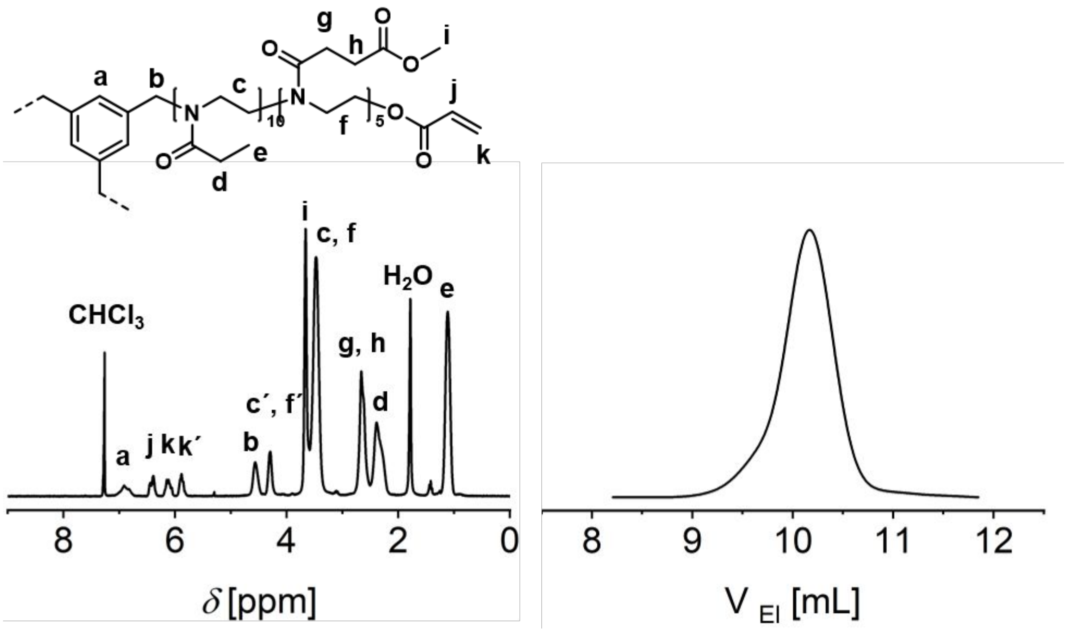

Figure S19. Characterization data of **T-Mest-A**. Left: ^1H NMR spectrum (300 MHz, $CDCl_3$). Right: SEC elugram (chloroform, triethylamine, *i*so-propanol, RI-detection).

7.2.5 Trifunctional crosslinker with methacrylate ω-end groups

T-Mest-MA. The polymerization was performed for 1.75 h according to the general procedure using TIB (6.64 g, 13 mmol, 1 eq.), EtOx (13.46 mL, 133 mmol, 10 eq.), MestOx (10.47 mL, 67 mmol, 5 eq.) and 76 mL dry acetonitrile. The ω-chain ends were terminated by the addition of (6.75 mL, 80 mmol, 6 eq.) methacrylic acid and triethylamine (10.7 mL, 80 mmol, 6 eq.) at

a reaction temperature of 50 °C. The polymer was obtained as a yellowish solid. Yield: 23.08 g (98%).

^{1}H NMR ($CDCl_3$, 300 MHz): δ = 7.10 - 6.67 (m, 3H, CH **a**), 6.20 - 5.97 (m, 3H, CH_2 **k**), 5.75 - 5.48 (m, 3H, CH_2 **k'**), 4.57 (br d, 6H, CH_2 **b**), 4.29 (br s, 6H, CH_2 **c'**, CH_2 **f'**), 3.98 - 3.10 (m, 70H, CH_3 **i**, CH_2 **c**, CH_2 **f**), 2.91 - 2.13 (m, 41H, CH_2 **g**, CH_2 **h**, CH_2 **d**), 1.13 (br s, 31H, CH_3 **e**) ppm; DP = 15; DF = 98%.

SEC (chloroform/*iso*-propanol/triethylamine [94/2/4, v/v/v], RI-detection, PS cal.): $M_{n,SEC}$ = 2920 g mol^{-1}; Đ = 1.12.

MALDI-TOF MS (DCTB + NaTFA): $M_{n,MALDI}$ = 2200 g mol^{-1}; Đ = 1.08; $[C_9H_9(C_5H_9NO)_{10}(C_7H_{11}NO_3)_5(C_4H_5O_2)_3 + Na^+]$ = 2171.181 *m/z* (calculated 2171.200 *m/z*).

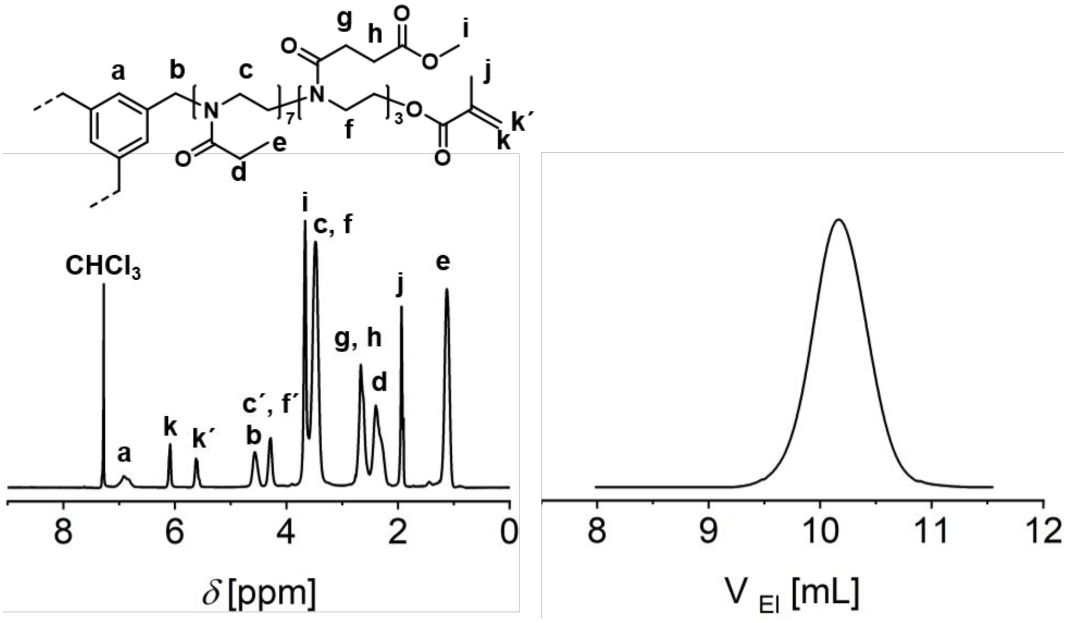

Figure S20. Characterization data of **T-Mest-MA**. Left: ^{1}H NMR spectrum (300 MHz, $CDCl_3$). Right: SEC elugram (chloroform, triethylamine, *i*so-propanol, RI-detection).

7.2.6 Trifunctional crosslinker with *N*-acryloyl-piperazine (PipA) ω-end groups

T-Mest-PipA. The polymerization was performed for 4.33 h according to the general procedure using TIB (1.18 g, 2.33 mmol, 1 eq.), EtOx (2.36 mL, 23 mmol, 7 eq.), MestOx (1.83 mL,

12 mmol, 3 eq.) and 30.8 mL dry acetonitrile yielding in an overall monomer concentration of 1 mol L^{-1}. The ω-chain ends were terminated by the addition of PipA (2.6 g, 12 mmol,4.4 eq.) and triethylamine (1.41 mL, 11 mmol, 4.5 eq.) at a reaction temperature of 30 °C. The polymer was obtained as a yellowish solid. Yield: 2.28 g (55%).

^{1}H NMR ($CDCl_3$, 300 MHz): δ = 7.11 - 6.71 (m, 4H, CH **a**), 6.68 - 6.47 (m, 3H, CH **l**), 6.39 - 6.22 (m, 3H, CH_2 **m**), 5.78 - 5.68 (m, 3H, CH_2 **m´**), 4.57 (br s, 8H, CH_2 **b**), 3.90 - 3.23 (m, 102H, CH_3 **i**, CH_2 **c**, CH_2 **f**, CH_2 **k**), 2.86 - 2.17 (m, 77H, CH_2 **d**, CH_2 **g**, CH_2 **h**, CH_2 **j**), 1.32 - 0.96 (m, 40H, CH_3 **e**) ppm; DP = 14; DF = 89%.

SEC (chloroform/*iso*-propanol/triethylamine [94/2/4, v/v/v], RI-detection, PS cal.): $M_{n,SEC}$ = 3330 g mol^{-1}; Đ = 1.26.

MALDI-TOF MS (DCTB + NaTFA): $M_{n,MALDI}$ = 2300 g mol^{-1}; Đ = 1.05; $[C_9H_9(C_5H_9NO)_{10}(C_7H_{11}NO_3)_4(C_7H_{11}N_2O)_3 + Na^+]$ = 2176.276 *m/z* (calculated 2176.301 *m/z*).

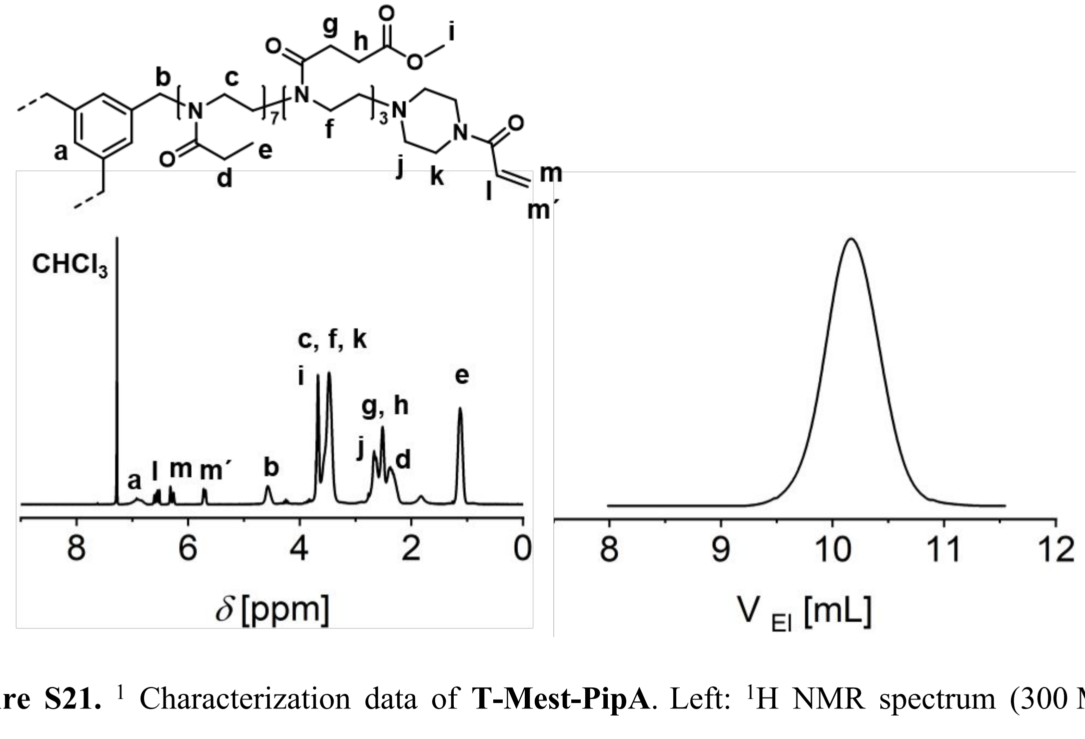

Figure S21. [1] Characterization data of **T-Mest-PipA**. Left: ^{1}H NMR spectrum (300 MHz, $CDCl_3$). Right: SEC elugram (chloroform, triethylamine, *i*so-propanol, RI-detection).

7.3 Crosslinkers derived from the copolymerization of EtOx and BocOx

7.3.1 Bifunctional crosslinker with acrylate ω-end groups

B-Boc-A. BIB (1.43 g, 4 mmol, 1 eq.), EtOx (3.03 mL, 30 mmol, 7.5 eq.) and BocOx (2.28 g, 10 mmol, 3 eq.) were suspended in 16.97 mL acetonitrile and stirred for 3 h at 50 °C. ^{1}H NMR spectroscopy confirmed that the polymerization had not taken place by that point. Then, the reaction mixture was heated to reflux for 4 h. The ω-chain ends were terminated by the addition of acrylic acid (0.411 mL, 6 mmol, 3 eq.) and triethylamine (1.07 mL, 8 mmol, 4 eq.) and the reaction mixture was stirred overnight at 50 °C. Purification was performed according to the general procedure. The polymer was obtained as a yellowish solid. Yield: 5.20 g (79%).

^{1}H NMR ($CDCl_3$, 300 MHz): δ = 7.16 (m, 4H, CH **a**), 6.44 - 6.39 (m, 2H, CH **j**), 6.15 - 6.06 (br, 2H, CH_2 **k**), 5.88 (br, 2H, CH_2 **k′**), 4.57 (br, 4H, CH_2 **b**), 4.29 (br, 4H, CH_2, **c′**, CH_2 **f′**) 3.62-3.46 (br, 38H, CH_2 **c**, CH_2 **f**), 3.16 (br, 6H, CH_2 **i**), 2.39 (br m, 20H, CH_2 **g**, CH_2 **d**), 1.82 (br, 6H, CH_2 **h**), 1.42 (br, 28H, CH_3 **Boc**), 1.13 (br, 23H, CH_3 **e**) ppm; DP = 10, DF = 94%.

SEC (chloroform/*iso*-propanol/triethylamine [94/2/4, v/v/v], RI-detection, PS cal.): $M_{n,SEC}$ = 1630 g mol^{-1}; Đ = 1.19.

MALDI-TOF MS (DCTB + NaTFA): $M_{n,MALDI}$ = 1470 g mol^{-1}; Đ = 1.13; [$C_8H_8(C_5H_9NO)_6(C_{11}H_{20}N_2O_3)_2(C_3H_3O_2)_3 + Na^+$] = 1319.812 *m/z* (calculated 1319.784 *m/z*).

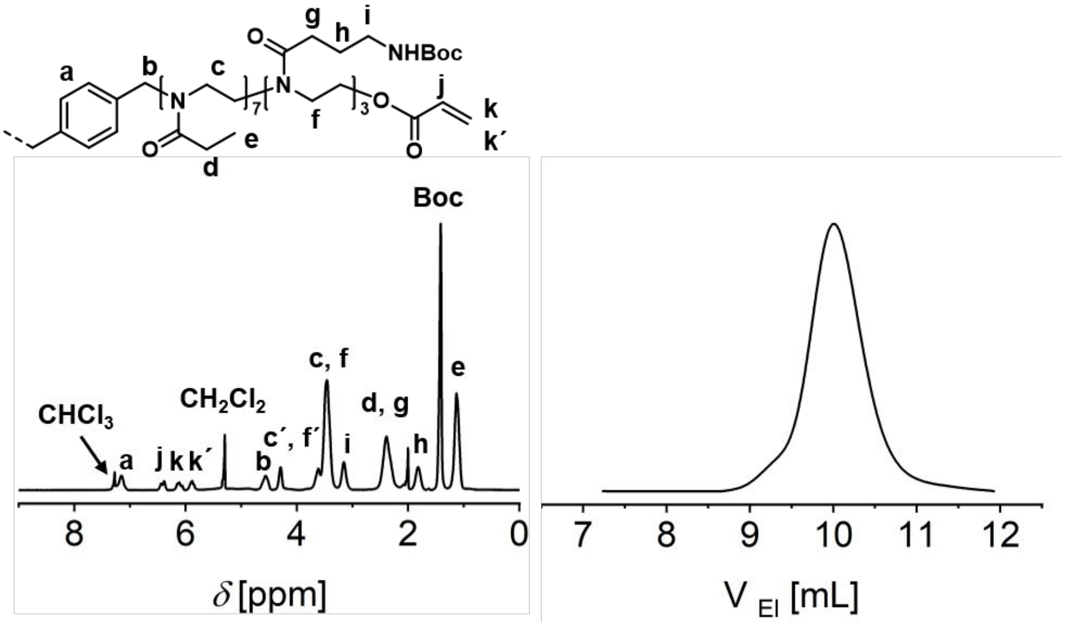

Figure S22. Characterization data of **B-Boc-A**. Left: ^{1}H NMR spectrum (300 MHz, $CDCl_3$). Right: SEC elugram (chloroform, triethylamine, *iso*-propanol, RI-detection).

.

7.3.2 Bifunctional crosslinker with methacrylate ω-end groups

B-Boc-MA. The polymerization was performed for 1.75 h according to the general procedure using BIB (6.37 g, 17.8 mmol, 1 eq.), EtOx (12.58 mL, 125 mmol, 7 eq.), BocOx (12.14 g, 534 mmol, 3 eq.) and 50 mL dry acetonitrile. The ω-chain ends were terminated by addition of methacrylic acid (4.34 mL, 53 mmol, 3 eq.) and triethylamine. (9.50 mL, 71 mmol, 4 eq.) at a reaction temperature of 50 °C. The polymer was obtained as a yellowish solid. Yield: 28.89 g (97%).

^{1}H NMR ($CDCl_3$, 300 MHz): δ = 7.17 (br, 4H, CH **a**), 6.10 (br, 2H, CH_2 **k**), 5.63 (br, 2H, CH_2 **k´**), 4.58 (br, 4H, CH_2 **b**), 4.30 (br, 4H, CH_2 **c´**,CH_2 **f´**), 3.64-3.47 (br, 38H, CH_2 **c**, CH_2 **f**), 3.18 (br, 6H, CH_2 **i**), 2.41 (br m, 20H, CH_2 **d**, CH_2 **g**), 1.95 (br, 6H, CH_3 **j**), 1.84 (br, 6H, CH_2 **h**), 1.43 (br, 28H, CH_3 **Boc**), 1.15 (br, 23H, CH_3 **e**) ppm; DP = 10; DF = 94%.

SEC (chloroform/*iso*-propanol/triethylamine [94/2/4, v/v/v], RI-detection, PS cal.): $M_{n,SEC}$ = 2020 g mol^{-1}; Đ = 1.13.

MALDI-TOF MS (DCTB + NaTFA): $M_{n,MALDI}$ = 1710 g mol^{-1}; Đ = 1.09, [$C_8H_8(C_5H_9NO)_7(C_{11}H_{20}N_2O_3)_3(C_4H_5O_2)_2 + Na^+$] = 1675.025 *m/z* (calculated 1675.031 *m/z*).

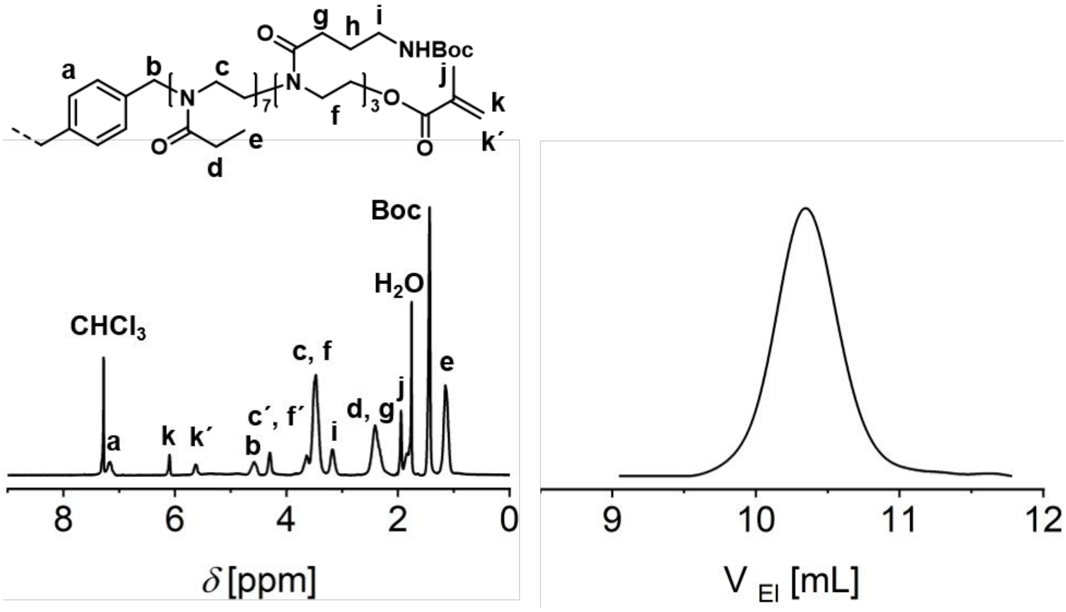

Figure S23. Characterization data of **B-Boc-MA**. Left: ^{1}H NMR spectrum (300 MHz, $CDCl_3$). Right: SEC elugram (chloroform, triethylamine, *iso*-propanol, RI-detection).

7.3.3 Bifunctional crosslinker with *N*-acryloyl-piperazine (PipA) ω-end groups

B-Boc-PipA. The polymerization was performed for 1.67 h according to the general procedure using BIB (3.22 g, 9 mmol, 1 eq.), EtOx (6.36 mL, 63 mmol, 7 eq.), BocOx (6.167 g, 27 mmol, 3 eq.) and 23.64 mL dry acetonitrile. The ω-chain ends were terminated by the addition of PipA (5.93 g, 23.4 mmol, 2.6 eq.) and triethylamine (3.61 mL, 27 mmol, 3 eq.) at room temperature. The polymer was obtained as a yellowish solid. Yield: 8.84 g (56%).

^{1}H NMR ($CDCl_3$, 300 MHz): δ = 7.15 (br, 4H, CH **a**), 6.60 - 6.51 (m, 2H, CH **l**), 6.31 - 6.25 (br, 2H, CH_2 **m**), 5.72 - 5.68 (br, 2H, CH_2 **m´**), 4.56 (br, 4H, CH_2 **b**), 3.68 - 3.45 (br, 44H, CH_2 **c**, CH_2 **f**, CH_2 **k**), 3.16 (br, 8H, CH_2 **i**), 2.51 - 2.35 (br m, 35H, CH_2 **d**, CH_2 **g**, CH_2 **j**), 1.83 (br, 8H, CH_2 **h**), 1.42 (br, 34H, CH_3 **Boc**), 1.13 (br, 23H, CH_3 **e**) ppm; DP = 11; DF = 84%.

SEC (chloroform/*iso*-propanol/triethylamine [94/2/4, v/v/v], RI-detection, PS cal.): $M_{n,\,SEC}$ = 2710 g mol^{-1}; Đ = 1.13.

MALDI-TOF MS (DCTB + NaTFA): $M_{n,MALDI}$ = 1810 g mol^{-1}; Đ = 1.07; $[C_8H_8(C_5H_9NO)_6(C_{11}H_{20}N_2O_3)_3(C_7H_{11}N_2O)_2 + Na^+]$ = 1684.068 *m/z* (calculated 1684.079 *m/z*).

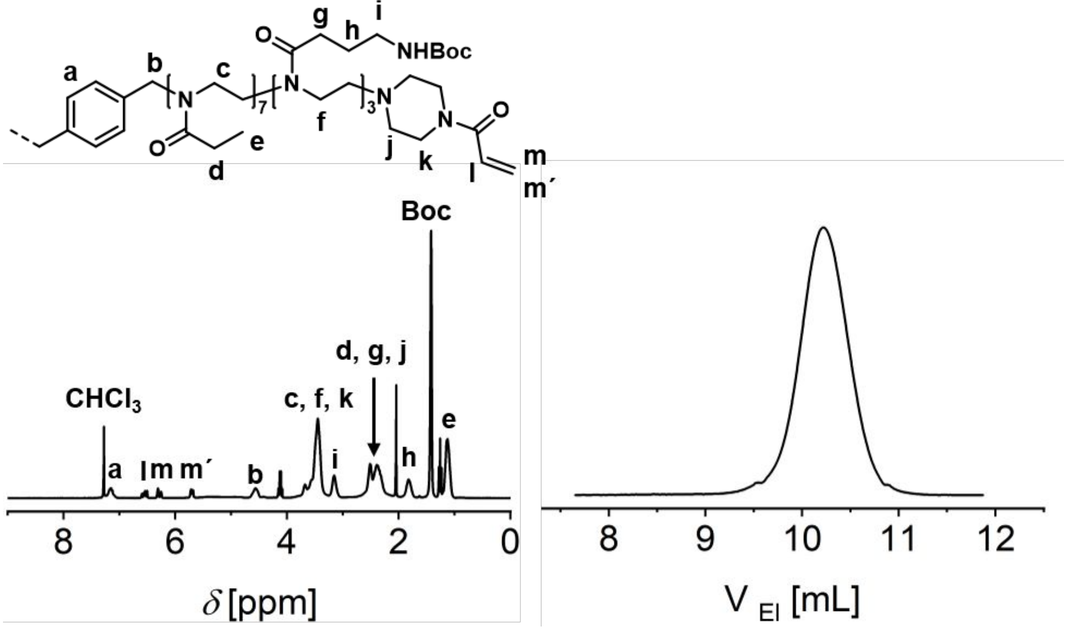

Figure S24. Characterization data of **B-Boc-PipA**. Left: ^{1}H NMR spectrum (300 MHz, $CDCl_3$). Right: SEC elugram (chloroform, triethylamine, *i*so-propanol, RI-detection).

7.3.4 Bifunctional crosslinker with *N*-methacryloyl-piperazine (PipMA) ω-end groups

B-Boc-PipMA. The polymerization was performed for 1.75 h according to the general procedure using BIB (5.36 g, 15 mmol, 1 eq.), EtOx (10.60 mL, 105 mmol, 7 eq.), BocOx (10.26 g, 45 mmol, 3 eq.) and 40 mL dry acetonitrile. The ω-chain ends were terminated by the addition of PipMA (6.94 g, 45 mmol, 3 eq.) and triethylamine (8.03 mL, 60 mmol, 4 eq.) at room temperature. The polymer was obtained as a yellowish solid. Yield: 25.42 g (94%).

^{1}H NMR ($CDCl_3$, 300 MHz): δ = 7.11 (br, 4H, CH **a**), 5.15 (br, 2H, CH_2 **m**), 4.98 (br, 2H, CH_2 **m´**), 4.53 (br, 4H, CH_2 **b**), 3.41 (br, 46H, CH_2 **c**, CH_2 **f**, CH_2 **k**), 3.12 (br, 7H, CH_2 **i**), 2.45 -

2.35 (br m, 34H, CH_2 **d**, CH_2 **g**, CH_2 **j**), 1.90 (br, 6H, CH_3 **l**), 1.79 (br, 7H, CH_2 **h**), 1.38 (br, 30H, CH_3 **Boc**), 1.09 (br, 22H, CH_3 **e**) ppm; DP = 10; DF = quant..

SEC (chloroform/*iso*-propanol/triethylamine [94/2/4, v/v/v], RI-detection, PS cal.): $M_{n,SEC}$ = 2630 g mol^{-1}; Đ = 1.10.

MALDI-TOF MS (DCTB + NaTFA): $M_{n,MALDI}$ = 1850 g mol^{-1}; Đ = 1.11; $[C_8H_8(C_5H_9NO)_7(C_{11}H_{20}N_2O_3)_3(C_8H_{13}N_2O)_2 + Na^+]$ = 1811.200 *m/z* (calculated 1811.179 *m/z*).

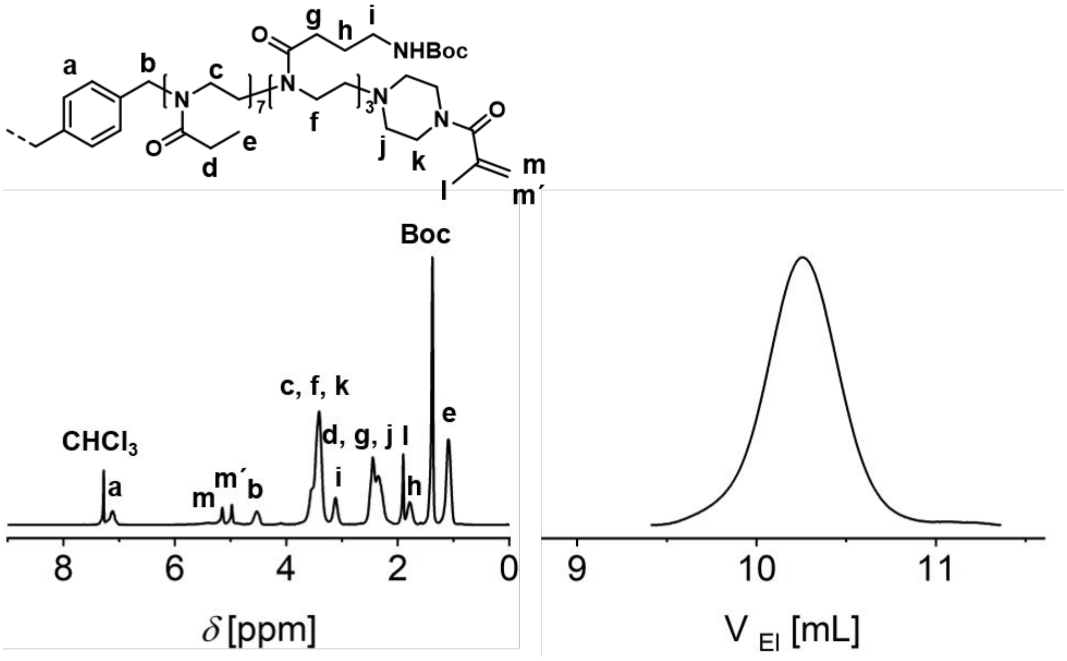

Figure S25. Characterization data of **B-Boc-PipMA**. Left: ^{1}H NMR spectrum (300 MHz, $CDCl_3$). Right: SEC elugram (chloroform, triethylamine, *i*so-propanol, RI-detection).

7.3.5 Trifunctional crosslinker with methacrylate ω-end groups

T-Boc-MA. The polymerization was performed for 2.08 h according to the general procedure using TIB (2.49 g, 5 mmol, 1 eq.), EtOx (5.05 mL, 50 mmol, 7 eq.), BocOx (5.73 g, 25 mmol, 3 eq.) and 20 mL dry acetonitrile. The ω-chain ends were terminated by the addition of methacrylic acid (5.6 mL, 22.5 mmol, 4.5 eq.) and triethylamine (4.02 mL, 30 mmol, 6 eq.) at

a reaction temperature of 50 °C. The polymer was obtained as a yellowish solid. Yield: 12.00 g (96%).

^{1}H NMR ($CDCl_3$, 300 MHz): δ = 6.93 - 6.83 (br, 3H, CH **a**), 6.09 (br, 3H, CH_2 **k**), 5.61 (br, 3H, CH_2 **k´**), 4.57 (br, 6H, CH_2 **b**), 4.28 (br, 6H, CH_2 **c´**, CH_2 **f´**), 3.63 - 3.46 (br, 51H, CH_2 **c**, CH_2 **f**), 3.16 (br, 10H, CH_2 **i**), 2.40 - 2.32 (br m, 30H, CH_2 **d**, CH_2 **g**), 1.94 (br, 9H, CH_3 **j**), 1.83 (br, 10H, CH_2 **h**), 1.42 (br, 43H, CH_3 **Boc**), 1.14 (br, 30H, CH_3 **e**) ppm; DP = 15; DF = 98%.

SEC (chloroform/*iso*-propanol/triethylamine [94/2/4, v/v/v], RI-detection, PS cal.): $M_{n,SEC}$ = 3230 g mol^{-1}; Đ = 1.11.

MALDI-TOF MS (DCTB + NaTFA): $M_{n,MALDI}$ = 2220 g mol^{-1}; Đ = 1.06; [$C_9H_9(C_5H_9NO)_9(C_{11}H_{20}N_2O_3)_4(C_4H_5O_2)_3$ + Na^+] = 2199.311 *m/z* (calculated 2199.351 *m/z*).

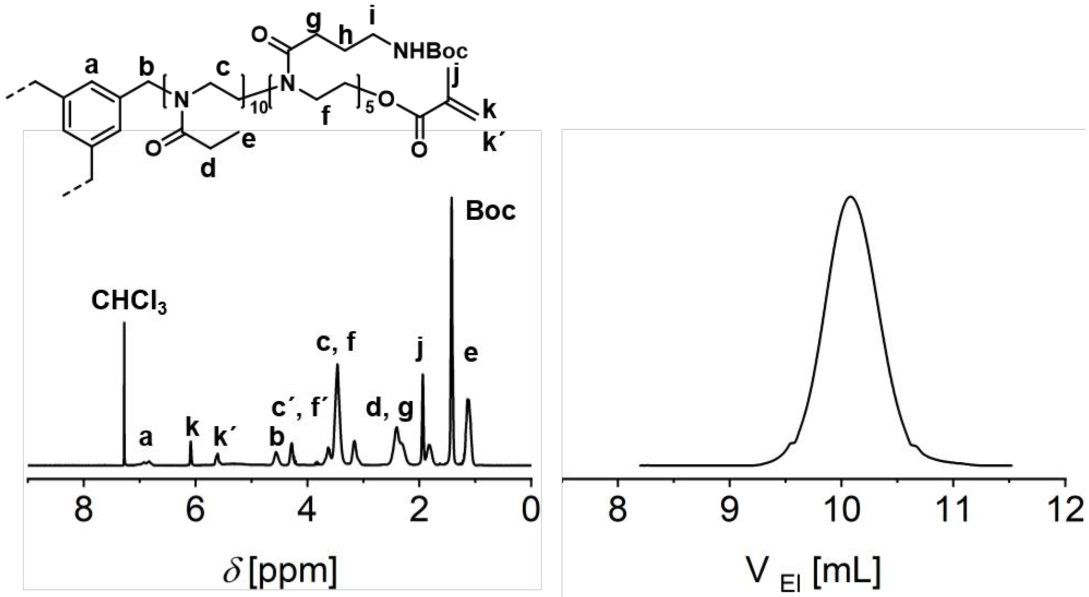

Figure S26. Characterization data of **T-Boc-MA**. Left: ^{1}H NMR spectrum (300 MHz, $CDCl_3$). Right: SEC elugram (chloroform, triethylamine, *i*so-propanol, RI-detection).

7.3.6 Trifunctional crosslinker with *N*-acryloyl-piperazine (PipA) ω-end groups

T-Boc-PipA. The polymerization was performed for 1.5 h according to the general procedure using TIB (2.49 g, 5 mmol, 1 eq.), EtOx (5.05 mL, 50 mmol, 7 eq.), BocOx (5.73 g, 25 mmol,

3 eq.) and 20 mL dry acetonitrile. The ω-chain ends were terminated by the addition of PipA (4.95 g, 19.5 mmol, 3.9 eq.) and triethylamine (3.01 mL, 22.5 mmol, 4.5 eq.) at room temperature. The polymer was obtained as a yellowish solid. Yield: 7.70 g (71%).

^{1}H NMR ($CDCl_3$, 300 MHz): δ = 6.90 - 6.80 (br, 3H, CH **a**), 6.61 - 6.52 (m, 3H, CH **l**), 6.33 - 6.27 (br, 3H, CH_2 **m**), 5.73 (br, 3H, CH_2 **m´**), 4.58 (br, 6H, CH_2 **b**), 3.70 - 3.47 (br, 62H, CH_2 **c**, CH_2 **f**, CH_2 **k**), 3.17 (br, 12H, CH_2 **i**), 2.53 - 2.34 (br m, 43H, CH_2 **d**, CH_2 **g**, CH_2 **j**), 1.84 (br, 16H, CH_2 **h**), 1.43 (br, 47H, CH_3 **Boc**), 1.14 (br, 29H, CH_3 **e**) ppm; DP = 15; DF = 82%.

SEC (chloroform/*iso*-propanol/triethylamine [94/2/4, v/v/v], RI-detection, PS cal.): $M_{n,SEC}$ = 3190 g mol^{-1}; Đ = 1.18.

MALDI-TOF MS (DCTB + NaTFA): $M_{n,MALDI}$ = 2490 g mol^{-1}; Đ = 1.07; $[C_9H_9(C_5H_9NO)_{10}(C_{11}H_{20}N_2O_3)_4(C_7H_{11}N_2O)_3 + Na^+]$ = 2460.598 *m/z* (calculated 2460.595 *m/z*).

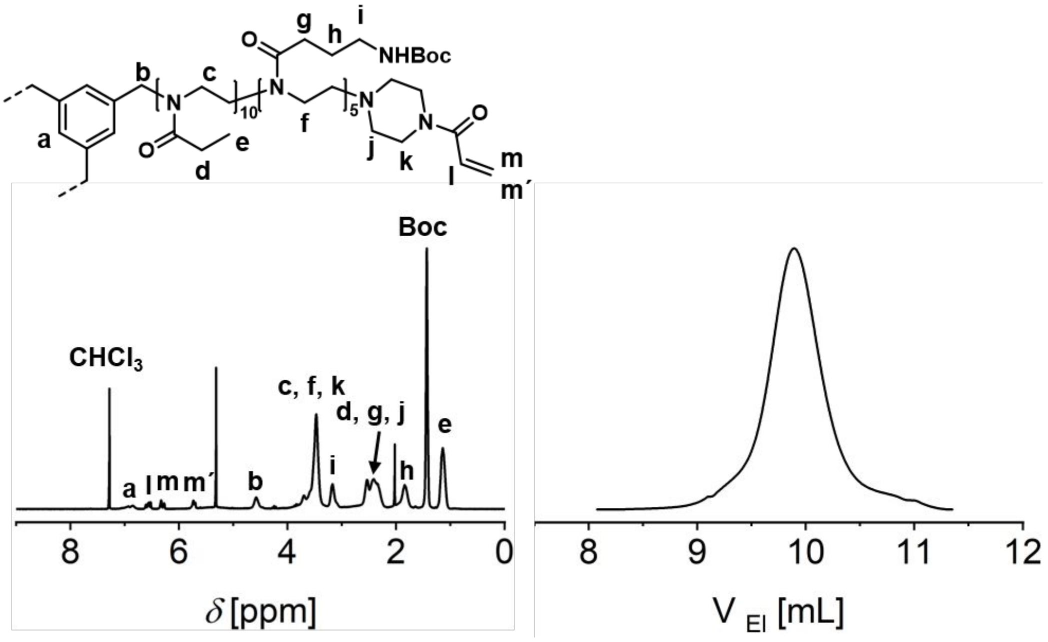

Figure S27. Characterization data of **T-Boc-PipA**. Left: ^{1}H NMR spectrum (300 MHz, $CDCl_3$). Right: SEC elugram (chloroform, triethylamine, *i*so-propanol, RI-detection).

7.4 Deprotection of BocOx containing polymers yielding crosslinkers with amino moieties

7.4.1 Bifunctional crosslinker with acrylate ω-end groups

B-Am-A. B-Boc-A (1 g, 0.31 mmol, 1 eq.) was dissolved in dist. dichloromethane and TFA (4.0 mL, 52.3 mmol, 168 eq., 56 eq. per Boc-group) was added. The reaction mixture was stirred for 1 h at room temperature. The deprotected polymer separated as a liquid from the dichloromethane phase. The layer containing the deprotected polymer was separated and volatiles were evaporated under reduced pressure. The residue was dissolved in water and passed through a column filled with the chloride-loaded anion exchange resin Ambersep 900 (compare materials section). A sample was taken, freeze-dried, and analyzed by ^{1}H NMR spectroscopy, which indicated the loss of the acrylate end groups.

7.4.2 Bifunctional crosslinker with methacrylate ω-end groups

B-Am-MA. B-Boc-MA (12.00 g, 7.4 mmol, 1 eq.) was dissolved in 120 mL dichloromethane and TFA (17 mL, 221.6 mmol, 30 eq., 10 eq. per Boc-group) was added. The reaction mixture was stirred for 2 h at room temperature. The deprotected polymer separated as a liquid from the dichloromethane phase. The layer containing the deprotected polymer was separated and volatiles were evaporated under reduced pressure. The residue was dissolved in water and passed through a column filled with the chloride-loaded anion exchange resin Ambersep 900 (compare materials section). The combined aqueous fractions were lyophilized. The polymer was obtained as a yellowish solid. Yield: 6.89 g (57%).

^{1}H NMR (D_2O, 300 MHz): δ = 7.16 (br, 4H, CH **a**), 5.96 (br, 2H, CH_2 **k**), 5.60 (br, 2H, CH_2 **k′**), 4.53 (br, 4H, CH_2 **b**), 4.23 (br, 4H, CH_2 **c′**, CH_2 **f′**), 3.62-3.40 (br, 44H, CH_2 **c**, CH_2 **f**), 2.90 (br, 8H, CH_2 **i**), 2.36 - 2.23 (br m, 22H, CH_2 **d**, CH_2 **g**), 1.81-1.77 (br, 13H, CH_2 **h**, CH_3 **j**), 0.92 (br, 21H, CH_3 **e**) ppm; DP = 10 DF = 84%.

MALDI-TOF MS (CHCA): $M_{n,MALDI}$ = 1520 g mol^{-1}; Đ = 1.06; $[C_8H_8(C_5H_9NO)_{10}(C_6H_{12}N_2O)_3(C_4H_5O_2)_2 + Na^+]$ = 1672.203 *m/z* (calculated 1672.079 *m/z*).

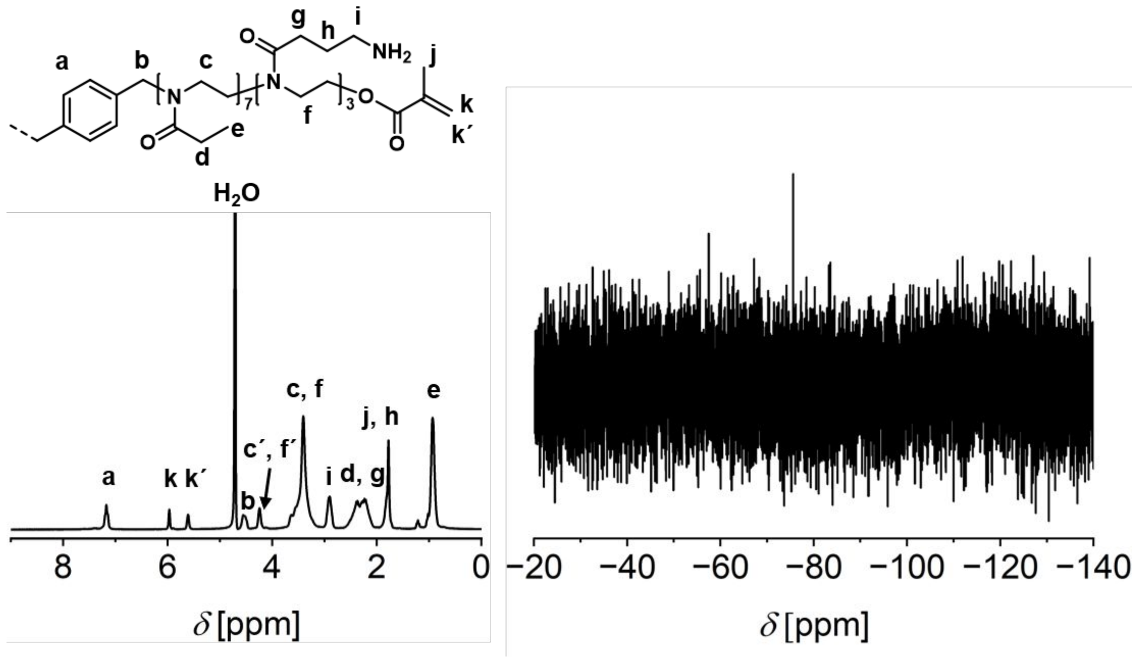

Figure S28. ^{1}H NMR spectrum (left) and ^{19}F NMR spectrum (right) (300 MHz, D_2O) of **B-Am-MA** (300 MHz, D_2O).

7.4.3 Bifunctional crosslinker with *N*-acryloyl-piperazine (PipA) ω-end groups

B-Am-PipA. **B-Boc-PipA** (2.40 g, 1.36 mmol, 1 eq.) was dissolved in 50 mL dist. dichloromethane and TFA (6.2 mL, 81.0 mmol, 50 eq., 17 eq. per Boc-group) was added. The reaction mixture was stirred for 2 h at room temperature and the solvent was evaporated. The residue was re-dissolved in acetone and precipitated thrice from –80 °C diethylether. The precipitate was dried *in vacuo* and the polymer was obtained as a yellowish solid. Yield: 1.78 g (74%).

^{1}H NMR (D_2O, 300 MHz): δ = 7.19 (br, 4H, CH **a**), 6.66 - 6.57 (m, 2H, CH_2 **l**), 6.16 - 6.11 (br, 2H, CH_2 **m**), 5.82-5.78 (br, 2H, CH_2 **m´**), 4.59 (br, 4H, CH_2 **b**), 3.90 - 3.30 (br, 54H, CH_2 **c**, CH_2 **f**, CH_2 **j**, CH_2 **k**), 2.93 (br, 8H, CH_2 **i**), 2.31 (br m, 22H, CH_2 **d**, CH_2 **i**), 1.83 (br, 8H, CH_2 **h**), 0.95 (br, 21H, CH_3 **e**) ppm; DP = 10; DF = 82%.

MALDI-TOF MS (CHCA): $M_{n,MALDI}$ = 1800 g mol^{-1}; Đ = 1.06; [$C_8H_8(C_5H_9NO)_9(C_6H_{12}N_2O)_3(C_7H_{11}N_2O)_2 + Na^+$] = 1681.113 *m/z* (calculated 1681.127 *m/z*).

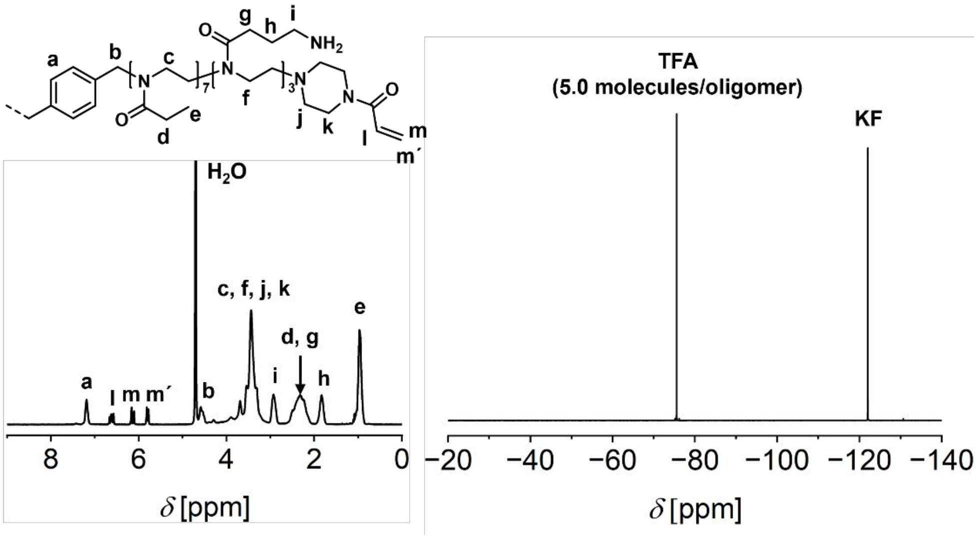

Figure S29. ^1H NMR spectrum (left)- and ^{19}F NMR spectrum (right) (300 MHz, D_2O) of **B-Am-PipA** (300 MHz, D_2O).

7.4.4 Bifunctional crosslinker with *N*-methacryloyl-piperazine (PipMA) ω-end groups

B-Am-PipMA. B-Boc-PipMA (12.00 g, 7.04 mmol, 1 eq.) was dissolved in 120 mL dichloromethane and TFA (20 mL, 259.6 mmol, 37 eq., 12 eq. per Boc-group) was added. The reaction mixture was stirred for 4 h at room temperature. The deprotected polymer separated as a liquid from the dichloromethane phase. The layer containing the deprotected polymer was separated and volatiles were evaporated under reduced pressure. The residue was dissolved in water and the pH value was adjusted to 8 using aq. NaOH (1 M) solution. The aqueous phase was extracted with chloroform thrice. The combined organic layers were dried over Na_2SO_4 and the solvent was removed under reduced pressure. The polymer was obtained as a yellowish solid. Yield: 5.80 g (48%).

^1H NMR (D_2O, 300 MHz): δ = 7.25 (br, 4H, CH **a**), 5.26 (br, 2H, CH **m**), 5.06 (br, 2H, CH_2 **m´**), 4.60 (br, 4H, CH_2 **b**), 3.59 - 3.48 (br, 42H, CH_2 **c**, CH_2 **f**, CH_2 **j**, CH_2 **k**, CH_2 **i**), 2.57 - 2.18 (br m, 36H, CH_2 **d**, CH_2 **g**), 1.87 (br, 6H, CH_3 **l**), 1.65 (br, 5H, CH_2 **h**) 0.96 (br, 21H, CH_3 **e**) ppm; DP = 10; DF = 94%.

MALDI-TOF MS (CHCA): $M_{n,MALDI}$ = 1740 g mol^{-1}; Đ = 1.06; $[C_8H_8(C_5H_9NO)_9(C_6H_{12}N_2O)_3(C_8H_{13}N_2O)_2 + Na^+]$ = 1709.263 *m/z* (calculated 1709.158 *m/z*) observed.

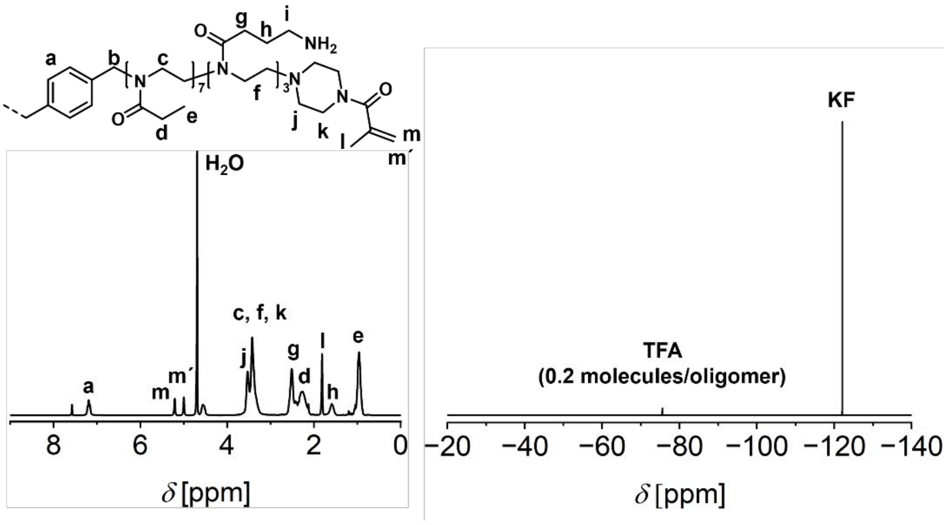

Figure S30. ^{1}H NMR spectrum (left) and ^{19}F NMR spectrum (right) (300 MHz, D_2O) of **B-Am-PipMA**.

7.4.5 Trifunctional crosslinker with methacrylate ω-end groups

T-Am-MA. T-Boc-MA (8.00 g, 3.19 mmol, 1 eq.) was dissolved in 20 mL dichloromethane and TFA (8.53 mL, 111.48 mmol, 35 eq., 7 eq. per Boc-group) was added. The reaction mixture was stirred for 2 h at room temperature. The deprotected polymer separated as a liquid from the dichloromethane phase. The layer containing the deprotected polymer was separated and volatiles were evaporated under reduced pressure. The residue was dissolved in water and the pH value was adjusted to 8 using aq. NaOH (1 M) solution. The aqueous phase was extracted with chloroform thrice. The combined organic layers were dried over Na_2SO_4 and the solvent was removed under reduced pressure. The polymer was obtained as a yellowish solid. Yield: 1.42 g (18%).

^{1}H NMR (D_2O, 300 MHz): δ = 6.89 (br, 3H, CH **a**), 5.99 (br, 3H, CH_2 **k**), 5.62 (br, 3H, CH_2 **k′**), 4.57 (br, 6H, CH_2 **b**), 4.26 (br, 6H, CH_2 **c′**, CH_2 **f′**), 3.62 - 3.42 (br, 45H, CH_2 **c**, CH_2 **f**), 2.65

(br, 5H, CH_2 **i**), 2.39 - 2.26 (br m, 25H, CH_2 **d**, CH_2 **g**), 1.80 - 1.65 (br, 13H, CH_2 **h**, CH_3 **j**), 0.98 (br, 30H, CH_3 **e**) ppm; DP = 13; DF = 86%.

MALDI-TOF MS (CHCA): $M_{n,MALDI}$ = 1660 g mol^{-1}; Đ = 1.07; repeating units of EtOx and AmOx were found but the envisaged end group could not be confirmed.

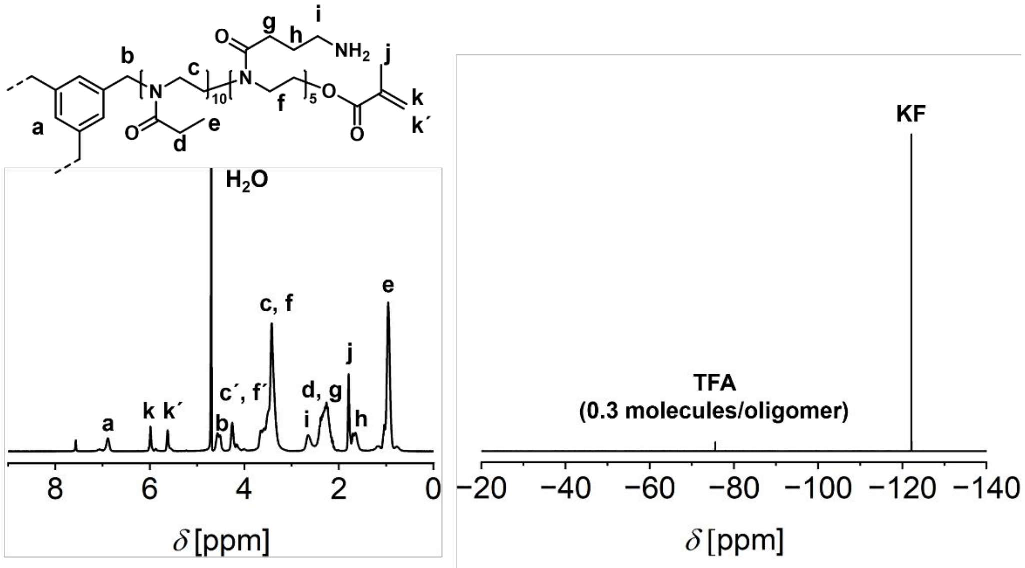

Figure S31. ^{1}H NMR spectrum (left) and ^{19}F NMR spectrum (right) (300 MHz, D_2O) of **T-Am-MA**.

7.4.6 Trifunctional crosslinker with *N*-acryloyl-piperazine (PipA) ω-end groups

T-Am-PipA. T-Boc-PipA (2.54 g, 0.95 mmol, 1 eq.) was dissolved in 50 mL dichloromethane and TFA (7.1 mL, 92.7 mmol, 98 eq., 20 eq. per Boc-group) was added. After 3 h of continuous stirring at room temperature, the solvent was evaporated. The residue was re-dissolved in acetone and precipitated five times from –80 °C diethylether. The polymer was dried *in vacuo*. The polymer was obtained as a yellowish solid. Yield: 2.13 g (84%).

^{1}H NMR (D_2O, 300 MHz): δ = 6.84 (br, 3H, CH **a**), 6.61 - 6.57 (m, 3H, CH_2 **l**), 6.16 - 6.11 (br, 3H, CH_2 **m**), 5.81-5.78 (br, 3H, **m´**), 4.58 (br, 6H, CH_2 **b**), 3.69 - 3.31 (br, 91H, CH_2 **c**, CH_2 **f**, CH_2 **j**, CH_2 **k**), 2.93 (br, 12H, CH_2 **i**), 2.49 - 2.23 (br m, 34H, CH_2 **d**, CH_2 **g**), 1.83 (br, 12H, CH_2 **h**), 0.97 (br, 33H, CH_3 **e**) ppm; DP = 17; DF = 88%.

MALDI-TOF MS (CHCA): $M_{n,MALDI}$ = 2320 g mol^{-1}; Đ = 1.04; repeating units of EtOx and AmOx were found but the envisaged end group could not be confirmed.

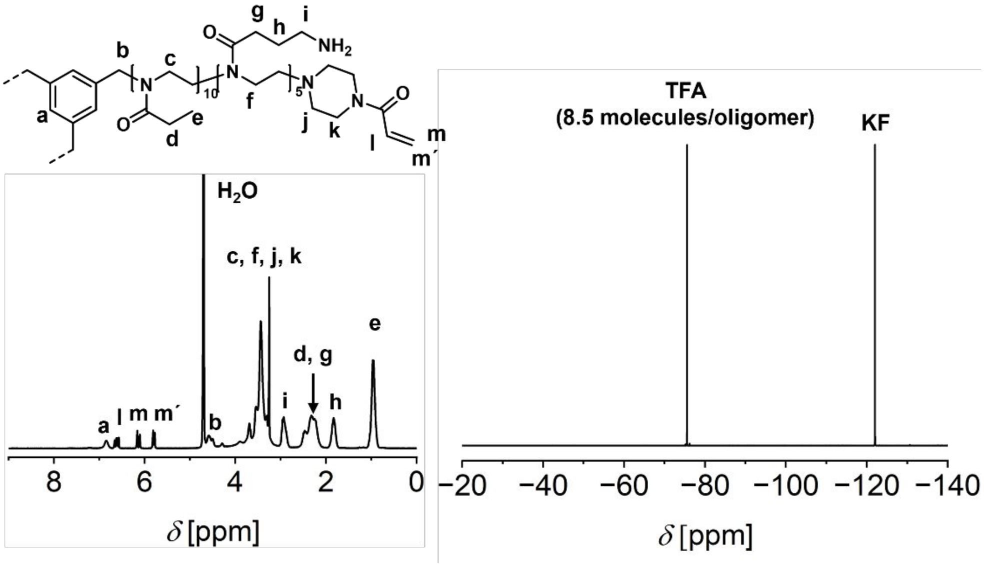

Figure S32. ^{1}H NMR spectrum (left) and ^{19}F NMR spectrum (right) (300 MHz, D_2O) of **T-Am-PipA**.

8 CRYOGEL PREPARATION

8.1 Bifunctional EtOx-based cryogels

Table S2. Overview about the conditions and parameters for the preparation of cryogels using bifunctional EtOx-based crosslinkers (CL). The amounts of monomers and $K_2S_2O_8$ are given per 8 mL which corresponds to one duplicate of 5 mL syringes each. Individual volume in each syringe was 3.98 mL after addition of TMEDA. t_R = reaction time. Proportion of rhodamine B acrylate (RhoB) was approx. 0.2%. [M] = monomer concentration in the syringe. [I] = initiator concentration in the syringe.

CL	CL amount	RhoB	$K_2S_2O_8$	t_R [h]	[M] [mol L^{-1}]	[I] [mol L^{-1}]
B-Et-A	290 mg 0.227 mmol	8 mL of 0.4 mM; 0.003 mmol	8 mL of 11.1 mM; 0.089 mmol	19.7	0.028	0.011
	287 mg 0.224 mmol	8 mL of 0.4 mM; 0.003 mmol	8 mL of 11.1 mM; 0.089 mmol	19.7	0.028	0.011
B-Et-MA	315 mg 0.225 mmol	8 mL of 0.5 mM; 0.004 mmol	8 mL of 11.3 mM; 0.090 mmol	17.8	0.032	0.011
B-Et-PipMA	317 mg 0.226 mmol	8 mL of 0.4 mM; 0.003 mmol	8 mL of 11.3 mM; 0.090 mmol	24	0.028	0.011
B-Et-PipA	309 mg 0.225 mmol	8 mL of 0.4 mM; 0.003 mmol	8 mL of 11.8 mM; 0.094 mmol	22.2	0.028	0.012
	310 mg 0.226 mmol	8 mL of 0.4 mM; 0.003 mmol	8 mL of 11.8 mM; 0.094 mmol	22.2	0.028	0.012

8.2 Trifunctional EtOx-based cryogels

Table S3. Overview about the conditions and parameters for the preparation of cryogels using trifunctional EtOx-based crosslinkers (CL). The amounts of monomers and $K_2S_2O_8$ are given per 8 mL which corresponds to one duplicate of 5 mL syringes each. Individual volume in each syringe was 3.98 mL after addition of TMEDA. t_R = reaction time. Proportion of rhodamine B acrylate (RhoB) was approx. 0.2%. [M] = monomeric concentration in the syringe. [I] = initiator concentration in the syringe.

CL	CL amount	RhoB	$K_2S_2O_8$	t_R [h]	[M] [mol L^{-1}]	[I] [mol L^{-1}]
T-Et-A	418 mg<br>0.226 mmol	8 mL of 0.4 mM;<br>0.003 mmol	8 mL of 11.1 mM;<br>0.089 mmol	19.5	0.028	0.011
	418 mg<br>0.226 mmol	8 mL of 0.4 mM;<br>0.003 mmol	8 mL of 11.1 mM;<br>0.089 mmol	19.5	0.028	0.011
T-Et-MA	427 mg<br>0.225 mmoL	8 mL of 0.4 mM;<br>0.003 mmol	8 mL of 11.1 mM;<br>0.089 mmol	22.5	0.028	0.011
T-Et-PipMA	450 mg<br>0.225 moL	8 mL of 0.4 mM;<br>0.003 mmol	8 mL of 11.1 mM;<br>0.089 mmol	24	0.028	0.011
T-Et-PipA	455 mg<br>0.225 mmol	8 mL of 0.4 mM;<br>0.003 mmol	8 mL of 11.1 mM;<br>0.089 mmol	21.6	0.028	0.011
	456 mg<br>0.226 mmol	8 mL of 0.4 mM;<br>0.003 mmol	8 mL of 11.1 mM;<br>0.089 mmol	22	0.028	0.011

9 ADDITIONAL SUPPORTING FIGURES

9.1 MALDI-TOF mass spectra

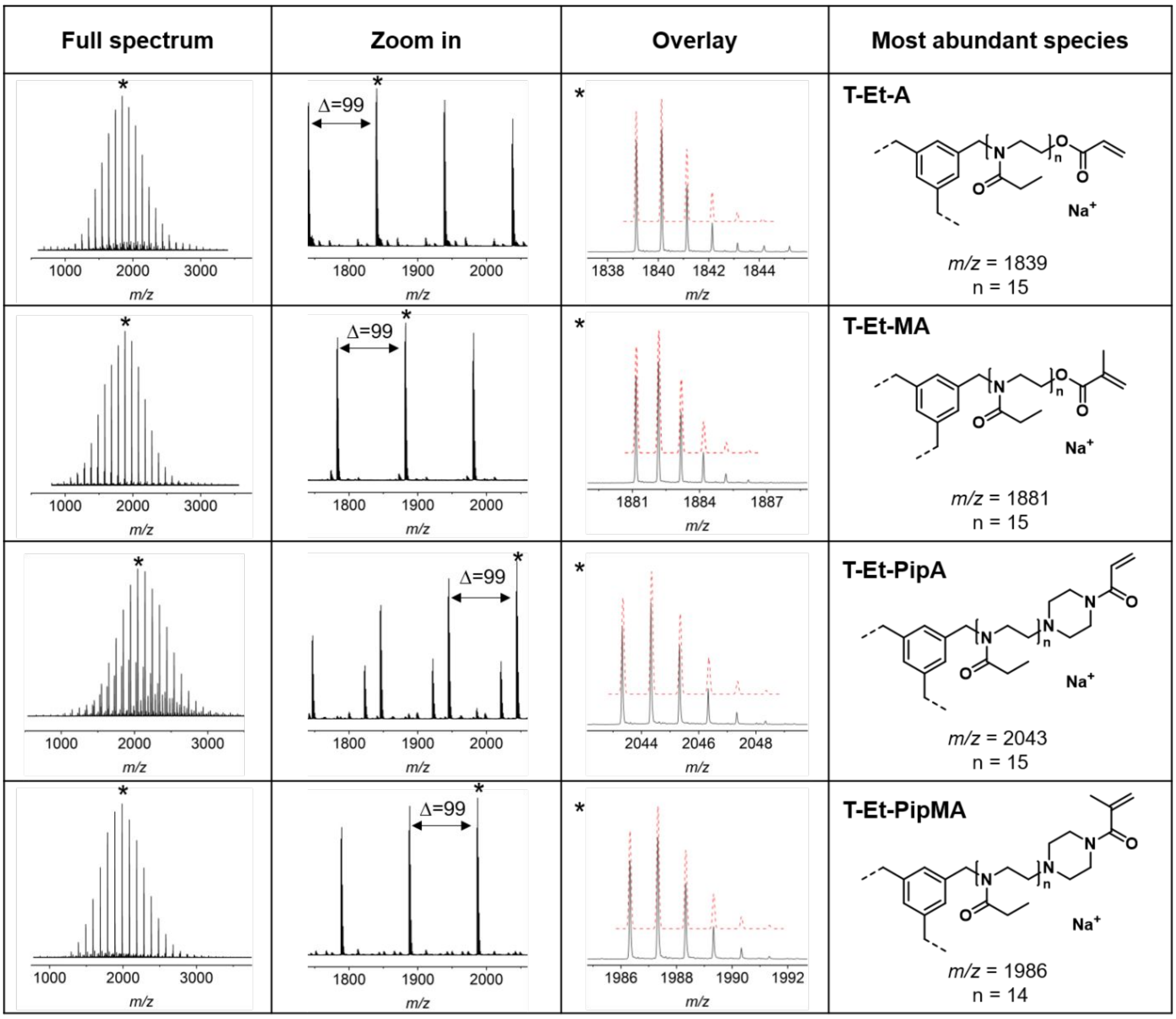

Figure S33. Full MALDI-TOF mass spectra (DCTB + NaTFA) of the crosslinkers derived from the homopolymerization of EtOx using the trifunctional initiator TIB. From left to right: Full spectra, zoom into an *m/z* region displaying the EtOx repeating units, overlay of the measured and calculated (red dotted line) isotopic pattern of the most abundant species (marked with *). All identified *m/z* species were found as sodium adducts.

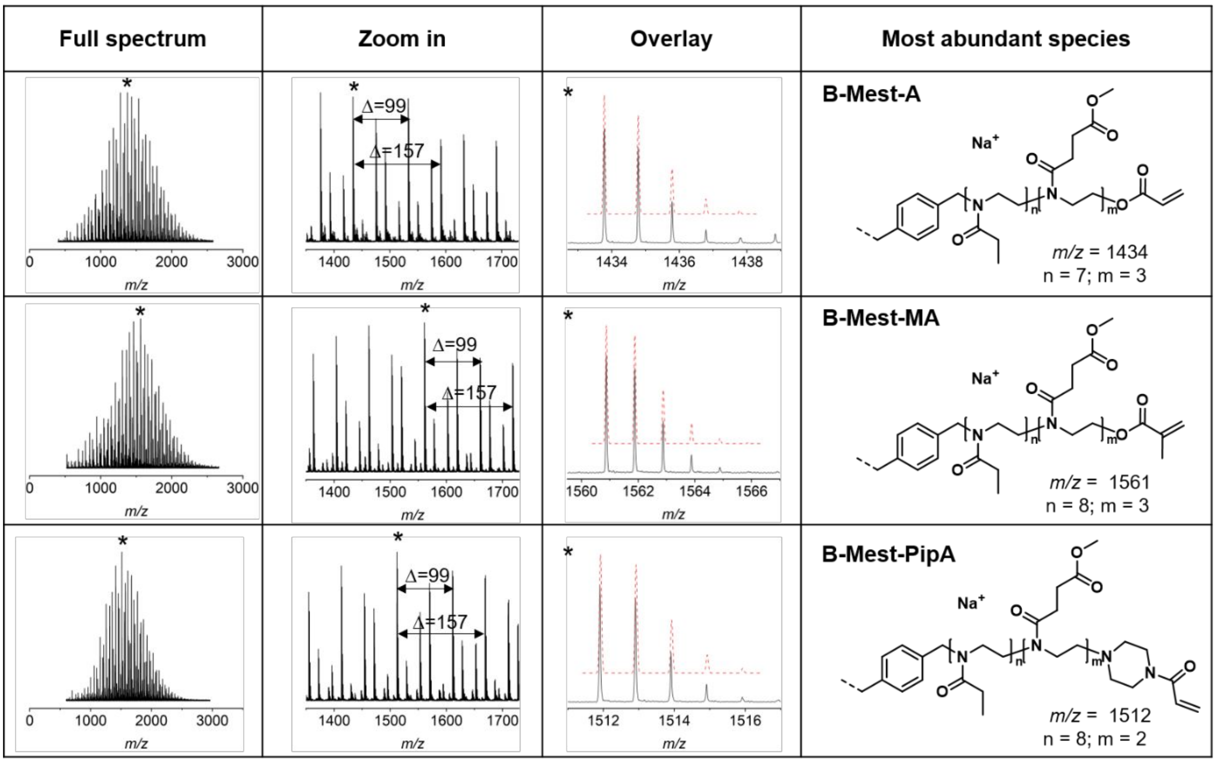

Figure 34. Full MALDI-TOF mass spectra (DCTB + NaTFA) of the crosslinkers derived from the copolymerization of EtOx and MestOx using the bifunctional initiator BIB. From left to right: Full spectra, zoom into an *m/z* region displaying the EtOx as well as the MestOx repeating units, overlay of the measured and calculated (red dotted line) isotopic pattern of the most abundant species (marked with *). All identified *m/z* species were found as sodium adducts.

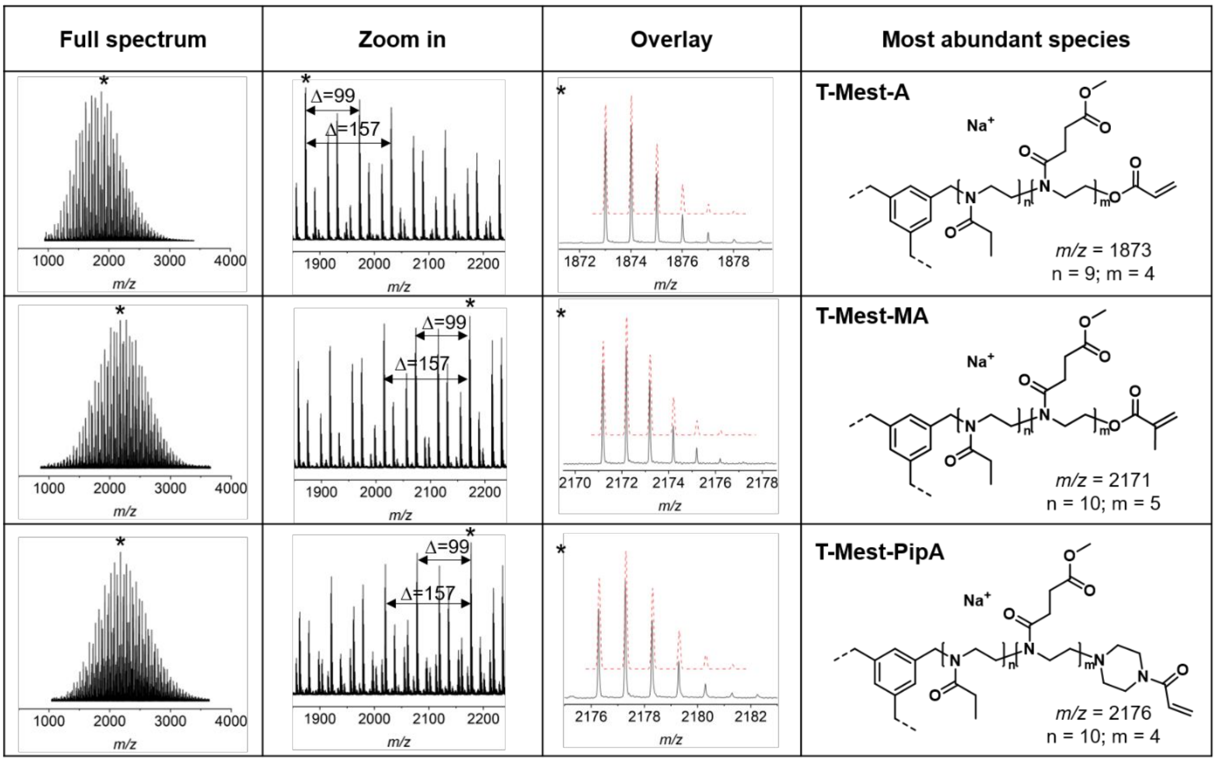

Figure S35. MALDI-TOF mass spectra (DCTB + NaTFA) of the crosslinkers derived from the copolymerization of EtOx and MestOx using the trifunctional initiator TIB. From left to right: Full spectra, zoom into an *m/z* region displaying the EtOx as well as the MestOx repeating units, overlay of the measured and calculated (red dotted line) isotopic pattern of the most abundant species (marked with *). All identified *m/z* species were found as sodium adducts.

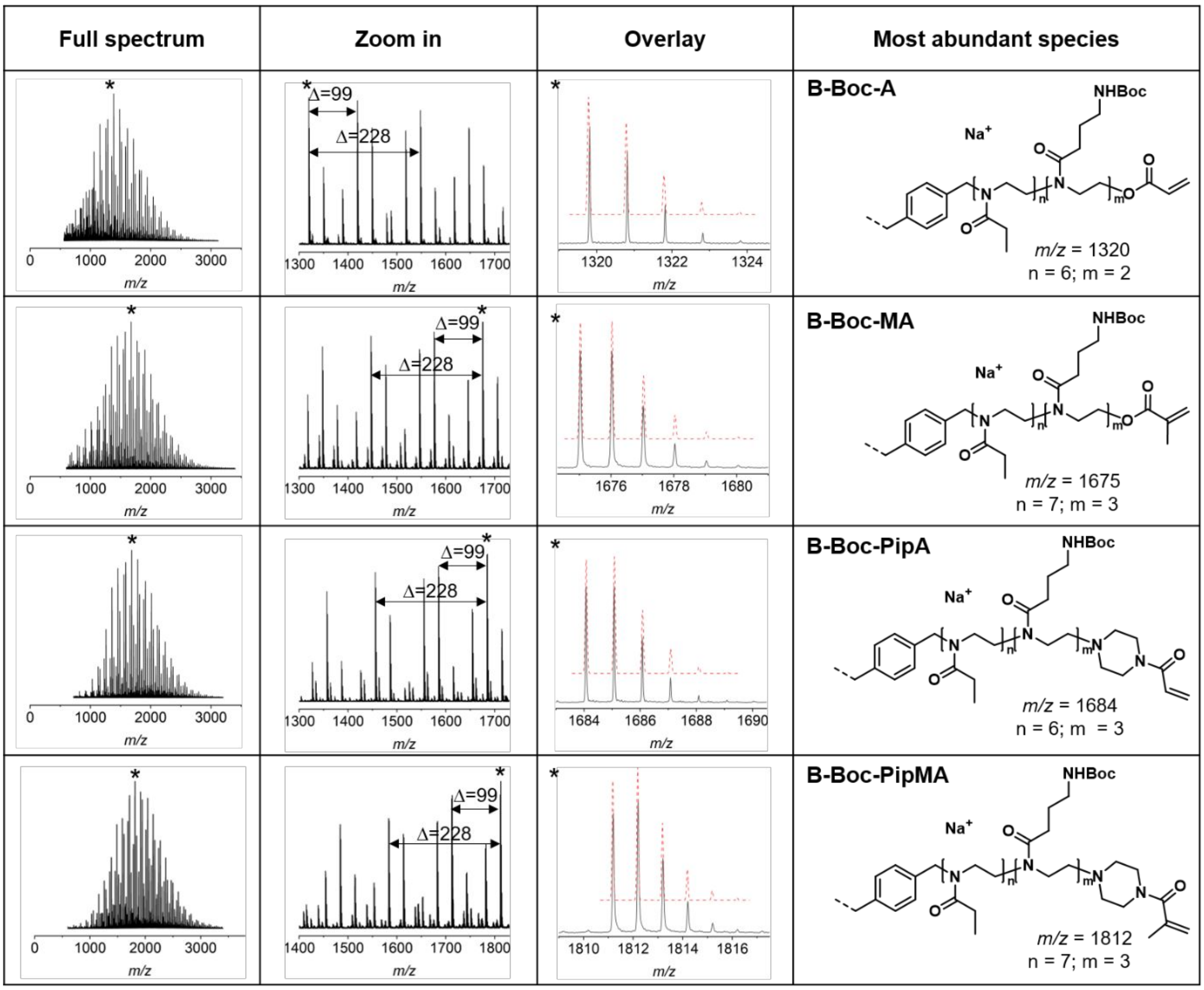

Figure S36. Full MALDI-TOF mass spectra (DCTB + NaTFA) of the crosslinkers derived from the copolymerization of EtOx and BocOx using the bifunctional initiator BIB. From left to right: Full spectra, zoom into an *m/z* region displaying the EtOx as well as the BocOx repeating units, overlay of the measured and calculated (red dotted line) isotopic pattern of the most abundant species (marked with *). All identified *m/z* species were found as sodium adducts.

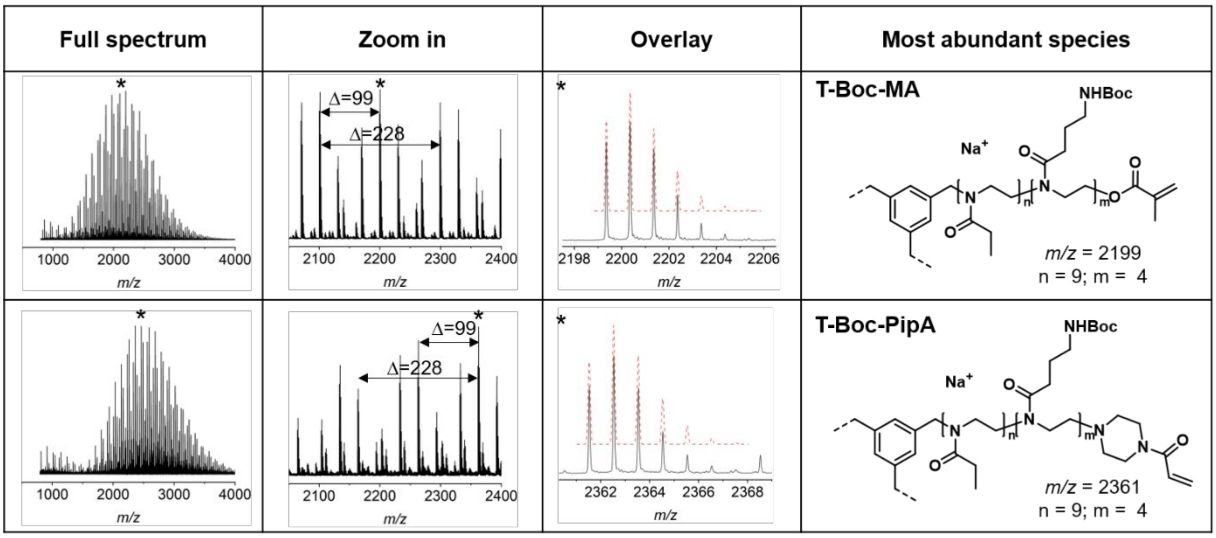

Figure S37. Full MALDI-TOF mass spectra (DCTB + NaTFA) of the crosslinkers derived from the copolymerization of EtOx and BocOx using the trifunctional initiator TIB. From left to right: Full spectra, zoom into an *m/z* region displaying the EtOx as well as the BocOx repeating units, overlay of the measured and calculated (red dotted line) isotopic pattern of the most abundant species (marked with *). All identified *m/z* species were found as sodium adducts.

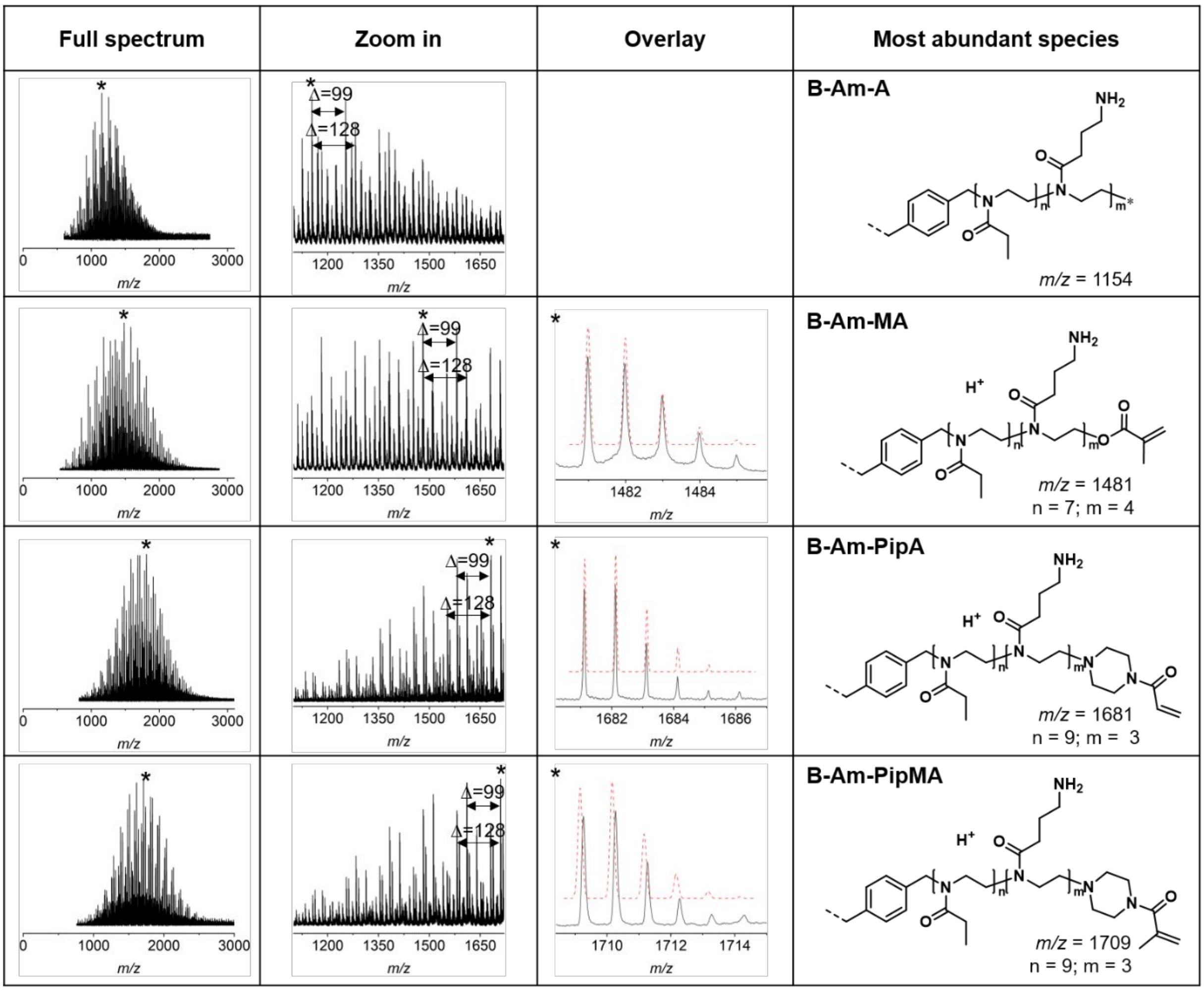

Figure S38. Full MALDI-TOF mass spectra (CHCA) derived from the deprotected B-BocOx resulted in the B-AmOx crosslinkers. From left to right: Full spectra, zoom into an *m/z* region displaying the EtOx as well as the AmOx repeating units, overlay of the measured and calculated (red dotted line) isotopic pattern of the most abundant species (marked with *). All identified *m/z* species were found as sodium adducts.

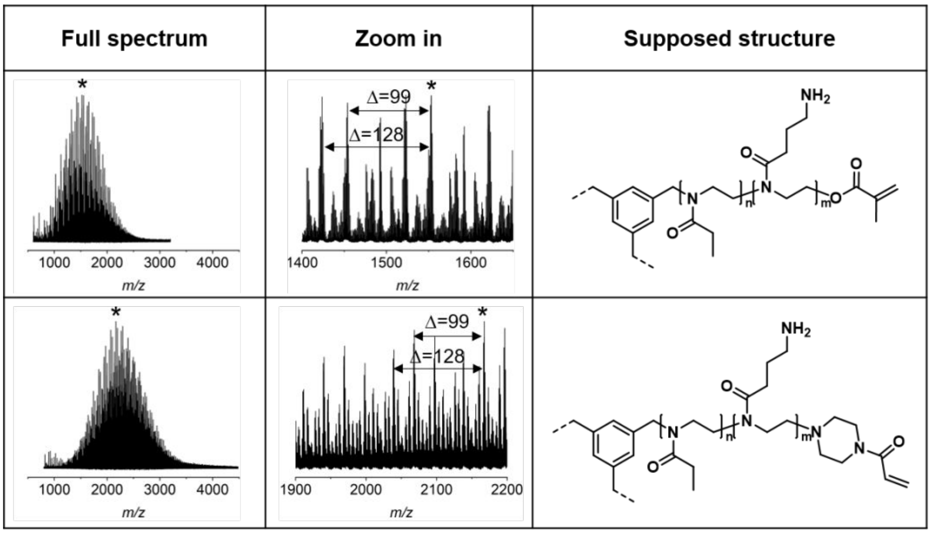

Figure S39. Full MALDI-TOF (CHCA) derived after deprotection from the deprotected T-BocOx resulted in the T-AmOx crosslinkers. From left to right: Full spectra, zoom into an *m/z* region displaying the EtOx as well as the AmOx repeating units, and supposed structure. The most abundant species is marked with *.

9.2 Cryogel analysis

9.2.1 Pore size analysis

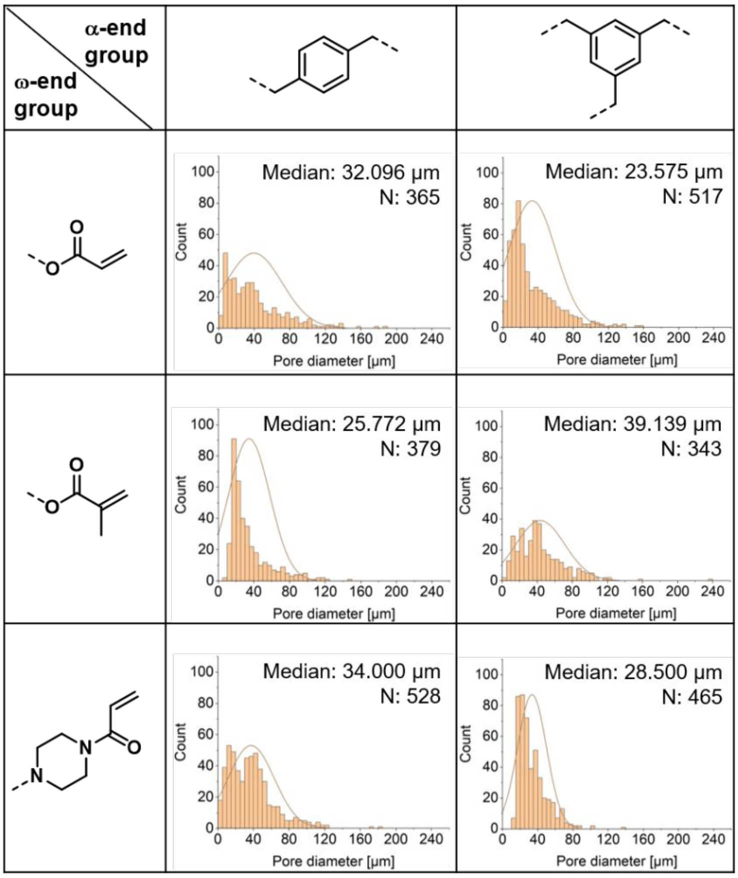

Figure S40. Schematic representation of the pore size evaluation based on the automated algorithmic analysis of SEM images.[7] Pores were automatically measured from 4 selected SEM images per cryogel sample.

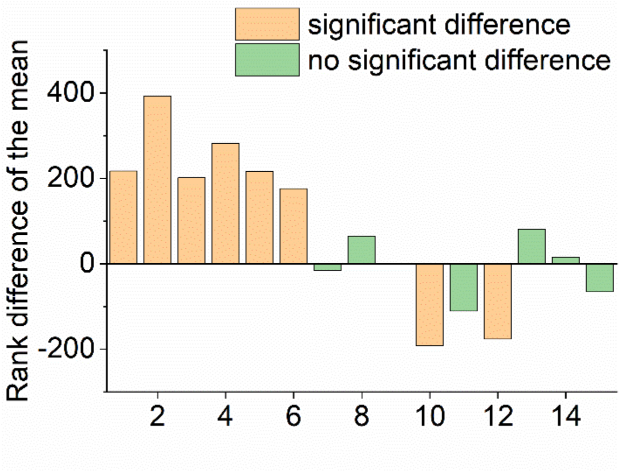

Figure S41. Rank differences of cryogel pore size comparisons according to Kruskal-Wallis analysis of variances (ANOVA) and Mood's median test. Both methods utilize Dunns test to compare differences between each group. Statistical significance was set at $p < 0.05$. An assignment of the numbers with the corresponding evaluated cryogel pairs are given in **Table S4**.

Table S4. Overview about the different cryogel pore size comparisons according to **Figure S4** from the Kruskal-Wallis analysis of variances (ANOVA) and Mood’s median test, utilizing Dunns test to compare difference between each group. Statistical significance was set at p<0.05.

Number	Group comparison	Significant difference	No significant difference
1	"CG-T-Et-MA" "CG-B-Et-A"	×	
2	"CG-T-Et-MA " "CG-B-Et-MA "	×	
3	"CG-T-Et-MA " "CG-B-Et-PipA"	×	
4	"CG-T-Et-MA " "CG-B-Et-MA"	×	
5	"CG-T-Et-MA " "CG-T-Et-PipA"	×	
6	"CG-B-Et-A " "CG-B-Et-MA "	×	
7	"CG-B-Et-A " "CG-B-Et-PipA"		×
8	"CG-B-Et-A " "CG-B-Et-MA "		×
9	"CG-B-Et-A " "CGT-Et-PipA"		×
10	"CG-B-Et-MA " "CG B-Et-PipA "	×	
11	"CG-B-Et-MA " “CG-B-Et-MA "		×
12	"CG-B-Et-MA " "CG-T-Et-PipA"	×	
13	"CG-B-Et-PipA" "CG-B-Et-MA "		×
14	"CG-B-Et-PipA " "CG-T-Et-PipA"		×
15	"CG-B-Et-MA " "CG-t-Et-PipA"		×

9.2.2 Swelling of EtOx-based cryogels

The swelling behavior of the EtOx-based cryogels was studied as described in literature[15] at room temperature by sampling a circular piece of the dry cryogel in a vial containing deionized water. At periodic time points (20 s, 40 s, 60 s, 300 s, 1800 s), the increase in the mass of the cryogel sample ($n = 3$, $n_{CG(T\text{-}Et\text{-}MA)} = 2$) was documented after the removal of the excess water that had adhered to the surface. The water uptake was calculated as the cryogel swelling ratio according to the following equation (1):

$$\text{CG swelling ratio} = \frac{m(CG)_{wet} - m(CG)_{dry}}{m(CG)_{dry}} \quad (1)$$

Where $m(CG)_{wet}$ and $m(CG)_{dry}$ are the weights of the cryogels in their swollen and dried states, respectively.

Table S5. Swelling degree measurements of all cryogels in water. Values represent the swelling ratios per cryogel at different time points (n = 3).

t [s]	CG-B-Et-A	CG-T-Et-A	CG-B-Et-MA	CG-T-Et-MA	CG-B-Et-PipA	CG-T-Et-PipA
0	0	0	0	0	0	0
20	6.001 ±0.240	4.795 ±0.395	27.346 ±1.544	22.754 ±0.418	22.740 ±1.794	22.752 ±0.325
40	16.636 ±2.428	13.753 ±1.229	28.014 ±0.378	23.005 ±0.161	22.606 ±1.371	23.103 ±0.524
60	25.836 ±1.695	17.046 ±2.295	27.860 ±0.536	23.097 ±0.420	22.922 ±1.201	23.129 ±0.645
600	26.756 ±0.457	18.728 ±0.607	27.785 ±0.849	22.585 ±0.173	22.999 ±1.398	23.236 ±0.482
1800	27.097 ±0.271	19.208 ±0.663	28.646 ±0.877	22.422 ±0.470	22.902 ±1.173	23.558 ±0.720

9.2.3 ^{13}C solid-state NMR

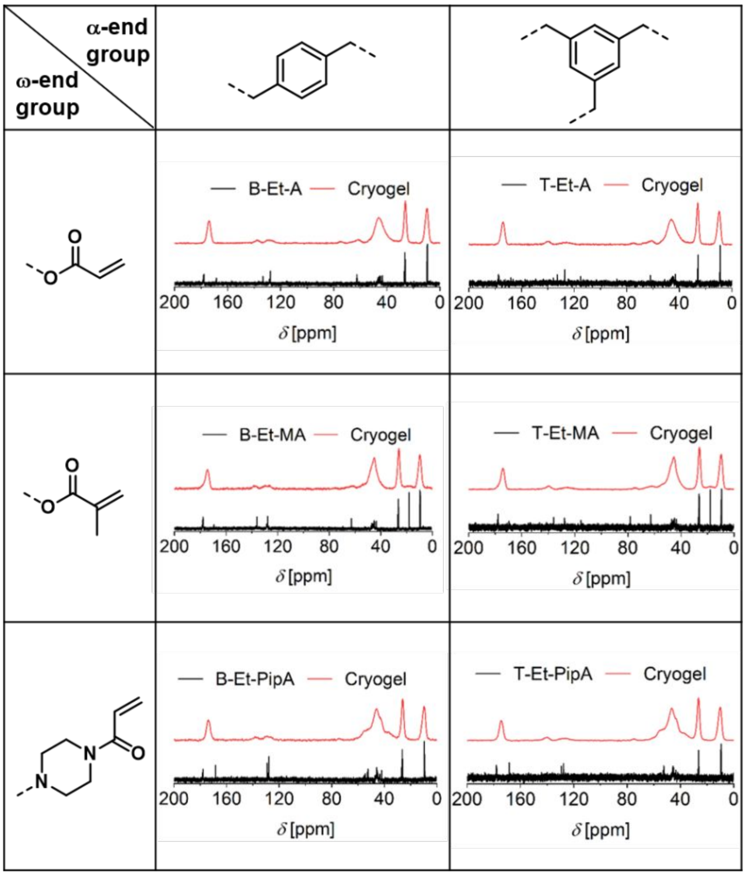

Figure S42. Overview about the results from ssNMR analysis of cryogels prepared from EtOx based crosslinkers with acrylate, methacrylate and **PipA** as ω-end groups. Overlays of the ssNMR cryogel spectra (red line) in comparison with the ^{13}C liquid NMR spectra of the respective EtOx-based crosslinkers (black line).

9.2.4 Thermogravimetric analysis

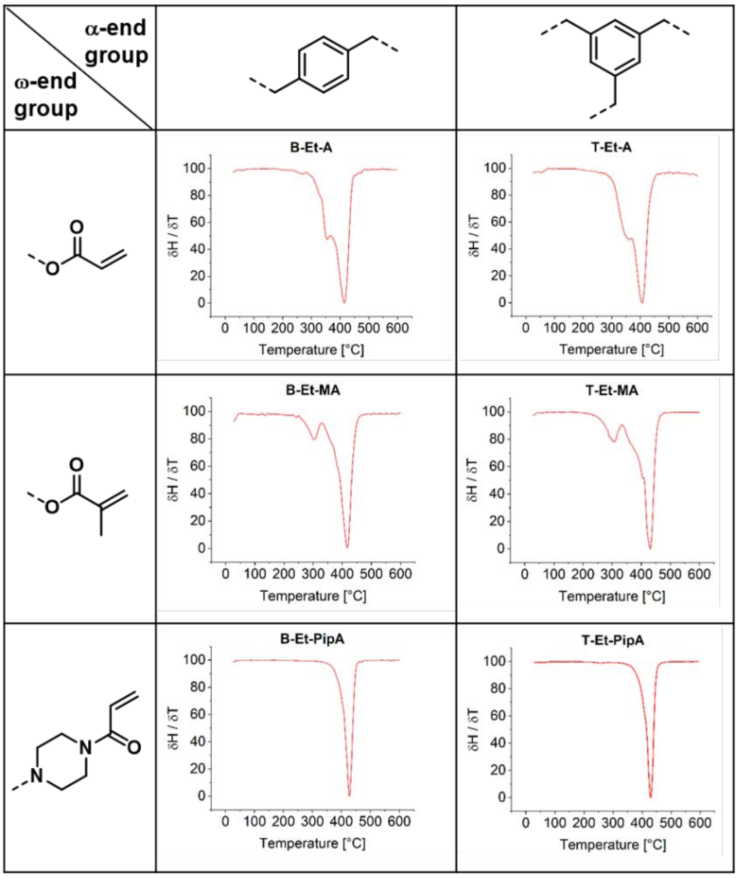

Figure S43. First derivatives of TGA degradation curves of cryogels based on B-EtOx and T-EtOx crosslinkers with acrylate, methacrylate and **PipA** as ω-end groups.

9.2.5 Stability tests

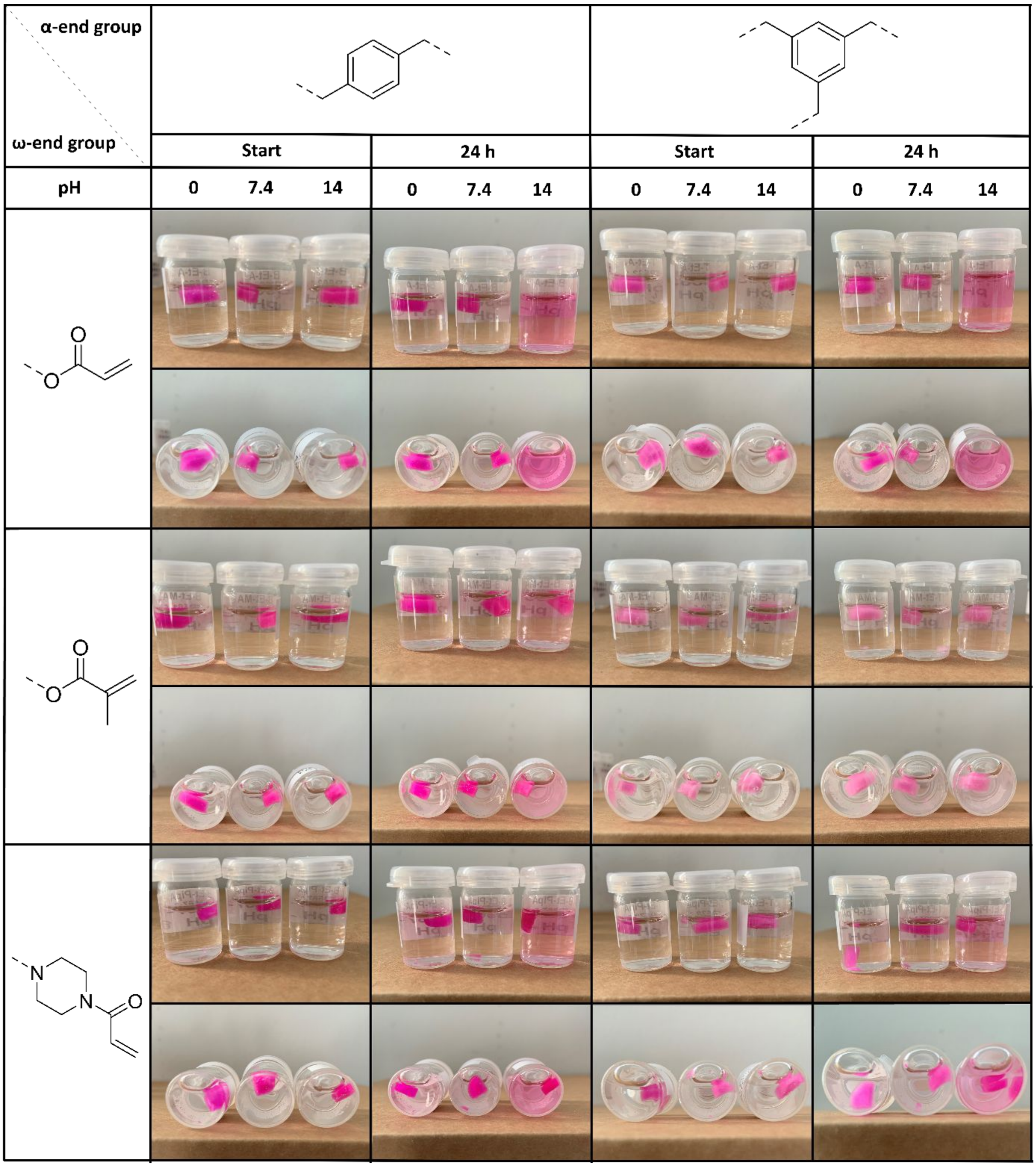

Figure S44. Overview about the stability tests of cryogels based on B-Et and T-Et crosslinkers with acrylate, methacrylate and **PipA** as ω-end group in aqueous solutions with different pH values (0, 7.4, 14). Pictures were taken at the beginning and after 24 h. Complete dissolution of the cryogel network was only visible in alkaline pH in case of acrylate ester based cryogels prepared based on **B-Et-A** and **T-Et-A** crosslinkers. The other cryogel systems remained stable.

10 REFERENCES

(1) Sheldrick, G. M. SHELXT - Integrated space-group and crystal-structure determination. *Acta Crystallogr. Sect. A* **2015**, *71*, 3-8.

(2) Sheldrick, G. M. Crystal structure refinement with SHELXL. *Acta Crystallogr. Sect. C-Struct. Chem.* **2015**, *71*, 3-8.

(3) Dolomanov, O. V.; Bourhis, L. J.; Gildea, R. J.; Howard, J. A. K.; Puschmann, H. OLEX2: A complete structure solution, refinement and analysis program. *J. Appl. Crystallogr.* **2009**, *42*, 339-341.

(4) Schneider, C. A.; Rasband, W. S.; Eliceiri, K. W. NIH image to imageJ: 25 years of image analysis. *Nat. Methods* **2012**, *9*, 671-675.

(5) Schindelin, J.; Arganda-Carreras, I.; Frise, E.; Kaynig, V.; Longair, M.; Pietzsch, T.; Preibisch, S.; Rueden, C.; Saalfeld, S.; Schmid, B.; et al. Fiji: an open-source platform for biological-image analysis. *Nat. Methods* **2012**, *9*, 676-682.

(6) Gerst, R.; Cseresnyés, Z.; Figge, M. T. JIPipe: visual batch processing for ImageJ. *Nat. Methods* **2023**, *20*, 168-169.

(7) Behrendt, F.; Cseresnyés, Z.; Gerst, R.; Gottschaldt, M.; Figge, M. T.; Schubert, U. S. Evaluation of reproducible cryogel preparation based on automated image analysis using deep learning. *J. Biomed. Mater. Res. A* **2023**, *111*, 1734-1749.

(8) Stringer, C.; Wang, T.; Michaelos, M.; Pachitariu, M. Cellpose: a generalist algorithm for cellular segmentation. *Nat. Methods* **2021**, *18*, 100-106.

(9) Celebi, O.; Barnes, S. R.; Narang, G. S.; Kellogg, D.; Mecham, S. J.; Riffle, J. S. Molecular weight distribution and endgroup functionality of poly(2-ethyl-2-oxazoline) prepolymers. *Polymer* **2015**, *56*, 147-156.

(10) Hermann, K.; Pratumyot, Y.; Polen, S.; Hardin, A. M.; Dalkilic, E.; Dastan, A.; Badjic, J. D. Twisted baskets. *Chem. Eur. J.* **2015**, *21*, 3550-3555.

(11) Bouten, P. J. M.; Hertsen, D.; Vergaelen, M.; Monnery, B. D.; Boerman, M. A.; Goossens, H.; Catak, S.; van Hest, J. C. M.; Van Speybroeck, V.; Hoogenboom, R. Accelerated living cationic ring-opening polymerization of a methyl ester functionalized 2-oxazoline monomer. *Polym. Chem.* **2015**, *6*, 514-518.

(12) Hartlieb, M.; Pretzel, D.; Kempe, K.; Fritzsche, C.; Paulus, R. M.; Gottschaldt, M.; Schubert, U. S. Cationic poly(2-oxazoline) hydrogels for reversible DNA binding. *Soft Matter* **2013**, *9*, 4693-4704.

(13) Baowei, Y.; Yicheng, M.; Huiling, T.; Meng, Y.; Song, F.; Ying, H. Synthesis and characterization of glucose-sensitive phenylboronic acid derivative. CN112979685A, **2021**.

(14) Sari, S.; Yilmaz, M. Synthesis, characterization, acetylcholinesterase inhibition, and molecular docking studies of new piperazine substituted dihydrofuran compounds. *Med. Chem. Res.* **2020**, *29*, 1804-1818.

(15) Boonkanon, C.; Phatthanawiwat, K.; Chuenchom, L.; Lamthornkit, N.; Taweekarn, T.; Wongniramaikul, W.; Choodum, A. Preparation and characterization of calcium cross-linked starch monolithic cryogels and their application as cost-effective green filters. In *Polymers*, 2021; Vol. 13.
